# Synthesis and Evaluation of Novel 1,2,6-Thiadiazinone Kinase Inhibitors as Potent Inhibitors of Solid Tumors

**DOI:** 10.3390/molecules26195911

**Published:** 2021-09-29

**Authors:** Andreas S. Kalogirou, Michael P. East, Tuomo Laitinen, Chad D. Torrice, Kaitlyn A. Maffuid, David H. Drewry, Panayiotis A. Koutentis, Gary L. Johnson, Daniel J. Crona, Christopher R. M. Asquith

**Affiliations:** 1 Department of Life Sciences, School of Sciences, European University Cyprus, 6 Diogenis Str., Engomi, P.O. Box 22006, Nicosia 1516, Cyprus; 2 Department of Chemistry, University of Cyprus, P.O. Box 20537, Nicosia 1678, Cyprus; koutenti@ucy.ac.cy; 3 Department of Pharmacology, School of Medicine, University of North Carolina, Chapel Hill, NC 27599, USA; Michael_East@med.unc.edu (M.P.E.); gary_johnson@med.unc.edu (G.L.J.); 4 School of Pharmacy, Faculty of Health Sciences, University of Eastern Finland, 70211 Kuopio, Finland; tuomo.laitinen@uef.fi; 5 Division of Pharmacotherapy and Experimental Therapeutics, Eshelman School of Pharmacy, University of North Carolina, Chapel Hill, NC 27599, USA; chad_torrice@med.unc.edu (C.D.T.); kmaffuid@email.unc.edu (K.A.M.); crona@email.unc.edu (D.J.C.); 6 Structural Genomics Consortium, Eshelman School of Pharmacy, University of North Carolina, Chapel Hill, NC 27599, USA; david.drewry@unc.edu; 7 Lineberger Comprehensive Cancer Center, School of Medicine, University of North Carolina, Chapel Hill, NC 27599, USA

**Keywords:** thiadiazinone, bladder cancer, prostate cancer, pancreatic cancer, breast cancer, chordoma, lung cancer

## Abstract

A focused series of substituted 4*H*-1,2,6-thiadiazin-4-ones was designed and synthesized to probe the anti-cancer properties of this scaffold. Insights from previous kinase inhibitor programs were used to carefully select several different substitution patterns. Compounds were tested on bladder, prostate, pancreatic, breast, chordoma, and lung cancer cell lines with an additional skin fibroblast cell line as a toxicity control. This resulted in the identification of several low single digit micro molar compounds with promising therapeutic windows, particularly for bladder and prostate cancer. A number of key structural features of the 4*H*-1,2,6-thiadiazin-4-one scaffold are discussed that show promising scope for future improvement.

## 1. Introduction

**Scheme 1:**
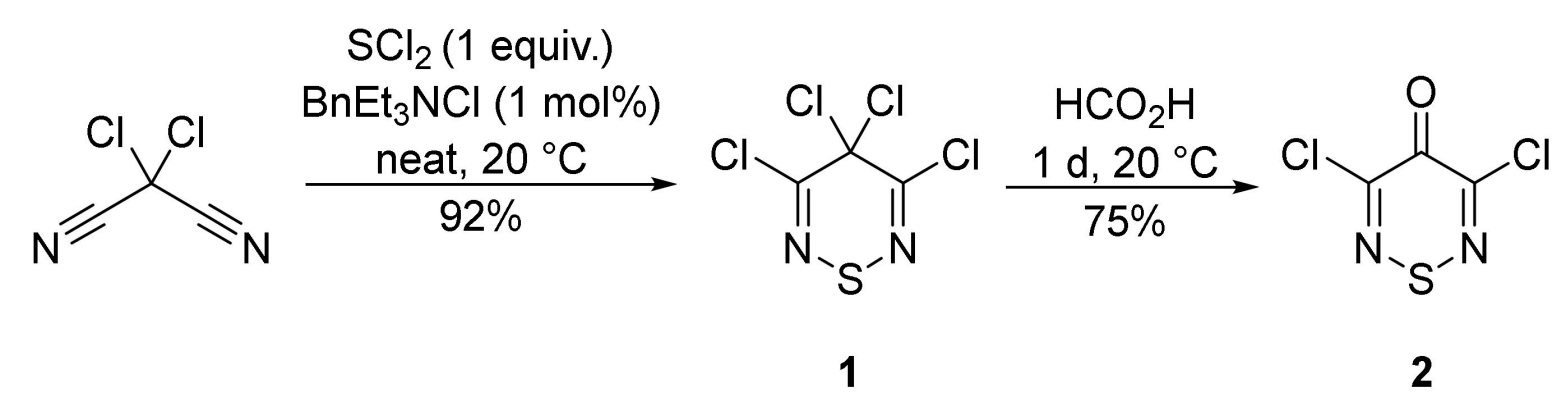
Route to access 3,5-dichloro-4*H*-1,2,6-thiadiazin-4-one (**2**).

Heteroatom-rich scaffolds are underexplored in medicinal chemistry, potentially leading to missed opportunities [1,2,3,4,5,6]. One such ring system is the non S-oxidized 4*H*-1,2,6-thiadiazinone, selected examples of which have applications as plant protectants [7,8,9,10,11], organic photovoltaics (OPVs) [12,13], liquid crystals [14], and potential anti-cancer agents [15]. The precursor to this 1,2,6-thiadiazinone scaffold, is the 3,4,4,5-tetrachloro-4*H*-1,2,6-thiadiazine (**1**) which was first reported in 1973 [16]. Subsequently, the chemistry was later developed to allow broad diversification [17]. Synthetic interest in thiadiazones/thiadiazines is currently focused around two scaffolds: 3,5-dichloro-4*H*-1,2,6-thiadiazin-4-one (**2**) and its precursor, 3,4,4,5-tetrachloro-4*H*-1,2,6-thiadiazine (**1**), which can be derived from dichloromalononitrile (Scheme 1) [18]. The substitution chemistry of both chlorines of 3,5-dichloro-4*H*-1,2,6-thiadiazin-4-one (**2**) has been explored in detail and affords a versatile platform to prepare suitable 3,5-substituted derivatives.

**Figure 1:**
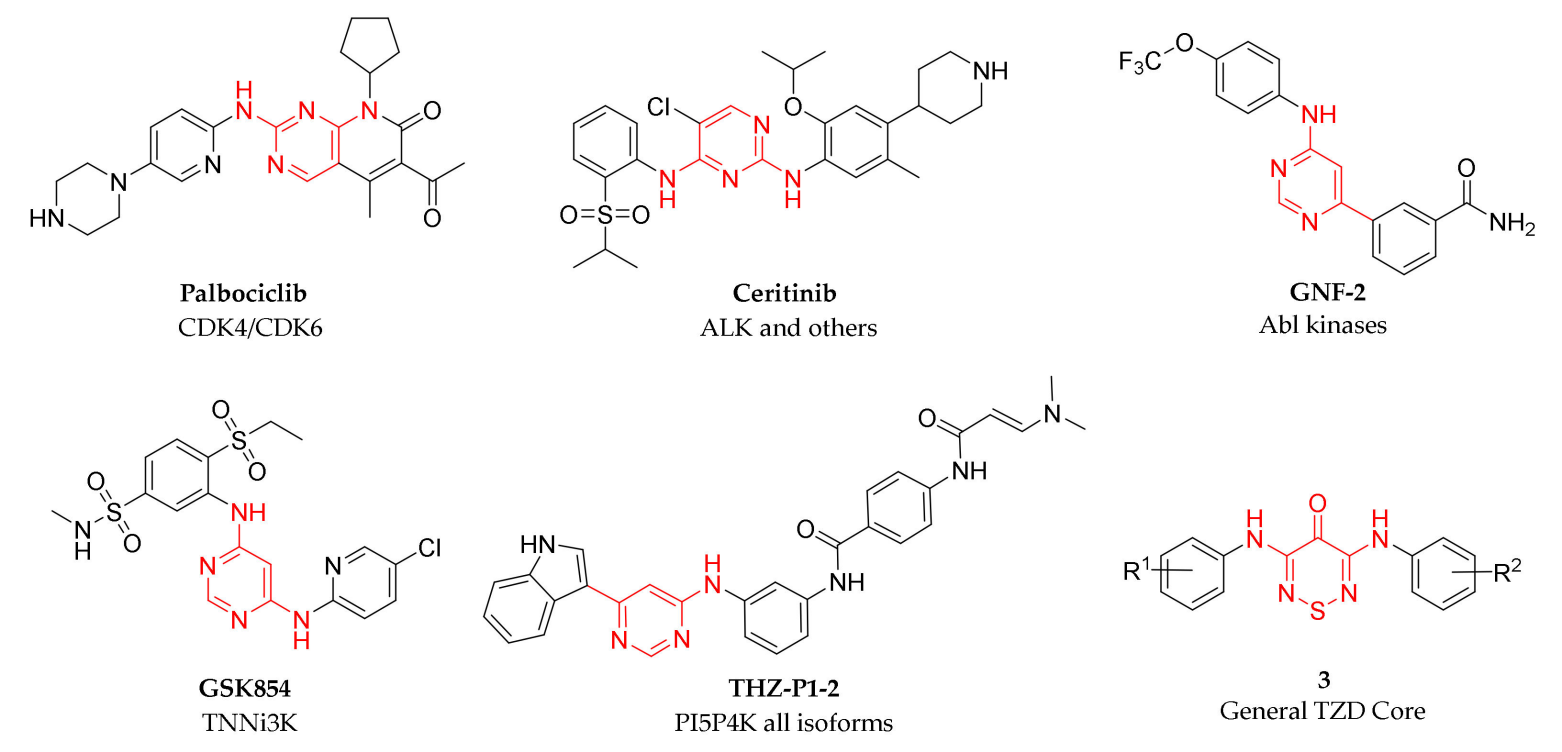
Representative examples of known anilinopyrimidines (highlighted in red) and general 1,2,6-thiadiazinone core (**3**).

3-Anilino-1,2,6-thiadiazinones occupy a similar chemical space to more commonly used medicinal chemistry scaffolds, including the mono- and di-anilinopyrimidines. The anilinopyrimidines are a common chemotype found in ~10% of the clinically approved kinase inhibitor drugs, including palbociclib and ceritinib (Figure 1) [19]. Furthermore, anilinopyrimidines are found in a number of tool compounds for kinase interrogation, including GNF-2 for ABL [20], GSK854 for TNNi3K [21,22], and THZ-P1-2 for the PI5P4K isoforms [23]. The broad activity profile of the dianilinopyrimidine chemotype can also be seen within several kinase inhibitor screening sets, including PKIS, PKIS2, and KCGS, with more than 35 examples showing activity on >400 different protein kinases (excluding mutants) in either enzyme inhibition or affinity capture assays [24,25,26].

**Scheme 2:**
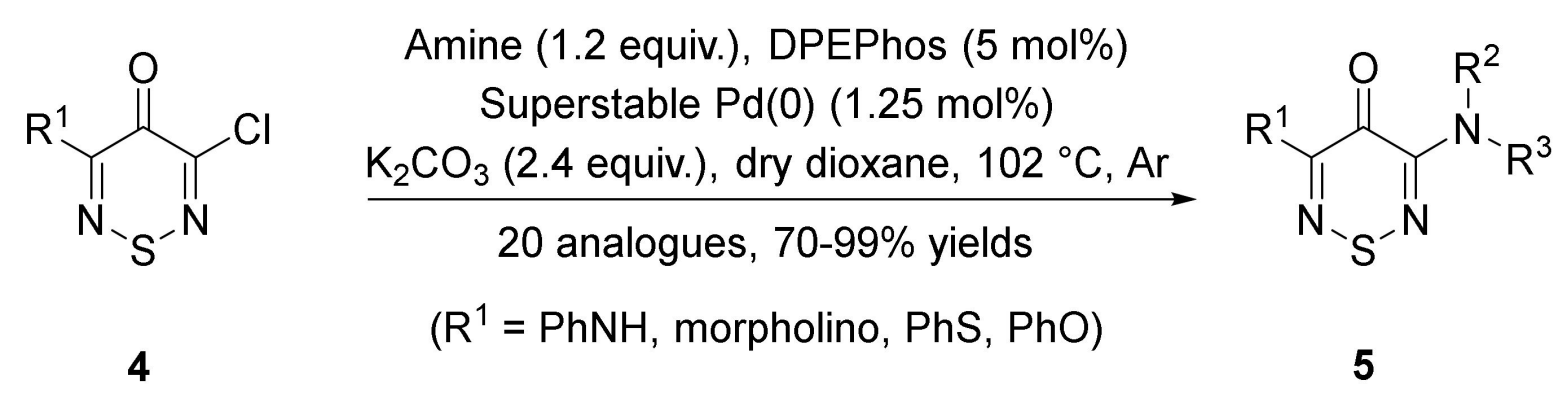
C-N coupling of 5-subsituted 3-chlorothiadiazinones 4 with (het)arylamines.

The chemistry of 3,5-dichloro-4*H*-1,2,6-thiadiazin-4-one (**2**) is dominated by the substitution of the two chlorides. In particular, substitution can occur with N, O, and S nucleophiles as well as with organometallic reagents (carbon nucleophiles). Interestingly, while the synthesis of symmetrical 3,5-diarylthiadiazines is easy via Suzuki and Stille reactions [27], the synthesis of unsymmetrical analogues is more difficult as selective reaction of one of the two sites is not possible [28]. However, with amine or alkoxide nucleophiles, a stepwise reaction is possible as the first displacement is considerably more facile that the second one [17,18]. This allows for the preparation of unsymmetrical 3,5-diaminothiadiazines or even 3-amino-5-aryl derivatives via a combination of nucleophilic attack by an amine followed by a Suzuki reaction in the remaining C-5 position [15]. Moreover, a palladium-catalyzed C-N coupling protocol was developed for the more difficult amino-displacement of the second chloride of thiadiazinone **4** to afford disubstituted derivatives **5** that avoids the use of elevated temperatures and excess amine (Scheme 2) [29].

## 2. Results

### 2.1. Synthesis

**Figure 2:**
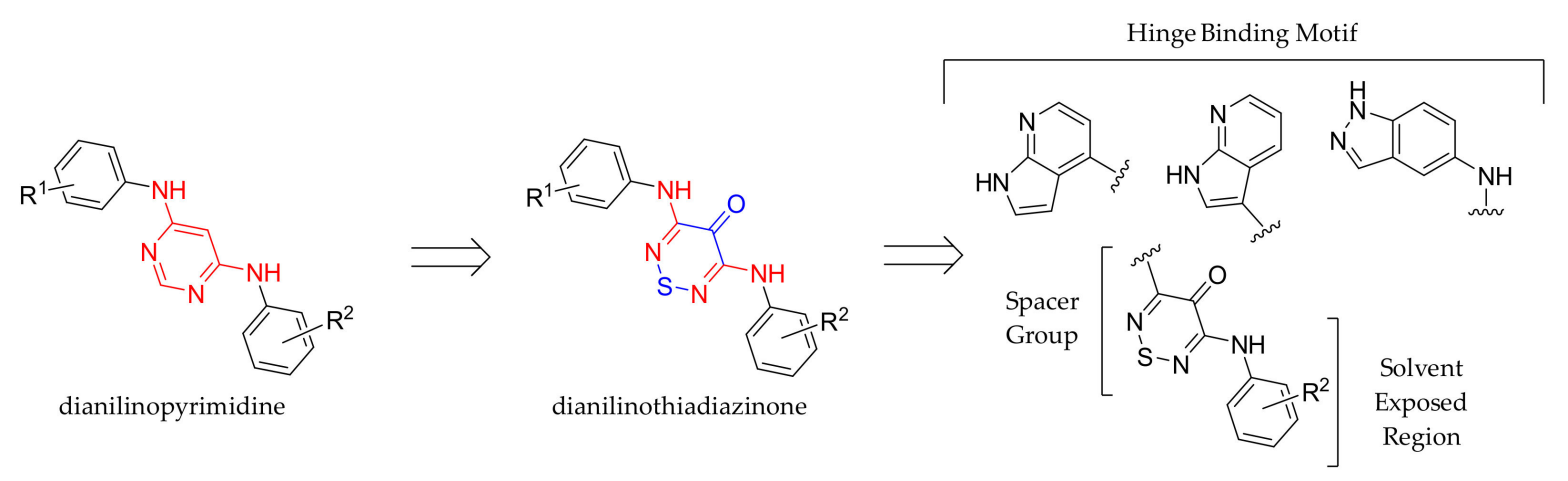
Design rational of novel 1,2,6-thiadiazinone analogues.

An in-depth analysis of the kinome-wide profiling of the dianilinopyrimidines in PKIS, PKIS2, and KCGS, and within the literature, led us to select a series of R^1^ and R^2^ substituent patterns (Figure 2, Table 1) that showed the broadest range of activity on human kinases. These compounds were designed to capture as much of the kinome as possible and, while unoptimized, they should provide attractive starting points for future work. In addition, several powerful hinge binding motifs were selected to enhance the scaffolds potential to capture extra kinases [30,31,32].

**Scheme 3:**
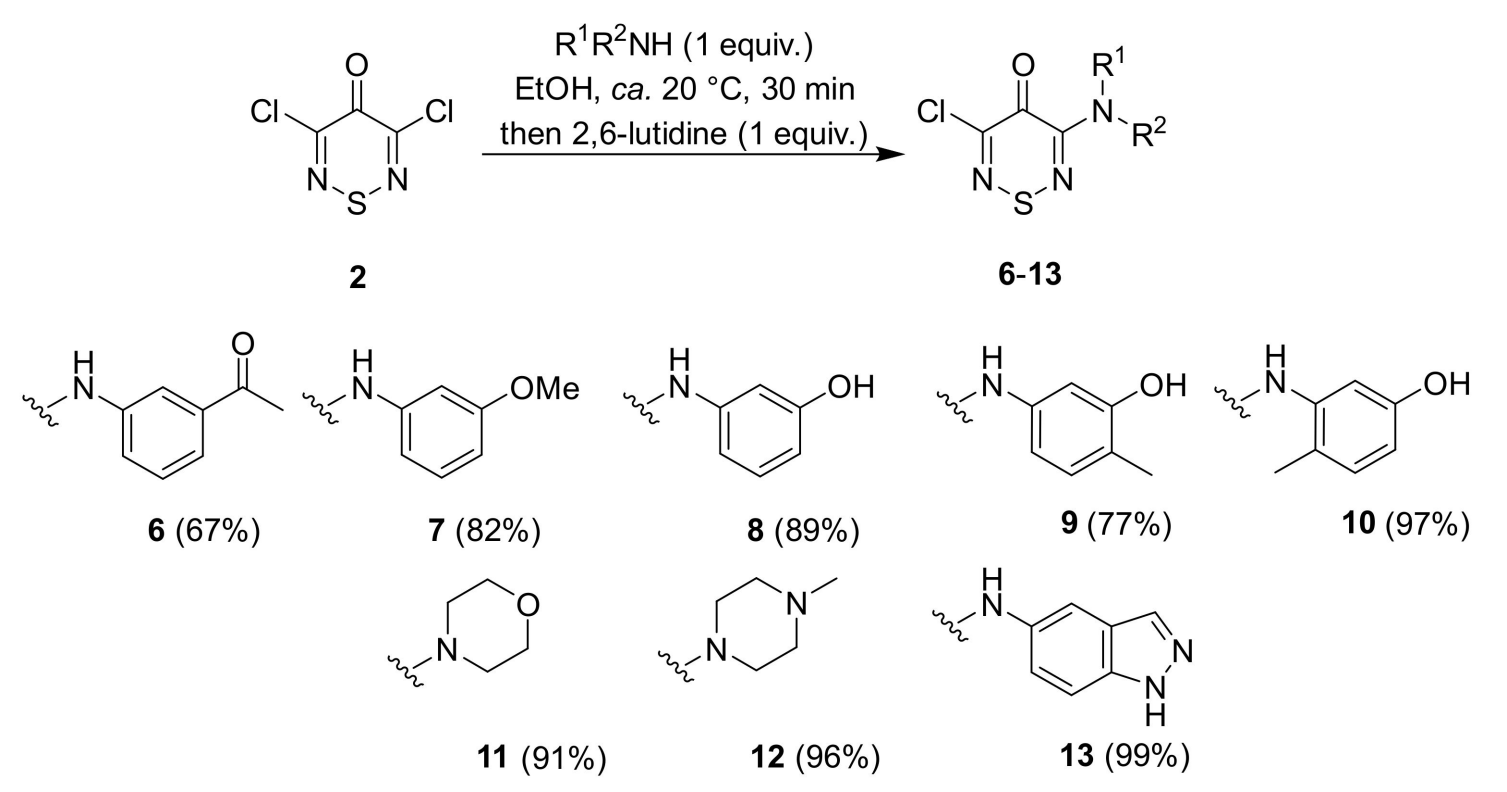
Synthetic route to access 5-amino-3-chloro-4*H*-1,2,6-thiadiazin-4-ones **6**–**13**.

The corresponding 1,2,6-thiadiazin-4-ones (**14**–**19**, **21**–**25**) were synthesized in two-steps starting from 3,5-dichloro-4*H*-1,2,6-thiadiazin-4-one (**2**) [18]. The first chloride of thiadiazinone **2** can be readily displaced by anilines and alkylamines by reaction with stoichiometric amounts of the amine (1 equiv.) and the base 2,6-lutidine (1 equiv.) in EtOH, at ca. 0–20 °C. The required 5-amino-3-chloro-4*H*-1,2,6-thiadiazin-4-ones **6**–**13** were isolated in 67–99% yields with a chromatography-free work-up (Scheme 3).

**Scheme 4:**
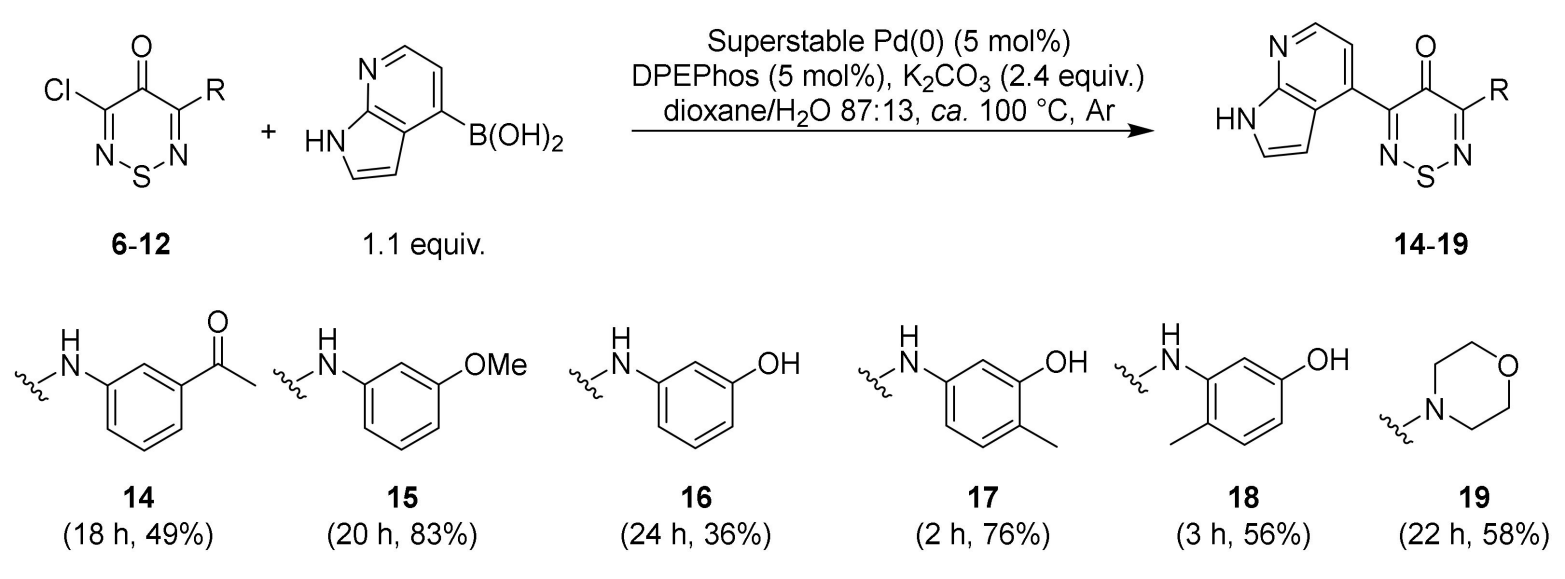
Suzuki couplings of thiadiazines **6**–**12** with (1*H*-pyrrolo[2,3-*b*]pyridin-4-yl)boronic acid to afford disubstituted products **14**–**19**.

Subsequently, scaffolds **6**–**12** were subjected to a Suzuki coupling with (1*H*-pyrrolo [2,3-*b*]pyridin-4-yl)boronic acid. The seven desired products **14**–**19** were obtained in moderate to good yields (36–83%) (Scheme 4).

**Scheme 5:**
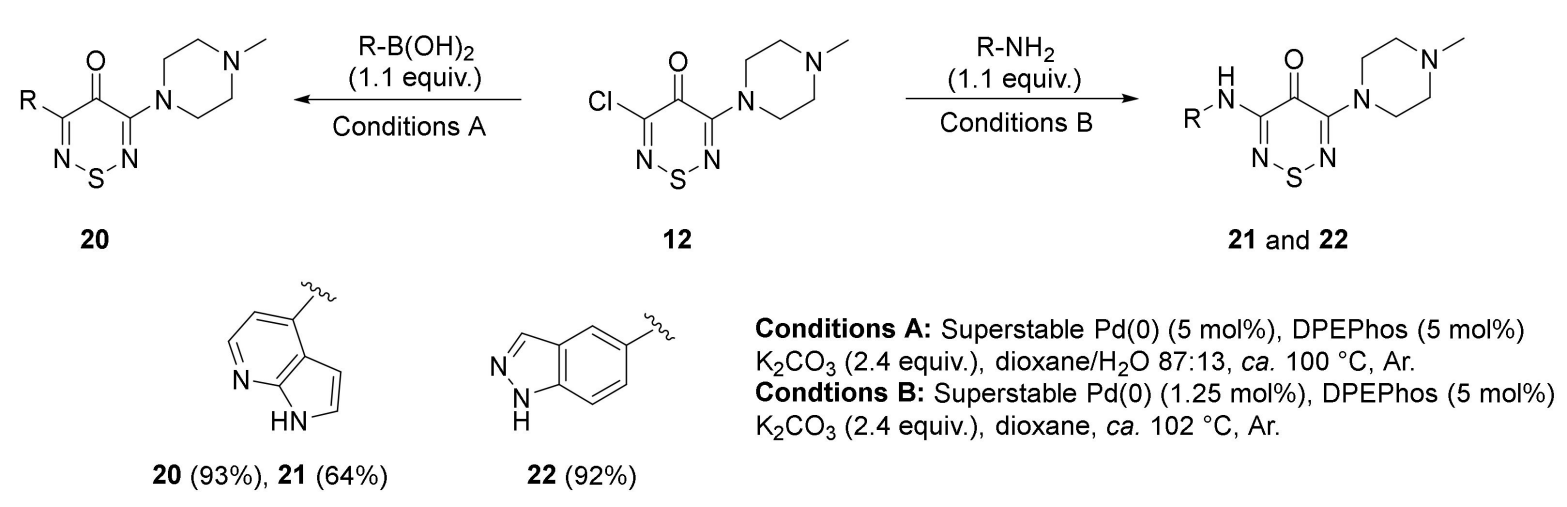
Scaffold diversification with tertiary amine substituent to afford **20**–**22**.

A similar Suzuki coupling reaction was used to prepare 3-(4-methylpiperazin-1-yl)-5-(1*H*-pyrrolo[2,3-*b*]pyridin-4-yl)-4*H*-1,2,6-thiadiazin-4-one (**20**) starting from 3-chloro-5-(4-methylpiperazin-1-yl)-4*H*-1,2,6-thiadiazin-4-one (**12**) and (1*H*-pyrrolo[2,3-*b*]pyridin-4-yl)boronic acid (Scheme 5). Moreover, the two 3,5-diamino-substituted thiadiazines **21** and **22** were prepared via our previously developed C-N coupling methodology [15] from 5-(4-methylpiperazin-1-yl)thiadiazin-4-one **12**. The products were isolated in 64 and 92% yields, respectively (Scheme 5).

**Scheme 6:**
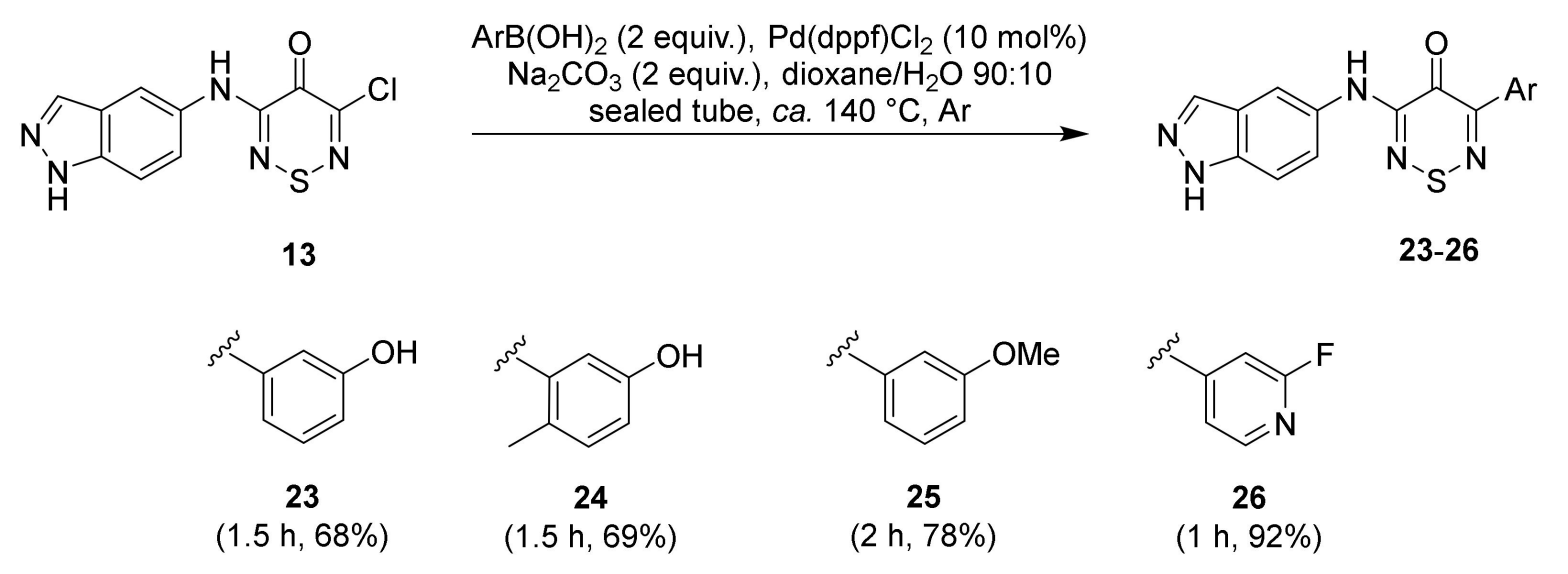
Suzuki couplings of 5-chloro-3-[(1*H*-indazol-5-yl)amino]-4*H*-1,2,6-thiadiazin-4-one (**13**).

Finally, a modified Suzuki coupling procedure was required for the synthesis of the 3-[(1*H*-indazol-5-yl)amino]-4*H*-1,2,6-thiadiazin-4-ones. A brief optimization showed that the combination of the catalyst Pd(dppf)Cl_2_ (10 mol%) and the base Na_2_CO_3_ (2 equiv.), in dioxane/H_2_O 90:10, in a sealed tube at ca. 140 °C was required to drive the reactions to completion. The four desired products **23**–**26** were obtained in medium to good yields of 68–92% (Scheme 6).

It is important to note that the stability of dianilinothiadiazinones in biological systems has previously been assessed [15]. 3,5-Bis(phenylamino)-4*H*-1,2,6-thiadiazin-4-one [18] is stable to neutral, acidic, or slightly basic aqueous conditions, the presence of amine or thiol nucleophiles, and oxidizing and reducing conditions. We similarly tested thiadiazinone **16** from our current study and found the same results.

### 2.2. Cancer Cell Screening

Compounds **14**–**26** were screened on an array of cancer cell lines to explore the structural requirements for anti-cancer activity within the 1,2,6-thiadiazinone scaffold (Table 1). This included pancreatic, bladder, prostate, breast, chordoma, and lung cancer cell lines while a skin fibroblast cell line was used as a toxicity control [33,34,35,36]. These cancer cell lines present different drug resistance profiles and when combined in a panel, each represents a distinct therapeutic challenge to overcome [37].

The first analogue 3-[(3-acetylphenyl)amino]-5-(1*H*-pyrrolo[2,3-*b*]pyridin-4-yl)-4*H*-1,2,6-thiadiazin-4-one (**14**) demonstrated limited inhibition across the panel of cell lines with the most inhibition shown on the bladder cancer cell line (IC_50_ = 15 μM). Switching from the acetyl substitution **14** to the methoxy **15** showed no improvement across the panel. Removal of the methyl on the methoxy **15**, to afford a hydroxy functionality **16**, led to a 10-fold increase in potency on the bladder cancer cell line (IC_50_ = 1.6 μM) with no toxicity in the WS-1 cell line (IC_50_ = >100 μM). The introduction of a 4-methyl substituent **17** removed weak inhibition on PANC1 and UCH-1 but did not show any improvement on the other cell lines in the panel. Interestingly, the introduction of a 2-methyl substituent **18** removed nearly all anti-cancer activity. Switching to the aliphatic a morpholine substituent again changed the preference toward pancreatic (IC_50_ = 11 μM) and prostate (IC_50_ = 14 μM) cancers with little activity towards bladder and the other cell lines.

We then sought to look at changing the hinge binding motif using the methyl piperazine as the fixed substituent. This tertiary amine substitution affords compounds with favorable properties [38,39,40,41,42]. However, in the case of our three powerful hinge binders [30], we observed very limited activity across both azaindoles, 4-postion **20** and 6-position **21**, along with the indazole **22**. The indazole **22** showed a small amount of toxicity in the WS-1 cell line (IC_50_ = 24 μM).

Switching to the indazole scaffold afforded a different potential hinge binding orientation. 5-(3-Hydroxyphenyl)-3-[(1*H*-indazol-5-yl)amino]-4*H*-1,2,6-thiadiazin-4-one (**23**) had only weak activity in the panel of cell lines but also showed no toxicity on the WS-1 cell line (IC_50_ = >100 μM). The introduction of a 2-methyl group to compound **23** to afford **24** improved the overall anti-cancer profile of the scaffold, with the most potency observed on breast cancer cell line (IC_50_ = 13 μM) and no toxicity on WS-1. The introduction of the methoxy group at the 3-position of thiadiazinone **25** changed the anti-cancer profile significantly with increased potency against bladder cancer (IC_50_ = 13 μM). Finally, we tested an isosteric replacement for the 3-methoxy the 3-fluoro and introduced a 4-pyridyl substitution pattern to afford compound **26**; this substituent orientation is observed in a number of kinase tool compounds [43,44]. 5-(2-Fluoropyridin-4-yl)-3-[(1*H*-indazol-5-yl)amino]-4*H*-1,2,6-thiadiazin-4-one (**26**) demonstrated potent activity against both bladder (IC_50_ = 8.4 μM) and prostate (IC_50_ = 5.7 μM) cancer with no toxicity on the WS-1 cell line (IC_50_ = >100 μM).

### 2.3. Kinome Profiling of Thiadiazinones ***16***, ***17***, and ***26***

**Figure 3:**
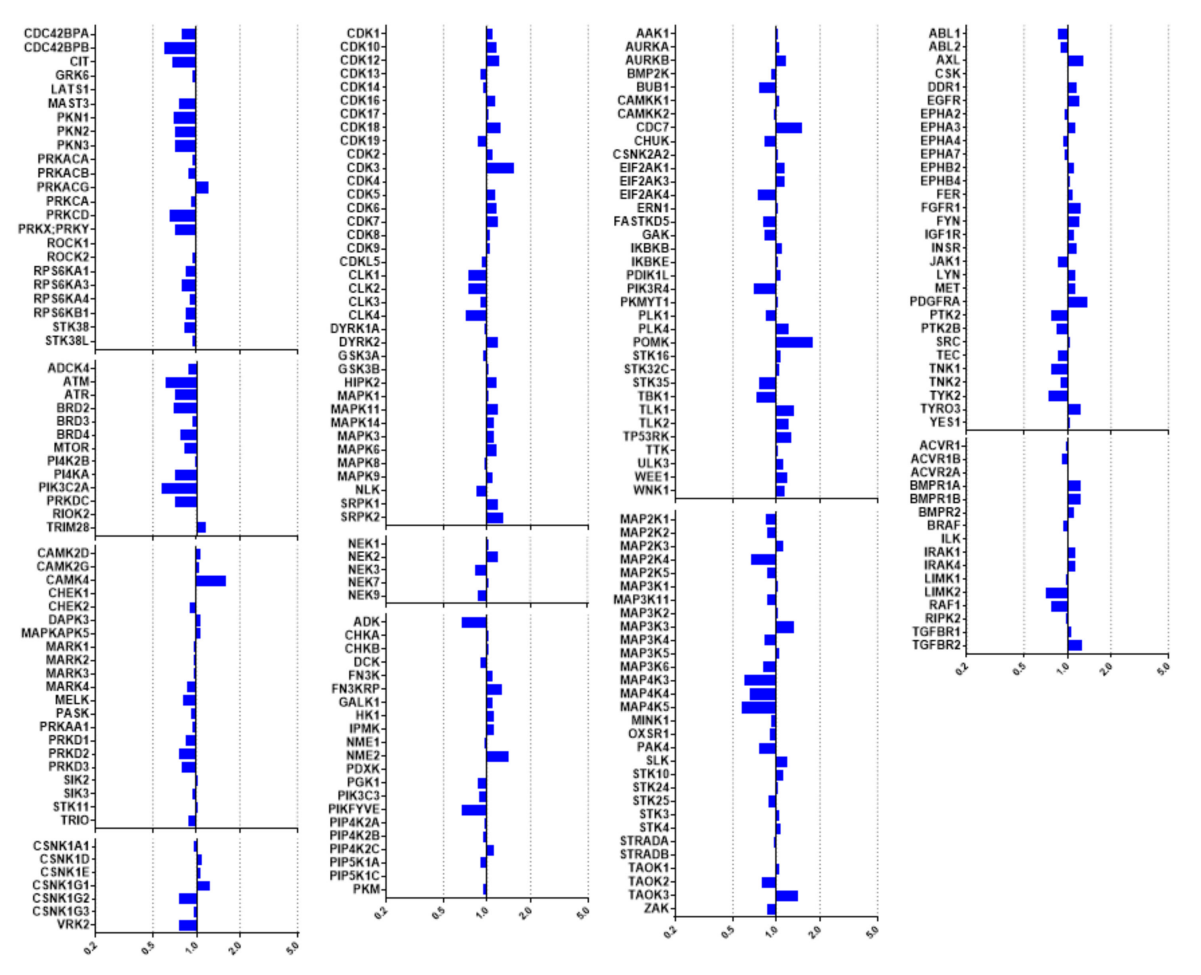
MIBS kinome profiling for thiadiazinone **16** (1 μM)/DMSO.

**Figure 4:**
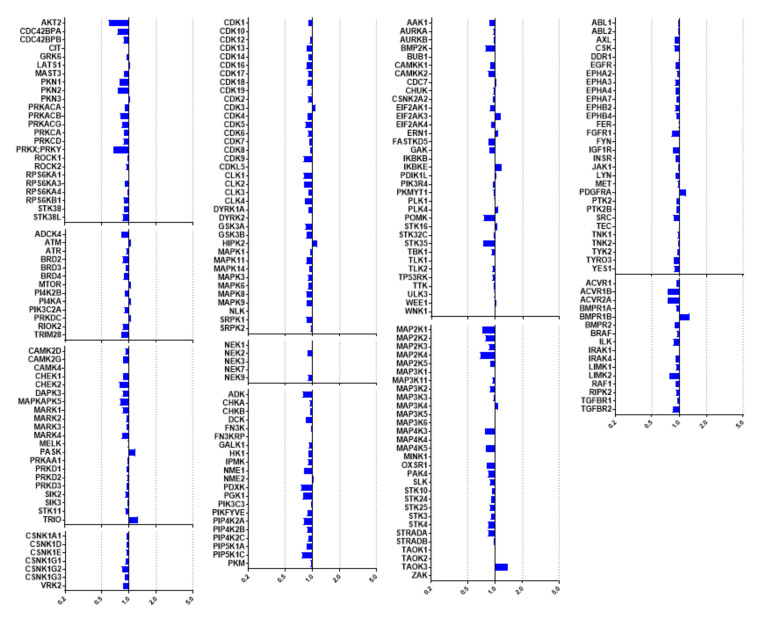
MIBS kinome profiling for thiadiazinone **17** (1 μM)/DMSO.

**Figure 5:**
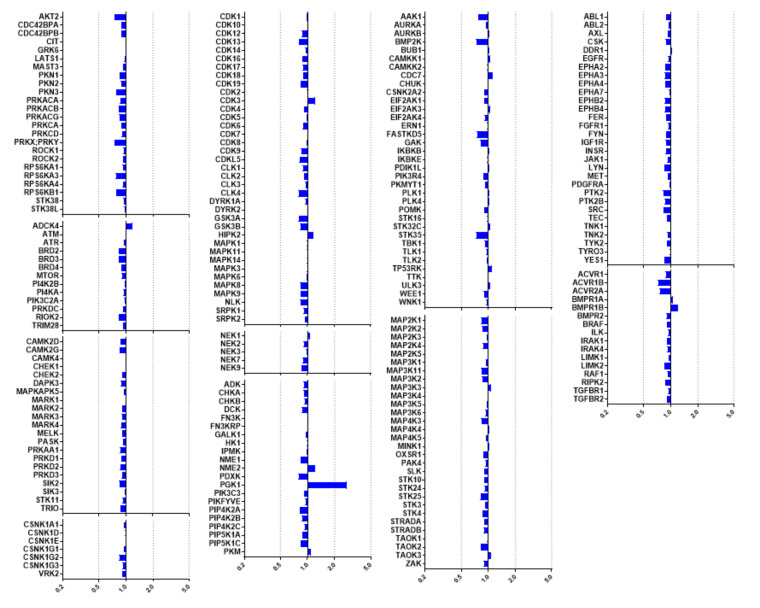
MIBS kinome profiling for thiadiazinone **26** (1 μM)/DMSO.

We then assessed the kinome profile of thiadiazinones **16**, **17**, and **26** all at 1 μM using a multiplexed kinase inhibitor bead set and quantitative mass spectrometry for detection of kinase peptides (MIB-MS) [36,45,46]. The MIB-MS proteomics was able to detect between 350 and 400 kinases in the cell lysates and competitive displacement of specific kinases from the beads was used to determine the kinome profile of the thiadiazinone kinase inhibitors. Compound **16** showed a narrow spectrum kinome profile with weak interactions on MAP4K5, MAP4K3, PRKCD, and PKN1 just below the threshold (Figure 3). Compound **17** also had a narrow spectrum kinome profile, with AKT2, MAP2K4, MAP2K1, and STK35 being just below the threshold (Figure 4). Compound **26** had a narrow spectrum kinome profile but was a weak inhibitor, showing affinity for ACVR1B, ACVR2A, and STK35 (Figure 5).

### 2.4. Modelling of Hits from Kinome Profiling on Thiadiazinones ***16***, ***17***, and ***26***

**Figure 6:**
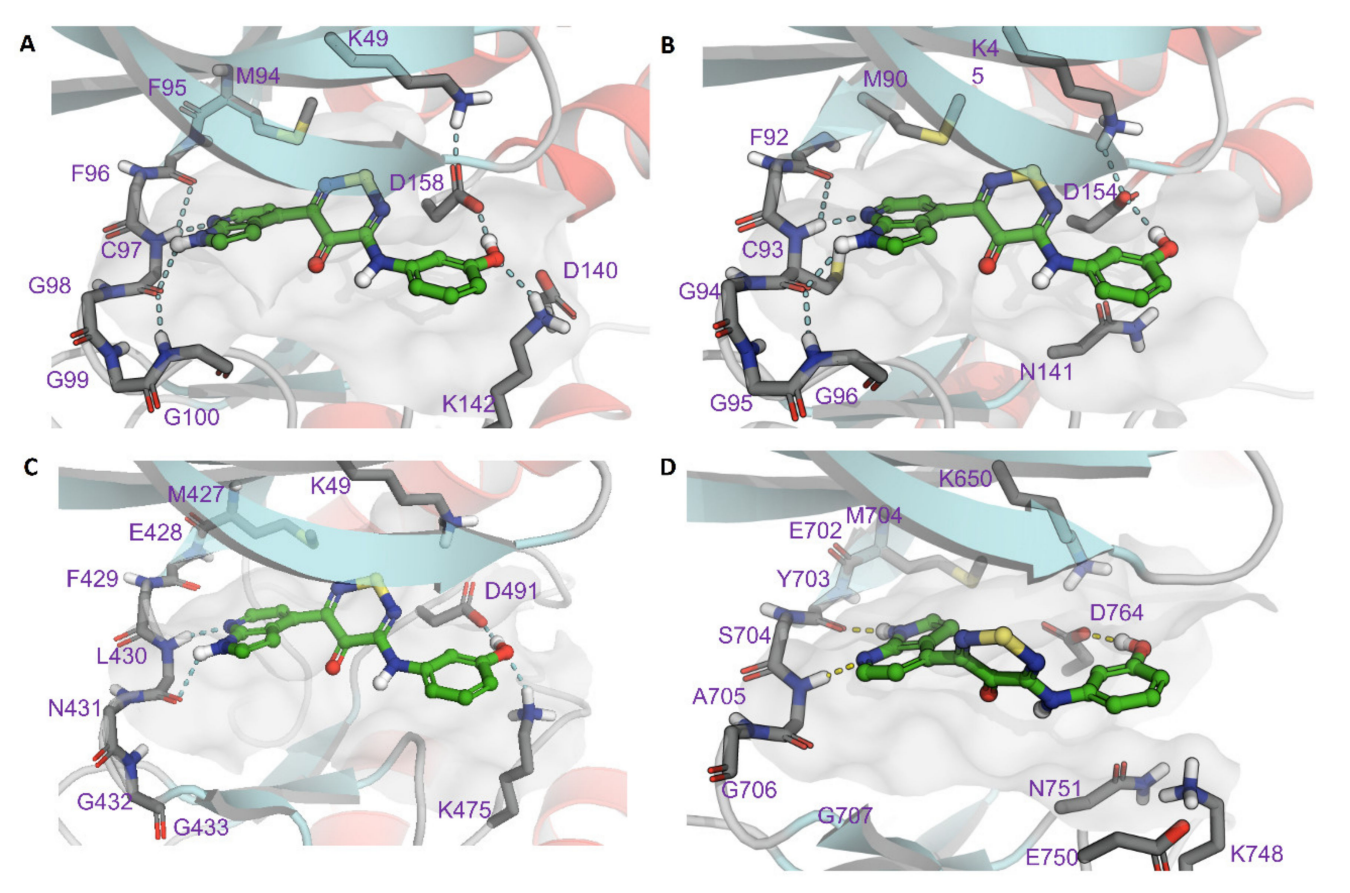
Molecular modeling of **16** on MIBS kinome profiling hits: (**A**) MAP4K5; (**B**) MAP4K3; (**C**) PRKCD; and (**D**) PKN1.

**Figure 7:**
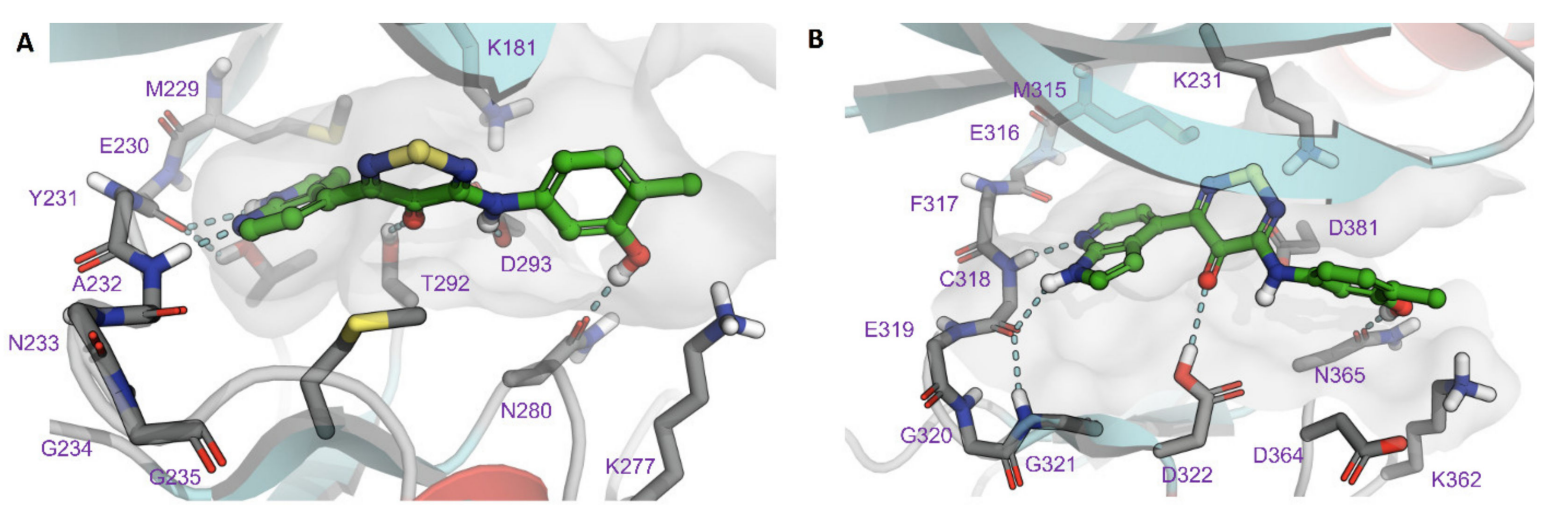
Molecular modeling of thiadiazinone **17** on MIBS kinome profiling hits: (**A**) AKT2 and (**B**) STK35.

**Figure 8:**
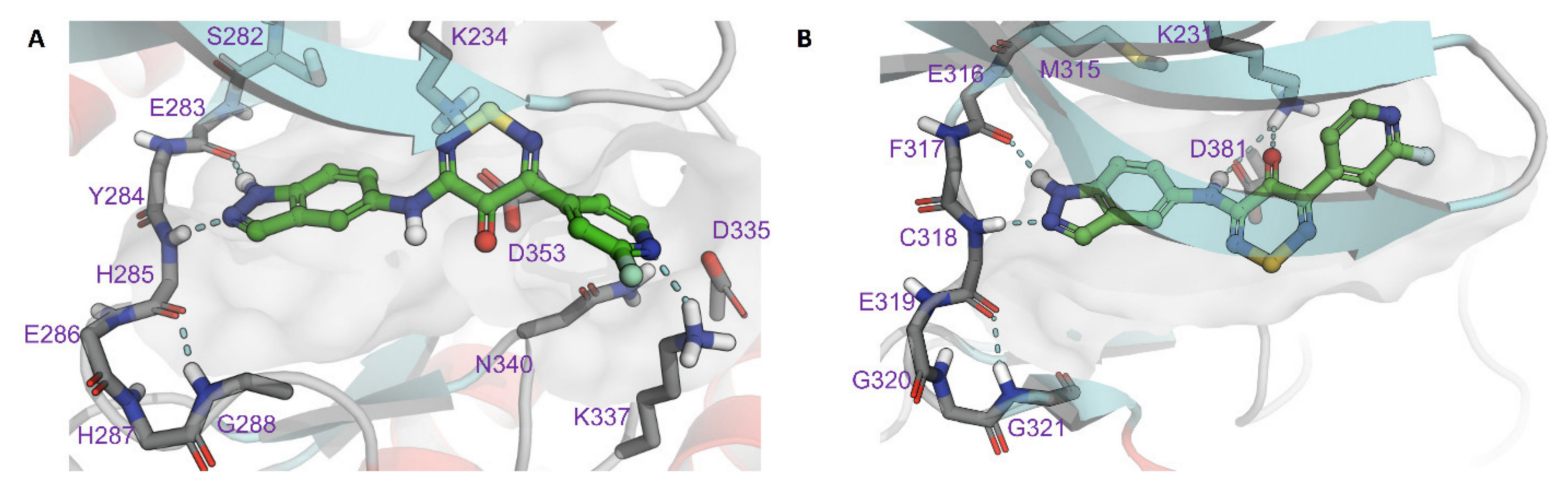
Molecular modeling of thiadiazinone **26** on MIBS kinome profiling hits: (**A**) ACVR1B and (**B**) STK35.

The key hits on thiadiazinones **16**, **17**, and **26** from the MIBS profiling, were then modelled to understand their interactions in the respective ATP binding site (Figure 6, Figure 7 and Figure 8). First, compound **16** was analyzed against MAP4K5 [47], MAP4K3 [48], PRKCD [49], and PKN1 [50]. Docking of the compounds was performed using Schrödinger Maestro [51]. Before docking into the ATP-binding site, prepared from the PDB structure as needed, the compounds were minimized using LigPrep. The docking pose that placed the azo-indole in a position to make a hydrogen bond from the backbone NH of the hinge region of the respective kinases was the most favorable (Figure 6A–D). The 1,2,6-thiadiazinone is relatively passive, acting as a linker to the solvent region where the phenolic alcohol can form a series of different hydrogen bonding interactions. In the case of MAP4K5, MAP4K3, and PRKCD (Figure 6A–C), this is both accepting and donating, forming a rigid fixture at the mouth of the ATP binding site. PKN1 is not able to accommodate this accepting interaction, so there is only a much weaker hydrogen bonding interaction with D764 and no interaction with K748.

In the case of compound **17** (Figure 7), the azo-indole was similarly positioned at the hinge in both AKT2 [52] and STK35 [47,53]. However, the rest of the molecule was not orientated differently in both cases. This enabled additional interactions in both cases where the 1,2,6-thiadiazinone is actively involved, forming a hydrogen bond with the residue T292 in AKT2 and D323 in STK35. This is in addition to a hydrogen bond interaction by the phenolic alcohol with N280 and N365 in AKT2 and STK35, respectively.

Thiadiazinone **26** (Figure 8), similarly to compounds **16** and **17**, formed a strong hydrogen bond in the hinge region of ACVR1B [54] and STK35 [53]. In AVCR1B, the 1,2,6-thiadiazinone was passive with no strong interactions. The pyridine nitrogen did, however, form a hydrogen bond with a solvent-exposed lysine residue K337. In the case of STK35, the 1,2,6-thiadiazinone was actively involved, forming an interaction with a solvent-exposed lysine residue K231. This was in addition to another hydrogen bond interaction with the bringing amine between the indazole and 1,2,6-thiadiazinone. The ATP was too shallow to accommodate the pyridine interaction as seen in AVCR1B and interactions involved with the phenolic alcohol of compound **17**.

## 3. Discussion

Previously, we demonstrated that the 4*H*-1,2,6-thiadiazin-4-one chemotype can function as an ATP-competitive kinase inhibitor, acting as a novel hinge binding motif. This was also the first report of a protein co-crystallization with this rare heterocycle [15]. We have now expanded the repertoire of the 4*H*-1,2,6-thiadiazin-4-one as a spacer unit within a kinase inhibitor affording unique chemical properties. The electronics of the 4*H*-1,2,6-thiadiazin-4-one core allows for negative charge transfer from the sulfur atom towards the conjugated system [55]. This electronic property, exploited in solar cell applications, can partly explain the general lack of kinome promiscuity compared to the dianilinopyrimidine [15]. The modular synthesis and relative narrow kinome spectrum make the 4*H*-1,2,6-thiadiazin-4-one an attractive chemotype for further development.

The chemical tractability of the protein kinases makes them an attractive target for drug development. Over 90 drugs have been approved that target the ATP binding site, predominantly in the field of oncology [19,56]. However, with emerging indications beyond cancer including the treatment of chronic diseases, such as inflammation and neurodegeneration, along with the emergence of resistance, development of compounds with improved potency and selectivity profiles are essential to remain at the cutting edge [57,58]. A number of indirect assays exist to assess the inhibitor affinity, and binding assays are some of the most accurate and robust methods to measure the potency and selectivity of ATP-competitive kinase inhibitors [59,60,61,62,63]. Ligand binding displacement assays are particularly important to provide an accepted form of direct measurement of kinase inhibition in drug optimization of ATP-binding site inhibitors for neglected kinases, where there are currently no robust and validated enzyme activity assays [59,63].

Our results afford new data on a series of interesting starting points for further optimization towards new kinases targets including MAP4K3, MAP4K5, PRKCD, PKN1, AKT2, STK35, and ACVR1B, all of which have some indication in proliferation. The early kinome data, combined with modelling and the accompanying phenotype on bladder cancer, afford exciting prospects for this scaffold. This work firmly puts 1,2,6-thiadiazin-4-one into the medicinal chemistry toolbox and provides another example of the application of sulfur heterocycles in drug design.

## 4. Materials and Methods

### 4.1. Cancer Cell Line Screening Panel

U-CH1 and U-CH2 cell lines were cultured in 80:20 IMDM/RPMI-1640 medium supplemented with 10% fetal bovine serum (FBS), 1% penicillin/streptomycin in gel-coated flasks. A-431, PANC-1, and WS1 cell lines were cultured in DMEM medium supplemented with 10% FBS and 1% penicillin/streptomycin. DU145 and MCF-7 cells were cultured in MEM medium supplemented with 10% FBS and 1% penicillin/streptomycin. 5637 cells were cultured in RPMI-1640 medium supplemented with 10% FBS and 1% penicillin/streptomycin. Cells were seeded in 384-well plates and were treated with test compound in quadruplicate 24 h after plating. Cell viability was assessed at 72 h using alamarBlue (ThermoFisher, Waltham, MA, USA). Fluorescence was measured using Tecan Infinite 200 PRO (Tecan, Männedorf, Switzerland) plate reader with an excitation at 535 nM and emission at 590 nM. IC_50_ values were determined by nonlinear regression using GraphPad PrismTM software (GraphPad Software Inc., San Diego, CA, US). (Positive controls: MCF7, lapatinib IC_50_ = 6.3 μM [64]; 5637, pazopanib IC_50_ = 15 μM [65]; PANC1, panobinostat IC_50_ = 1.0 μM [66]; DU145, docetaxel IC_50_ = 5 nM [67]; UCH1, gefitinib IC_50_ = 1.4 μM [36]; UCH2, gefitinib IC_50_ = 23 μM [36]; A431, lapatinib IC_50_ = 100 nM [68]; WS-1, lapatinib IC_50_ = 13 μM [36]).

### 4.2. MIBS Profiling Methods

Previously described [45]. Briefly, multiplexed inhibitor beads (MIBS) affinity chromatography/MS analysis: SUM159 cells were cultured in a 50:50 mixture of DMEM and Nutrient Mixture F-12 medium (Gibco) supplemented with 5% fetal bovine serum, 1% anti/anti, 5 mg mL^−1^ insulin, and 1 mg·mL^−1^ hydrocortisone. Cells were maintained at 37 °C in a humidified 5% CO_2_ atmosphere. SUM159 cells were grown to 80% confluency, washed twice with PBS, and harvested by scraping cells in lysis buffer [50 mM HEPES, pH 7.5, 150 mM NaCl, 0.5% Triton X-100, 1 mM EDTA, 1 mM EGTA, 10 mM NaF, 2.5 mM Na_3_VO_4_, complete protease inhibitor cocktail (Roche, Basel, Switzerland), phosphatase inhibitor cocktail 2, and 3 (Sigma, St. Louis, MO, USA)]. Lysates were sonicated then clarified by centrifugation at 14000 V g for 15 min at 4 °C. Lysate was then filtered through a 0.2 mm syringe filter and frozen at −80 °C until use. Protein concentration was quantified using a Bradford assay the day of the experiment. DMSO or the indicated concentration of 16, 17, and 26 were added to lysate containing 4 mg of total protein. Lysates were vortexed briefly then incubated for 30 min on ice. Kinases were affinity purified as previously described [46]. Briefly, lysates were diluted to 1.33 mg mL^−1^ with lysis buffer then NaCl concentration brought to 1 M.

Diluted lysates were passed over a mixture of 25 mL of settled beads of each of the following inhibitors conjugated to ECH Sepharose beads: Purvalanol B, PP58, VI-16832, UNC21474A, UNC8088A, and 37.5 mL of settled beads conjugated to CTx-0294885 and VI-16832 [69]. The kinase inhibitor–bead conjugates were previously equilibrated in high-salt buffer (50 mM HEPES pH 7.5, 1M NaCl, 0.5% Triton X-100, 1 mM EDTA, and 1 mM EGTA). MIBs columns were sequentially washed with high-salt buffer, low-salt buffer (50 mm HEPES pH 7.5, 150 mM NaCl, 0.5% Triton X-100, 1 mM EDTA, and 1 mM EGTA), and SDS buffer (50 mm HEPES pH 7.5, 150 mm NaCl, 0.5% Triton X-100, 1 mm EDTA, 1 mm EGTA, and 0.1% SDS). Proteins were eluted by boiling samples in elution buffer (100 mM Tris·HCl pH 6.8, 0.5% SDS, and 1% *β*-mercaptoethanol) for 15 min twice. Dithiothreitol (DTT) was added to a final concentration of 5 mm and samples were incubated at 60 °C for 25 min. Samples were then cooled to room temperature on ice and alkylated by adding iodoacetamide to a final concentration of 20 mm for 30 min in the dark at room temperature. Samples were then concentrated in 10K Amicon Ultra centrifugal concentrators (Millipore, Burlington, MA, USA) followed by methanol and chloroform precipitation of proteins. The final protein pellets were re-suspended in 50 mM HEPES pH 8.0 and incubated with trypsin at 37 °C overnight. Residual detergent was removed by three sequential ethyl acetate extractions then desalted using Pierce C_18_ spin columns (Thermo Scientific, Waltham, MA, USA) according to the manufacturer’s protocol.

### 4.3. Molecular Modelling Methods

#### 4.3.1. Ligand Preparation

Structures of small molecules were parametrized and minimized using the LigPrep module of Schrodinger 2020-4 suite employing OPLS3e force field [51].

#### 4.3.2. Protein Preparation

The kinase a crystal structures co-crystallized with substrate analogues or inhibitors were downloaded from RCSB-databank: MAP4K3-(PDB:5J5T) [70]; PKN1-(PDB:4OTI) [71]; AKT2-(PDB:2X39) [72]. The structures were H-bond optimized and minimized using the standard protein preparation procedure of Schrödinger suite.

#### 4.3.3. Development of Homology-Based Model

Three-dimensional models of ACVR1B, STK35, and MAP4K5 were developed using Biovia Discovery Studio 2019 program (Dassault Systèmes, San Diego, CA, USA). The human forms of target sequences were downloaded from uniport, and psi-blast searched against the PDB-template database. Pre-aligned template structures (ACVR1B-(PDB:5E8X) [73], STK35-(PDB:3MA6), and MAP4K5-(PDB:5J5T) [70]) were downloaded to Discovery Studio and Alignments to corresponding kinase domains. The sequence of corresponding unknown kinase was then aligned to template structures. The models were built using standard settings of the modeler: optimization level = high, Number of models = 20, Refine loops = false, original ligands were copied from templates. Models were then ranked according to the PDF score and highest ranked models were uploaded to Maestro for protein preparation protocol. Models of AVCR1B and MAP4K5 were also successfully generated using protein structure prediction functionality available at GalaxyWEB-modelling site (http://galaxy.seoklab.org/ Accessed on: 6 September 2021), and cross checked with in-house generated models. During the final stage of this study, several AlphaFold structures available including ACVR1B, STK35, and MAPK5 were downloaded [74], prepared, and aligned to compare the positions of key residues with previously generated in-house models, which were consistent with our findings (data not shown).

#### 4.3.4. Molecular Docking

The ligand docking was performed using an induced fit protocol using the standard settings of Schrodinger suite 2020-4 (up to 20 models were generated, dockings were done using SP-level of IFD-setting during Glide docking and redocking steps). The centers of glide grids were set to the centroid of co-crystallized ligands or template-derived ligands. The highest ranked docking poses were visually examined to conclude favorable hinge binding forms.

### 4.4. Chemistry Experimental Section

#### 4.4.1. General Methods and Materials

All chemicals were commercially available except those whose synthesis is described. Anhydrous Na_2_SO_4_ was used for drying organic extracts and all volatiles were removed under reduced pressure. 1,4-Dioxane was dried by refluxing over CaH_2_. All reaction mixtures and column eluents were monitored by TLC using commercial glass-backed thin layer chromatography (TLC) plates (Merck Kieselgel 60 F_254_, Darmstadt, Germany) [75]. The plates were observed under UV light at 254 and 365 nm. The technique of dry flash chromatography was used throughout for all non-TLC scale chromatographic separations using Merck Silica Gel 60 (less than 0.063 mm). Melting points were determined using a PolyTherm-A, Wagner & Munz, Koefler-Hotstage Microscope apparatus (Wagner & Munz, Munich, Germany). Solvents used for recrystallization are indicated after the melting point. UV spectra were obtained using a Perkin-Elmer Lambda-25 UV/vis spectrophotometer (Perkin-Elmer, Waltham, MA, USA) and inflections are identified by the abbreviation “inf”. IR spectra were recorded on a Shimadzu FTIR-NIR Prestige-21 spectrometer (Shimadzu, Kyoto, Japan) with Pike Miracle Ge ATR accessory (Pike Miracle, Madison, WI, USA) and strong, medium, and weak peaks are represented by s, m, and w, respectively. ^1^H and ^13^C NMR spectra were recorded on a Bruker Avance 300 (at 300 and 75 MHz, respectively), or a 500 machine (at 500 and 125 MHz, respectively, Bruker, Billerica, MA, USA, ^1^H and ^13^C NMR spectra of new compounds could be found from Supplementary Materials). Deuterated solvents were used for homonuclear lock and the signals are referenced to the deuterated solvent peaks. Attached proton test (APT) NMR studies were used for the assignment of the ^13^C peaks as *C*H_3_, *C*H_2_, *C*H, and *C*q (quaternary). The Matrix-Assisted Laser Desorption/Ionization-Time of Flight (MALDI-TOF) mass spectra (+ve mode) were recorded on a Bruker Autoflex III Smartbeam instrument (Bruker, Leipzig, Germany), while ESI-APCI+ mass spectra were recorded on a Model 6110 Quadrupole MSD, Agilent Technologies (Agilent Technologies, Santa Clara, CA, USA). High resolution mass spectra were recorded in a ThermoFisher Q Exactive HF-X (ThermoFisher, Bremen, Germany) mass spectrometer coupled with a Waters Acquity H-class liquid chromatograph system. 3,5-Dichloro-4*H*-1,2,6-thiadiazin-4-one (2) [18], 3-chloro-5-[(3-hydroxy-4-methylphenyl)amino]-4*H*-1,2,6-thiadiazin-4-one (9) [15] and 3-chloro-5-morpholino-4*H*-1,2,6-thiadiazin-4-one (11) [29] were prepared according to the reported procedures.

#### 4.4.2. Preparation of 3-Aminosubstituted-4*H*-1,2,6-thiadiazines

*3-[(3-Acetylphenyl)amino]-5-chloro-4*H*-1,2,6-thiadiazin-4-one* (**6**) (General procedure). To a stirred solution of 3,5-dichloro-4*H*-1,2,6-thiadiazin-4-one (2) (366 mg, 2 mmol) in EtOH (4 mL), at ca. 20 °C was added 1-(3-aminophenyl)ethan-1-one (135 mg, 1 mmol) in one portion and the mixture stirred for 30 min at this temperature. Then, 2,6-lutidine (233 μL, 4 mmol) was added and the mixture was stirred at this temperature until complete consumption of the starting material (TLC, 2 h). The yellow solid formed was then filtered under vacuum and washed with EtOH (2 mL), *t*-BuOMe (5 mL), and n-hexane (5 mL) to give the *title compound* **6** (190 mg, 67%) as yellow needles, mp 201–202 °C (from EtOH/THF); R*_f_* 0.41 (DCM); (found: C, 46.70; H, 2.97; N, 14.73. C_11_H_8_ClN_3_O_2_S requires C, 46.90; H, 2.86; N, 14.92%); *λ*_max_(DCM)/nm 295 (log ε 4.12), 321 (4.25), 334 inf (4.18), 413 (3.69); *v*_max_/cm^−1^ 3258m (N-H), 1680s, 1632s, 1589m, 1547s, 1518m, 1487w, 1439m, 1431s, 1356m, 1315w, 1312w, 1287m, 1260m, 1240m, 1177m, 1169m, 997m, 908s, 868m, 810m, 795m; *δ*_H_(300 MHz; DMSO-*d*_6_) 10.32 (1H, s, NH), 8.39 (1H, dd, *J* 1.8, 1.8, Ar H), 8.04 (1H, ddd, *J* 8.1, 2.2, 1.0, Ar H), 7.72 (1H, ddd, *J* 7.7, 1.3, 1.1, Ar H), 7.51 (1H, dd, *J* 8.0, 8.0, Ar H), 2.58 (3H, s, CH_3_); *δ*_C_(75 MHz; DMSO-*d*_6_) 197.5 (*C*q), 157.1 (*C*q), 150.2 (*C*q), 141.4 (*C*q), 138.4 (*C*q), 137.2 (*C*q), 129.0 (*C*H), 125.1 (*C*H), 123.9 (*C*H), 120.2 (*C*H), 26.7 (*C*H_3_); *m*/*z* (MALDI-TOF) 284 (MH^+^+2, 39%), 282 (MH^+^, 100), 265 (37), 240 (66).

*3-Chloro-5-[(3-methoxyphenyl)amino]-4*H*-1,2,6-thiadiazin-4-one* (**7**). Similar treatment of 3,5-dichloro-4*H*-1,2,6-thiadiazin-4-one (**2**) (183 mg, 1 mmol) in EtOH (2 mL), with 3-methoxyaniline (246 mg, 2 mmol) and 2,6-lutidine (116 μL, 2 mmol) for 1 h gave the *title compound* **7** (494 mg, 92%) as yellow plates, mp 176–177 °C (from PhH/EtOH); R*_f_* 0.54 (DCM); (found: C, 44.45; H, 2.97; N, 15.56. C_10_H_8_ClN_3_O_2_S requires C, 44.53; H, 2.99; N, 15.58%); *λ*_max_(DCM)/nm 324 (log *ε* 4.61), 419 (4.00); *v*_max_/cm^−1^ 3306m (N-H), 3067w (C-H ar), 2833 (C-H alip.), 1624s, 1613m, 1609m, 1589m, 1566s, 1514w, 1501m, 1489m, 1483m, 1445w, 1435m, 1427m, 1321m, 1294m, 1275m, 1215m, 1206m, 1188w, 1175w, 1148s, 1059s, 1011w, 932m, 874m, 870m, 831s, 772m, 733m; *δ*_H_(500 MHz; CDCl_3_) 8.66 (1H, br. s, N*H*), 7.31 (1H, dd, *J* 2.2, 2.2, Ar *H*), 7.29 (1H, d, *J* 8.2, Ar *H*), 7.14 (1H, dd, *J* 8.0, 1.8, Ar *H*), 6.74 (1H, dd, *J* 8.3, 2.4, Ar *H*), 3.84 (3H, s, C*H*_3_); *δ*_C_(125 MHz; CDCl_3_) 160.3 (*C*q), 156.6 (*C*q), 149.7 (*C*q), 142.0 (*C*q), 137.9 (*C*q), 130.0 (*C*H), 112.2 (*C*H), 110.3 (*C*H), 105.9 (*C*H), 55.4 (*C*H_3_); *m*/*z* (MALDI-TOF) 272 (MH^+^+2, 39), 270 (MH^+^, 79%), 234 (100), 180 (92).

*3-Chloro-5-[(3-hydroxyphenyl)amino]-4*H*-1,2,6-thiadiazin-4-one* (**8**). Similar treatment of 3,5-dichloro-4*H*-1,2,6-thiadiazin-4-one (**2**) (183 mg, 1 mmol) in EtOH (2 mL), with 2-aminophenol (109 mg, 1 mmol), and 2,6-lutidine (116 μL, 2 mmol) for 30 min gave the *title compound* **8** (228 mg, 89%) as yellow needles, mp 204–205 °C (from PhH/THF); R*_f_* 0.25 (DCM); (found: C, 42.11; H, 2.46; N, 16.24. C_9_H_6_ClN_3_O_2_S requires C, 42.28; H, 2.37; N, 16.44%); *λ*_max_(DCM)/nm 259 (log *ε* 3.78), 288 inf (4.40), 323 (4.23), 417 (4.03); *v*_max_/cm^−1^ 3379br (O-H), 3256 (N-H), 1640m, 1599s, 1560m, 1551s, 1524m, 1466m, 1451m, 1439m, 1321w, 1304m, 1277m, 1227w, 1148m, 1092w, 972m, 870m, 860m, 841m, 783m, 739m, 731m; *δ*_H_(300 MHz; DMSO-*d*_6_) 9.96 (1H, s), 9.52 (1H, br. s), 7.28 (1H, dd, *J* 2,1, 2.1, Ar *H*), 7.17 (1H, dd, *J* 7.7, 1.3, Ar *H*), 7.12 (1H, dd, *J* 8.0, 8.0, Ar *H*), 6.53 (1H, ddd, *J* 7.7, 2.2, 1.3, Ar *H*); *δ*_C_(125 MHz; DMSO-*d*_6_) 157.4 (*C*q), 157.1 (*C*q), 150.0 (*C*q), 141.0 (*C*q), 138.8 (*C*q), 129.2 (*C*H), 111.7 (*C*H), 111.2 (*C*H), 107.7 (*C*H); *m*/*z* (MALDI-TOF) 258 (MH^+^+ 2, 32), 256 (MH^+^, 100%), 238 (56), 220 (45), 166 (32).

*3-Chloro-5-[(5-hydroxy-2-methylphenyl)amino]-4*H*-1,2,6-thiadiazin-4-one* (**10**). Similar treatment of 3,5-dichloro-4*H*-1,2,6-thiadiazin-4-one (**2**) (183 mg, 1 mmol) in EtOH (2 mL), with 3-amino-4-methylphenol (123 mg, 1 mmol) and 2,6-lutidine (116 μL, 2 mmol) for 1 h gave the *title compound* **10** (262 mg, 97%) as yellow needles, mp 191–192 °C (from PhH/*c*-hexane); R*_f_* 0.18 (DCM); (found: C, 44.59; H, 2.91; N, 15.66. C_10_H_8_ClN_3_O_2_S requires C, 44.53; H, 2.99; N, 15.58%); *λ*_max_(DCM)/nm 319 (log *ε* 4.27), 398 (3.73); *v*_max_/cm^−1^ 3420br (O-H), 3364 (N-H), 1620m, 1609m, 1551s, 1518m, 1514m, 1466m, 1462m, 1445m, 1356m, 1267m, 1217m, 1153m, 1105m, 1015m, 1005m, 974m, 864m, 847m, 810m, 737m; *δ*_H_(500 MHz; DMSO-*d*_6_) 9.52 (1H, s), 9.33 (1H, s), 7.04 (1H, d, *J* 8.3, Ar *H*), 6.90 (1H, d, *J* 2.5, Ar *H*), 6.58 (1H, dd, *J* 8.2, 2.5, Ar *H*), 2.09 (3H, s, C*H*_3_); *δ*_C_(125 MHz; DMSO-*d*_6_) 157.0 (*C*q), 155.5 (*C*q), 151.4 (*C*q), 140.4 (*C*q), 135.9 (*C*q), 130.9 (*C*H), 122.6 (*C*q), 113.1 (*C*H), 111.9 (*C*H), 16.6 (C*H*_3_); *m*/*z* (MALDI-TOF) 272 (MH^+^+2, 70%), 270 (MH^+^, 100), 252 (96), 234 (55), 211 (34), 180 (37).

*3-Chloro-5-(4-methylpiperazin-1-yl)-4*H*-1,2,6-thiadiazin-4-one* (**12**). Similar treatment of 3,5-dichloro-4*H*-1,2,6-thiadiazin-4-one (**2**) (183 mg, 1 mmol) in EtOH (2 mL), with *N*-methylpiperazine (215 μL, 2 mmol) for 30 min gave the *title compound* **12** (245 mg, 99%) as yellow needles, mp 76–77 °C (from *c*-hexane); R*_f_* 0.29 (DCM/*t-*BuOMe, 50:50); (found: C, 38.72; H, 4.66; N, 22.53. C_8_H_11_ClN_4_OS requires C, 38.95; H, 4.49; N, 22.71%); *λ*_max_(DCM)/nm 269 (log *ε* 3.78), 312 (4.15), 320 inf (4.18), 406 (3.78); *v*_max_/cm^−1^ 2965w, 2934w, 2845w and 2804w (C-H), 1624s, 1493m, 1445m, 1433m, 1402m, 1356m, 1335w, 1306m, 1288m, 1271m, 1196m, 1159m, 1144m, 1080m, 1059m, 1005m, 984m, 939m, 864m, 854m, 843m, 791m, 775m, 760m, 727m; *δ*_H_(500 MHz; CDCl_3_) 3.92 (4H, br. s, C*H*_2_), 2.51 (4H, t, *J* 5.1, C*H*_2_), 2.32 (3H, s, C*H*_3_); *δ*_C_(125 MHz; CDCl_3_) 158.7 (*C*q), 152.9 (*C*q), 145.2 (*C*q), 54.8 (*C*H_2_), 46.6 (*C*H_2_), 45.9 (*C*H_3_); *m*/*z* (ESI+) 249 (MH^+^+2, 37%), 247 (MH^+^, 100), 130 (82).

*5-Chloro-3-[(1*H*-indazol-5-yl)amino]-4*H*-1,2,6-thiadiazin-4-one* (**13**). Similar treatment of 3,5-dichloro-4*H*-1,2,6-thiadiazin-4-one (**2**) (183 mg, 1 mmol) in EtOH (2 mL), with 1*H*-indazol-5-amine (133 mg, 1 mmol) and 2,6-lutidine (116 μL, 2 mmol) for 1 h gave the *title compound* **13** (255 mg, 91%) as orange plates, mp 290–291 °C (from PhMe/THF); R*_f_* 0.53 (DCM/*t-*BuOMe, 80:20); (found: C, 42.82; H, 2.09; N, 24.91. C_10_H_6_ClN_5_OS requires C, 42.94; H, 2.16; N, 25.04%); *λ*_max_(THF)/nm 254 (log *ε* 4.57), 286 (4.59), 325 (4.40), 338 (4.38), 423 (3.77); *v*_max_/cm^−1^ 3393m (N-H), 3213w and 3086w (C-H), 1626m, 1589m, 1576w, 1562m, 1557m, 1508s, 1504m, 1427m, 1281m, 1198m, 1138m, 961w, 937s, 872s, 841w, 808m, 754m; *δ*_H_(500 MHz; DMSO-*d*_6_) 13.06 (1H, s, N*H*), 10.18 (1H, s, N*H*), 8.18 (1H, s, Ar *H*), 8.07 (1H, s, Ar *H*), 7.65 (1H, d, *J* 7.5, Ar *H*), 7.52 (1H, d, *J* 7.5, Ar *H*); *δ*_C_(125 MHz; DMSO-*d*_6_) 157.1 (*C*q), 150.5 (*C*q), 140.5 (*C*q), 137.2 (*C*q), 133.5 (*C*H), 130.6 (*C*q), 122.5 (*C*q), 122.0 (*C*H), 111.7 (*C*H), 110.1 (*C*H); *m*/*z* (MALDI-TOF) 282 (MH^+^+2, 33%), 280 (MH^+^, 100), 244 (44), 190 (50).

#### 4.4.3. Preparation of 5-Amino-Substituted 3-Arylthiadiazinones

*3-[(3-Acetylphenyl)amino]-5-(1*H*-pyrrolo[2,3-*b*]pyridin-4-yl)-4*H*-1,2,6-thiadiazin-4-one* (**14**). Similar treatment of 3-[(3-acetylphenyl)amino]-5-chloro-4*H*-1,2,6-thiadiazin-4-one (**6**) (56.3 mg, 0.20 mmol) with (1*H*-pyrrolo[2,3-*b*]pyridin-4-yl)boronic acid (40.8 mg, 0.22 mmol) for 18 h gave the *title compound* **14** (35.5 mg, 49%) as yellow needles, mp 271–273 °C (from EtOH/PhH); R*_f_* 0.46 (DCM/Et_2_O, 50:50); (found: C, 59.25; H, 3.72; N, 19.06. C_18_H_13_N_5_O_2_S requires C, 59.49; H, 3.61; N, 19.27%); *λ*_max_(THF)/nm 290 (log *ε* 4.00), 360 (4.40), 415 inf (4.18); *v*_max_/cm^−1^ 3148w, 2916w, 1672 m, 1612m, 1583m, 1451s, 1535s, 1483w, 1443m, 1429w, 1323m, 1287w, 1265s, 1223m, 953m, 908m, 898m, 878m, 851m, 843m, 833m, 825m, 816m, 783m, 768m, 739m, 723m, 704m; *δ*_H_(500 MHz; DMSO-*d*_6_) 11.80 (1H, s), 10.39 (1H, s), 8.47 (1H, dd, *J* 1.8, 1.8, Ar *H*), 8.33 (1H, d, *J* 5.1, Ar *H*), 8.12 (1H, dd, *J* 8.0, 1.1, Ar *H*), 7.75 (1H, d, *J* 5.0, Ar *H*), 7.73 (1H, d, *J* 1.1, Ar *H*), 7.57–7.52 (2H, m, Ar *H*), 6.77 (1H, dd, *J* 3.3, 1.9, Ar *H*); *δ*_C_(75 MHz; DMSO-*d*_6_) 159.3 (*C*q), 152.4 (*C*q), 151.9 (*C*q), 149.4 (*C*q), 141.8 (*C*H), 138.4 (*C*q), 137.2 (*C*q), 133.6 (*C*q), 129.0 (*C*H), 126.9 (*C*H), 125.2 (*C*H), 123.7 (*C*H), 120.3 (*C*H), 116.9 (*C*q), 114.8 (*C*H), 100.8 (*C*H), 26.7 (*C*H_3_); *m*/*z* (ESI+) 363 (M^+^, 56%), 362 (M^+^-H, 100), 350 (62); HRMS (ESI+) found for MH^+^ 364.08444, C_18_H_14_N_5_O_2_S^+^ requires 364.08682.

*3-[(3-Methoxyphenyl)amino]-5-(1*H*-pyrrolo[2,3-*b*]pyridin-4-yl)-4*H*-1,2,6-thiadiazin-4-one* (**15**) (General procedure A). To a mixture of 3-chloro-5-[(3-methoxyphenyl)amino]-4*H*-1,2,6-thiadiazin-4-one (**7**) (53.9 mg, 0.20 mmol), (1*H*-pyrrolo[2,3-*b*]pyridin-4-yl)boronic acid (35.7 mg, 0.22 mmol), Pd[3,5-(F_3_C)_2_C_6_H_3_]_3_ (21.2 mg, 5 mol%), DPEPhos (5.3 mg, 5 mol%) and powdered dry K_2_CO_3_ (66.4 mg, 0.48 mmol) was added dioxane (2 mL) and H_2_O (0.3 mL). The stirred suspension was then deaerated by bubbling of Ar through it for 5 min and then heated at reflux under Ar until complete consumption of the starting thiadiazine (TLC, 20 h). The mixture was cooled to ca. 20 °C, then filtered and washed with H_2_O (5 mL) and EtOH (5 mL) and dried under vacuum to give the *title compound* **15** (58.4 mg, 83%) as orange needles, mp 257–258 °C (from *c*-hexane); R*_f_* 0.59 (DCM/*t*-BuOMe, 80:20); (found: C, 58.12; H, 3.67; N, 19.85. C_17_H_13_N_5_O_2_S requires C, 58.11; H, 3.73; N, 19.93%); *λ*_max_(THF)/nm 360 (log *ε* 4.05), 422 inf (3.79); *v*_max_/cm^−1^ 3136w (C-H), 1613m, 1605m, 1587m, 1541m, 1534s, 1531s, 1483m, 1462m, 1327m, 1288m, 1240w, 1223m, 1211m, 1155s, 1092w, 1049m, 966w, 897m, 872w, 792m, 777m, 768m, 760m, 718m; *δ*_H_(500 MHz; DMSO-*d*_6_) 11.79 (1H, br, N*H*), 10.14 (1H, s, N*H*), 8.32 (1H, d, *J* 5.0, Ar *H*), 7.38 (1H, dd, *J* 5.0, Ar *H*), 7.57–7.48 (3H, m, Ar *H*), 7.28 (1H, dd, *J* 8.2, 8.2, Ar *H*), 6.76 (1H, d, *J* 3.0, 1.7, Ar *H*), 6.72 (1H, dd, *J* 8.1, 1.7, Ar *H*), 3.77 (3H, s, C*H*_3_); *δ*_C_(75 MHz; DMSO-*d*_6_) 159.4 (*C*q), 159.3 (*C*q), 152.3 (*C*q), 151.6 (*C*q), 149.4 (*C*q), 141.8 (*C*H), 139.1 (*C*q), 133.7 (*C*q), 129.4 (*C*H), 129.9 (*C*H), 116.9 (*C*q), 114.8 (*C*H), 112.9 (*C*H), 109.3 (*C*H), 106.6 (*C*H), 100.9 (*C*H), 55.0 (*C*H_3_); *m*/*z* (MALDI-TOF) 352 (MH^+^, 100%), 351 (M^+^, 35); HRMS (ESI+) found for MH^+^ 352.08567, C_17_H_14_N_5_O_2_S^+^ requires 352.08682.

*3-[(3-Hydroxyphenyl)amino]-5-(1*H*-pyrrolo[2,3-*b*]pyridin-4-yl)-4*H*-1,2,6-thiadiazin-4-one (**16**).* Similar treatment of 3-chloro-5-[(3-hydroxy-4-methylphenyl)amino]-4*H*-1,2,6-thiadiazin-4-one (**8**) (53.9 mg, 0.20 mmol) with (1*H*-pyrrolo[2,3-*b*]pyridin-4-yl)boronic acid (40.3 mg, 0.22 mmol) for 24 h gave after chromatography (DCM/*t*-BuOMe, 50:50) the *title compound* **16** (24.2 mg, 36%) as yellow plates, mp 326–327 °C (from PhH); R*_f_* 0.56 (DCM/*t*-BuOMe, 50:50); (found: C, 57.14; H, 3.22; N, 20.52. C_16_H_11_N_5_O_2_S requires C, 56.97; H, 3.29; N, 20.76%); *λ*_max_(THF)/nm 366 (log *ε* 4.33), 423 inf (4.07); *v*_max_/cm^−1^ 3401w and 3331w (N-H), 1612m, 1591s, 1587s, 1543s, 1503m, 1381w, 1332m, 1269w, 1204m, 1167m, 1153m, 989m, 793m, 789m, 770m, 727m, 716m; *δ*_H_(500 MHz; DMSO-*d*_6_) 11.79 (1H, s), 10.03 (1H, s), 9.48 (1H, s), 8.31 (1H, d, *J* 5.0, Ar *H*), 7.74 (1H, d, *J* 5.0, Ar *H*), 7.55 (1H, dd, *J* 2.9, 2.9, Ar *H*), 7.39 (1H, dd, *J* 1.9, 1.9, Ar *H*), 7.25 (1H, d, *J* 8.0, Ar *H*), 7.15 (1H, dd, *J* 8.0, 8.0, Ar *H*), 6.76 (1H, dd, *J* 3.2, 1.9, Ar *H*), 6.55 (1H, dd, *J* 8.0, 1.7, Ar *H*); *δ*_C_(75 MHz; DMSO-*d*_6_) 159.3 (*C*q), 157.5 (*C*q), 152.2 (*C*q), 151.5 (*C*q), 149.4 (*C*q), 141.8 (*C*H), 138.9 (*C*q), 133.7 (*C*q), 129.2 (*C*H), 126.9 (*C*H), 116.9 (*C*q), 114.8 (*C*H), 111.7 (*C*H), 107.7 (*C*H), 100.9 (*C*H); *m*/*z* (MALDI-TOF) 338 (MH^+^, 100%), 337 (M^+^, 67); HRMS (ESI+) found for MH^+^ 338.06991, C_16_H_12_N_5_O_2_S^+^ requires 338.07117.

*3-[(3-Hydroxy-4-methylphenyl)amino]-5-(1*H*-pyrrolo[2,3-*b*]pyridin-4-yl)-4*H*-1,2,6-thiadiazin-4-one* (**17**). Similar treatment of 3-chloro-5-[(3-hydroxy-4-methylphenyl)amino]-4*H*-1,2,6-thiadiazin-4-one (**9**) (53.9 mg, 0.20 mmol) with (1*H*-pyrrolo[2,3-*b*]pyridin-4-yl)boronic acid (30.0 mg, 0.22 mmol) for 2 h gave after chromatography (DCM/Et_2_O/THF, 50:40:10) the *title compound* **17** (51.4 mg, 73%) as yellow needles, mp 292–293 °C (from PhH); R*_f_* 0.61 (DCM/Et_2_O/THF, 50:40:10); (found: C, 58.12; H, 3.67; N, 19.85. C_17_H_13_N_5_O_2_S requires C, 58.11; H, 3.73; N, 19.93%); *λ*_max_(THF)/nm 295 (log *ε* 3.75), 366 (4.00), 430 inf (3.68); *v*_max_/cm^−1^ 3298w, 3138w, 2882w, 1614m, 1597m, 1591m, 1535s, 1503m, 1422m, 1277w, 1260w, 1231w, 1219w, 1207w, 1186w, 1159w, 1155w, 1128w, 1111m, 995m, 899m, 856m, 789m, 729m, 718s; *δ*_H_(300 MHz; DMSO-*d*_6_) 11.79 (1H, br s), 9.98 (1H, s), 9.39 (1H, s), 8.31 (1H, d, *J* 5.0, Ar *H*), 7.74 (1H, d, *J* 5.0, Ar *H*), 7.55 (1H, dd, *J* 2.8, 2.8, Ar *H*), 7.42 (1H, d, *J* 1.9, Ar *H*), 7.13 (1H, dd, *J* 8.2, 1.9, Ar *H*), 7.03 (1H, d, *J* 8.0, Ar *H*), 6.76 (1H, dd, *J* 3.3, 1.9, Ar *H*), 2.10 (3H. s, C*H*_3_); *δ*_C_(75 MHz; DMSO-*d*_6_) 159.3 (*C*q), 155.1 (*C*q), 152.2 (*C*q), 151.2 (*C*q), 149.4 (*C*q), 141.7 (*C*H), 136.3 (*C*q), 133.8 (*C*q), 130.2 (*C*H), 126.8 (*C*H), 119.8 (*C*q), 116.9 (*C*q), 114.8 (*C*H), 111.8 (*C*H), 107.3 (*C*H), 100.9 (*C*H), 15.5 (*C*H_3_); *m*/*z* (MALDI-TOF) 352 (MH^+^, 90%), 351 (M^+^, 100); HRMS (ESI+) found for MH^+^ 352.08558, C_17_H_14_N_5_O_2_S^+^ requires 352.08682.

*3-[(5-Hydroxy-2-methylphenyl)amino]-5-(1*H*-pyrrolo[2,3-*b*]pyridin-4-yl)-4*H*-1,2,6-thiadiazin-4-one* (**18**). Similar treatment of 2-[(5-chloro-4-oxo-4*H*-1,2,6-thiadiazin-3-yl)amino]-*N*-methylbenzamide (**10**) (59.3 mg, 0.20 mmol) with (1*H*-pyrrolo[2,3-*b*]pyridin-4-yl)boronic acid (39.2 mg, 0.22 mmol) for 3 h gave after chromatography (DCM/Et_2_O, 50:50) the *title compound* **18** (39.1 mg, 56%) as yellow needles, mp 304–305 °C (from PhH); R*_f_* 0.63 (DCM/Et_2_O, 50:50); (found: C, 58.36; H, 3.69; N, 19.74. C_17_H_13_N_5_O_2_S requires C, 58.11; H, 3.73; N, 19.93%); *λ*_max_(THF)/nm 292 (log *ε* 3.34), 364 (3.60), 420 inf (3.37); *v*_max_/cm^−1^ 3339w, 3102w, 2880w, 1612m, 1587m, 1541s, 1537s, 1481m, 1477m, 1381w, 1321m, 1287w, 1221w, 1204w, 1155m, 1130w, 1115w, 1007m, 982m, 899m, 866m, 812m, 808m, 797m, 791m, 764m, 731m, 727m, 716m; *δ*_H_(300 MHz; DMSO*-d*_6_) 11.79 (1H, br s), 9.58 (1H, s), 9.34 (1H, s), 8.31 (1H, d, *J* 5.0, Ar *H*), 7.80 (1H, d, *J* 5.1, Ar *H*), 7.56 (1H, dd, *J* 3.0, 3.0, Ar *H*), 7.12 (1H, d, *J* 2.5, Ar *H*), 7.07 (1H, d, *J* 8.3, Ar *H*), 6.78 (1H, dd, *J* 3.4, 1.9, Ar *H*), 6.58 (1H, dd, *J* 8.3, 2.5, Ar *H*); *δ*_C_(75 MHz; DMSO*-d*_6_) 159.2 (*C*q), 155.6 (*C*q), 153.1 (*C*q), 150.7 (*C*q), 149.4 (*C*q), 141.8 (*C*H), 136.0 (*C*q), 133.6 (*C*q), 130.8 (*C*H), 126.9 (*C*H), 121.7 (*C*q), 116.9 (*C*q), 114.9 (*C*H), 112.6 (*C*H), 111.0 (*C*H), 100.8 (*C*H), 16.6 (*C*H_3_); *m*/*z* (MALDI-TOF) 352 (MH^+^, 17%), 351 (M^+^, 100); HRMS (ESI+) found for MH^+^ 352.08576, C_17_H_14_N_5_O_2_S^+^ requires 352.08682.

*3-Morpholino-5-(1*H*-pyrrolo[2,3-*b*]pyridin-4-yl)-4*H*-1,2,6-thiadiazin-4-one* (**19**). Similar treatment of 3-chloro-5-[(3-hydroxy-4-methylphenyl)amino]-4*H*-1,2,6-thiadiazin-4-one (**11**) (53.9 mg, 0.20 mmol) with (1*H*-pyrrolo[2,3-*b*]pyridin-4-yl)boronic acid (30.2 mg, 0.22 mmol) for 22 h gave after chromatography (DCM/*t*-BuOMe, 50:50) the *title compound* **19** (36.7 mg, 58%) as yellow needles, mp 256–257 °C (from EtOH/THF); R*_f_* 0.75 (DCM/*t*-BuOMe, 50:50); (found: C, 53.08; H, 4.12; N, 22.35. C_14_H_13_N_5_O_2_S requires C, 53.22; H, 4.16; N, 22.21%); *λ*_max_(THF)/nm 291 (log *ε* 3.63), 298 (3.62), 358 (3.71), 408 inf (3.55); *v*_max_/cm^−1^ 3140w, 3069w, 2978w, 2887w, 2852w, 1603s, 1560w, 1493m, 1483m, 1439m, 1404m, 1327m, 1319m, 1310m, 1283m, 1269m, 1248s, 1217m, 1192m, 1124s, 1065m, 1007m, 959m, 922m, 903m, 878m, 858m, 835m, 810s, 795s, 746m, 727m, 710m; *δ*_H_(300 MHz; DMSO*-d*_6_) 11.76 (1H, br s, N*H*), 8.27 (1H, d, *J* 5.0, Ar *H*), 7.53 (1H, dd, *J* 3.2, 2.7, Ar *H*), 7.51 (1H, d, *J* 5.0, Ar *H*), 6.60 (1H, dd, *J* 3.4, 1.8, Ar *H*), 3.78 (4H, br. s, C*H*_2_), 3.72 (4H, br. s, C*H*_2_); *δ*_C_(75 MHz; DMSO*-d*_6_) 162.3 (*C*q), 155.0 (*C*q), 154.3 (*C*q), 149.3 (*C*q), 141.7 (*C*H), 134.0 (*C*q), 126.8 (*C*H), 117.0 (*C*q), 114.9 (*C*H), 100.7 (*C*H), 65.7 (*C*H_2_), 46.2 (*C*H_2_); *m*/*z* (MALDI-TOF) 316 (M+H^+^, 100%), 315 (M^+^, 89); HRMS (ESI+) found for MH^+^ 316.08558, C_14_H_14_N_5_O_2_S^+^ requires 316.08682.

*3-(4-Methylpiperazin-1-yl)-5-(1*H*-pyrrolo[2,3-*b*]pyridin-4-yl)-4*H*-1,2,6-thiadiazin-4-one* (**20**). Similar treatment of 3-chloro-5-(4-methylpiperazin-1-yl)-4*H*-1,2,6-thiadiazin-4-one (**12**) (49.4 mg, 0.20 mmol) with (1*H*-pyrrolo[2,3-*b*]pyridin-4-yl)boronic acid (26.0 mg, 0.22 mmol) for 1 h gave after chromatography (DCM/THF, 50:50) the *title compound* **20** (60.8 mg, 93%) as yellow needles, mp 195–196 °C (from *c*-hexane/CHCl_3_); R*_f_* 0.21 (DCM/THF, 50:50); (found: C, 54.59; H, 5.14; N, 25.36. C_15_H_16_N_6_OS requires C, 54.86; H, 4.91; N, 25.59%); *λ*_max_(DCM)/nm 259 (log *ε* 4.08), 358 (4.26), 409 (4.06); *v*_max_/cm^−1^ 3134w, 2938w, 2884w, 2843w, 2787w, 1611m, 1593m, 1564m, 1463m, 1447m, 1410m, 1368w, 1331m, 1281m, 1259m, 1234m, 1219m, 1196m, 1165m, 1148m, 1090m, 1078m, 1001m, 1051m, 895m, 870m, 827s, 789s, 746m, 733m, 708s, 660m, 613m; *δ*_H_(500 MHz; DMSO*-d*_6_) 11.76 (1H, s, N*H*), 8.27 (1H, d, *J* 5.0, Ar *H*), 7.53 (1H, dd, *J* 3.0, 3.0, Ar *H*), 7.51 (1H, d, *J* 5.0, Ar *H*), 6.60 (1H, dd, *J* 3.4, 1.9, Ar *H*), 3.77 (4H, br. s, C*H*_2_), 2.45 (4H, t, *J* 5.0, C*H*_2_), 2.22 (3H, s, C*H*_3_); *δ*_C_(125 MHz; DMSO*-d*_6_) 162.3 (*C*q), 155.0 (*C*q), 154.2 (*C*q), 149.3 (*C*q), 141.8 (*C*H), 134.1 (*C*q), 126.8 (*C*H), 117.0 (*C*q), 114.9 (*C*H), 100.7 (*C*H), 54.2 (*C*H_2_), 45.54 (*C*H_2_), 45.5 (*C*H_3_); *m*/*z* (APCI+) 329 (MH^+^, 100%), 309 (34); HRMS (ESI+) found for MH^+^ 329.11702, C_15_H_17_N_6_OS^+^ requires 329.11845.

*5-(3-Hydroxyphenyl)-3-[(1*H*-indazol-5-yl)amino]-4*H*-1,2,6-thiadiazin-4-one* (**23**) (General procedure B). To a mixture of 5-chloro-3-[(1*H*-indazol-5-yl)amino]-4*H*-1,2,6-thiadiazin-4-one (**13**) (55.9 mg, 0.20 mmol), (3-hydroxyphenyl)boronic acid (55.2 mg, 0.40 mmol), Pd(dppf)Cl_2_ (14.6 mg, 10 mol%) and powdered dry Na_2_CO_3_ (42.4 mg, 0.40 mmol), in a sealed tube, was added dioxane (0.9 mL) and H_2_O (0.1 mL). The stirred suspension was then deaerated by bubbling of Ar through it for 5 min and then heated at ca. 140 °C under Ar until complete consumption of the starting thiadiazine (TLC, 1.5 h). The mixture was cooled to ca. 20 °C, adsorbed onto silica and chromatographed (DCM/*t*-BuOMe, 50:50) to give the *title compound* **23** (46.1 mg, 68%) as orange needles, mp 282–283 °C (from MeOH/THF); R*_f_* 0.52 (DCM/*t*-BuOMe, 50:50); (found: C, 59.79; H, 3.43; N, 20.81. C_16_H_11_N_5_O_2_S requires C, 56.97; H, 3.29; N, 20.76%); *λ*_max_(THF)/nm 326 inf (log *ε* 3.81), 374 (4.00), 391 inf (3.79), 447 (3.49); *v*_max_/cm^−1^ 3331w, 1605m, 1591m, 1584m, 1580m, 1553s, 1510m, 1503m, 1346m, 1300m, 1229m, 1169w, 1086w, 997w, 968w, 951m, 895w, 866m, 829m, 814m, 800m, 814m, 779m, 754m; *δ*_H_(500 MHz; DMSO*-d*_6_) 13.06 (1H, s), 10.15 (1H, s), 9.60 (1H, s), 8.25 (1H, s, Ar *H*), 8.08 (1H, s, Ar *H*), 7.68 (1H, dd, *J* 8.9, 1.4, Ar *H*), 7.56–7.51 (3H, m, Ar *H*), 7.27 (1H, dd, *J* 7.8, 7.8, Ar *H*), 6.86 (1H, dd, *J* 8.3, 2.4, Ar *H*); *δ*_C_(125 MHz; DMSO*-d*_6_) 159.6 (*C*q), 156.4 (*C*q), 152.5 (*C*q), 151.0 (*C*q), 137.2 (*C*q), 136.5 (*C*q), 133.5 (*C*H), 130.9 (*C*q), 128.9 (*C*H), 122.6 (*C*q), 122.1 (*C*H), 119.0 (*C*H), 116.6 (*C*H), 114.9 (*C*H), 111.5 (*C*H), 110.1 (*C*H); *m*/*z* (APCI+) 338 (MH^+^, 100%), 313 (36); HRMS (ESI+) found for MH^+^ 338.06985, C_16_H_12_N_5_O_2_S^+^ requires 338.07117.

*5-(5-Hydroxy-2-methylphenyl)-3-[(1*H*-indazol-5-yl)amino]-4*H*-1,2,6-thiadiazin-4-one* (**24**). Similar treatment of 5-chloro-3-[(1*H*-indazol-5-yl)amino]-4*H*-1,2,6-thiadiazin-4-one (**13**) (55.9 mg, 0.20 mmol) with (5-hydroxy-2-methylphenyl)boronic acid (60.8 mg, 0.40 mmol) for 1.5 h gave after chromatography (DCM/*t*-BuOMe, 75:25) the *title compound* **24** (48.8 mg, 69%) as yellow needles, mp 268–269 °C (from PhH/THF); R*_f_* 0.35 (DCM/*t*-BuOMe, 75:25); (found: C, 58.46; H, 3.51; N, 19.69. C_17_H_13_N_5_O_2_S requires C, 58.11; H, 3.73; N, 19.93%); *λ*_max_(THF)/nm 307 (log *ε* 3.90), 358 (3.93), 426 (3.44); *v*_max_/cm^−1^ 3337w, 3233w, 2689w, 1612m, 1591m, 1576m, 1557s, 1514m, 1504m, 1333m, 1300m, 1267w, 1238w, 1213w, 1167w, 1097w, 966w, 953m, 937w, 883w, 866m, 847m, 802m, 752m; *δ*_H_(500 MHz; DMSO*-d*_6_) 13.06 (1H, s), 10.11 (1H, s), 9.35 (1H, s), 8.26 (1H, d, *J* 1.2, Ar *H*), 8.08 (1H, s, Ar *H*), 7.69 (1H, dd, *J* 9.0, 1.9, Ar *H*), 7.53 (1H, d, *J* 8.9, Ar *H*), 7.07 (1H, d, *J* 8.3, Ar *H*), 6.78 (1H, d, *J* 2.6, Ar *H*), 6.75 (1H, dd, *J* 8.2, 2.6, Ar *H*), 2.10 (3H, s, C*H*_3_); *δ*_C_(125 MHz; DMSO*-d*_6_) 159.0 (*C*q), 155.8 (*C*q), 154.7 (*C*q), 152.1 (*C*q), 137.2 (*C*q), 136.3 (*C*q), 133.5 (*C*H), 130.9 (*C*H), 130.8 (*C*q), 126.1 (*C*q), 122.6 (*C*q), 122.1 (*C*H), 115.9 (*C*H), 113.8 (*C*H), 111.5 (*C*H), 110,1 (*C*H), 18.6 (*C*H_3_); *m*/*z* (APCI+) 352 (MH^+^, 100%), 177 (M^+^, 67); HRMS (ESI+) found for MH^+^ 352.08560, C_17_H_14_N_5_O_2_S^+^ requires 352.08682.

*3-[(1*H*-Indazol-5-yl)amino]-5-(3-methoxyphenyl)-4*H*-1,2,6-thiadiazin-4-one* (**25**). Similar treatment of 5-chloro-3-[(1*H*-indazol-5-yl)amino]-4*H*-1,2,6-thiadiazin-4-one (**13**) (55.9 mg, 0.20 mmol) with (3-methoxyphenyl)boronic acid (60.8 mg, 0.40 mmol) for 2 h gave after chromatography (DCM/*t*-BuOMe, 95:5) the *title compound* **25** (55.1 mg, 78%) as orange needles, mp 220–221 °C (from EtOH/THF); R*_f_* 0.29 (DCM/*t*-BuOMe, 95:5); (found: C, 58.20; H, 3.65; N, 19.89. C_17_H_13_N_5_O_2_S requires C, 58.11; H, 3.73; N, 19.93%); *λ*_max_(DCM)/nm 376 (log *ε* 4.04), 453 (3.53); *v*_max_/cm^−1^ 3335w (N-H), 2943w (C-H), 1605m, 1595m, 1587m, 1547m, 1512m, 1501m, 1491m, 1343m, 1323w, 1287m, 1244m, 1227m, 1215m, 1182w, 1167w, 1038m, 941m, 868m, 829m, 782m, 731m; *δ*_H_(300 MHz; DMSO*-d*_6_) 13.06 (1H, br. s, N*H*), 10.22 (1H, s, N*H*), 8.26 (1H, d, *J* 1.4, Ar *H*), 8.08 (1H, s, Ar *H*), 7.72–7.66 (3H, m, Ar *H*), 7.54 (1H, d, *J* 9.1, Ar *H*), 7.41 (1H, dd, *J* 8.0, 8.0, Ar *H*), 7.05 (1H, ddd, *J* 4.8, 2.5, 0.9, Ar *H*), 3.81 (3H, s, C*H*_3_); *δ*_C_(75 MHz; DMSO*-d*_6_) 159.6 (*C*q), 158.7 (*C*q), 152.5 (*C*q), 150.6 (*C*q), 137.2 (*C*q), 136.5 (*C*q), 133.5 (*C*H), 130.9 (*C*q), 129.1 (*C*H), 122.6 (*C*q), 122.0 (*C*H), 120.4 (*C*H), 115.2 (*C*H), 113.4 (*C*H), 111.5 (*C*H), 110.1 (*C*H), 55.1 (*C*H_3_); *m*/*z* (APCI+) 352 (MH^+^, 67%), 231 (52), 177 (46), 152 (98), 135 (82), 109 (100); HRMS (ESI+) found for MH^+^ 352.08557, C_17_H_14_N_5_O_2_S^+^ requires 352.08682.

*(2-Fluoropyridin-4-yl)-3-[(1*H*-indazol-5-yl)amino]-4*H*-1,2,6-thiadiazin-4-one* (**26**). Similar treatment of 5-chloro-3-[(1*H*-indazol-5-yl)amino]-4*H*-1,2,6-thiadiazin-4-one (**13**) (55.9 mg, 0.20 mmol) with (2-fluoropyridin-4-yl)boronic acid (56.4 mg, 0.40 mmol) for 1 h gave after chromatography (DCM/*t*-BuOMe, 50:50) the *title compound* **26** (54.3 mg, 92%) as orange needles, mp 280 °C (decomp., from EtOH/CHCl_3_); R*_f_* 0.79 (DCM/*t*-BuOMe, 50:50); (found: C, 52.80; H, 2.72; N, 24.42. C_15_H_9_FN_6_OS requires C, 52.94; H, 2.67; N, 24.69%); *λ*_max_(DCM)/nm 252 (log *ε* 4.02), 322 (3.80), 384 (3.99), 453 inf (3.68); *v*_max_/cm^−1^ 3319w and 3221 br (N-H), 1620m, 1612m, 1587m, 1560m, 1557s, 1551s, 1547s, 1503s, 1483m, 1381m, 1352w, 1288m, 1223m, 1202w, 1161m, 1007m, 937m, 837s, 799s; *δ*_H_(500 MHz; DMSO*-d*_6_) 13.10 (1H, s, N*H*), 10.41 (1H, s, N*H*), 8.38 (1H, d, *J* 5.3, Ar *H*), 7.24 (1H, d, *J* 1.2, Ar *H*), 8.09 (1H, s, Ar *H*), 8.01 (1H, d, *J* 5.2, Ar *H*), 7.83 (1H, s, Ar *H*), 7.70 (1H, dd, *J* 8.9, 1.8, Ar *H*), 7.55 (1H, d, *J* 8.9, Ar *H*); *δ*_C_(125 MHz; DMSO*-d*_6_) 163.3 (*C*q, d, ^1^*J*_CF_ 232.5), 159.7 (*C*q), 153.1 (*C*q), 147.8 (*C*H, d, ^3^*J*_CF_ 15.3), 147.5 (*C*q, d, ^3^*J*_CF_ 8.7), 146.3 (*C*q, d, ^4^*J*_CF_ 3.7), 137.3 (*C*q), 133.6 (*C*H), 130.5 (*C*q), 122.6 (*C*q), 122.2 (*C*H), 120.0 (*C*H, d, ^4^*J*_CF_ 3.7), 112.2 (*C*H), 110.2 (*C*H), 107.3 (*C*H, d, ^2^*J*_CF_ 40.3); *m*/*z* (APCI+) 341 (MH^+^, 100%); HRMS (ESI+) found for MH^+^ 341.06105, C_15_H_10_FN_6_OS^+^ requires 341.06208.

#### 4.4.4. Preparation of 3,5-Diaminosubstituted Thiadiazines

*5-(4-Methylpiperazin-1-yl)-3-[(1*H*-pyrrolo[2,3-*b*]pyridin-4-yl)amino]-4*H*-1,2,6-thiadiazin-4-one* (**21**) (general procedure). To a mixture of 3-chloro-5-(4-methylpiperazin-1-yl)-4*H*-1,2,6-thiadiazin-4-one (**12**) (49.4 mg, 0.20 mmol), Pd[3,5-(F_3_C)_2_C_6_H_3_]_3_ (5.3 mg, 1.25 mol%), DPEPhos (5.3 mg, 5 mol%), powdered dry K_2_CO_3_ (66.4 mg, 0.48 mmol) and 1*H*-pyrrolo[2,3-*b*]pyridin-4-amine (29.3 mg, 0.22 mmol) was added dry dioxane (4 mL). The stirred suspension was then deaerated by bubbling of Ar through it for 5 min and then heated at reflux under Ar until complete consumption of the starting thiadiazine (TLC, 2 h). The mixture was cooled to ca. 20 °C, then filtered and washed with H_2_O (5 mL) and EtOH (5 mL), and dried under vacuum to give the *title compound* **21** (43.6 mg, 64%) as yellow plates, mp 286–287 °C (from *c*-hexane/CHCl_3_); R*_f_* 0.30 (THF); (found: C, 52.41; H, 4.83; N, 28.47. C_15_H_17_N_7_OS requires C, 52.46; H, 4.99; N, 28.55%); *λ*_max_(DCM)/nm 272 (log *ε* 4.06), 338 (4.71), 351 (4.71), 442 (4.17); *v*_max_/cm^−1^ 3130w, 3022w, 2961w, 1841w, 2792w, 1611m, 1587s, 1549m, 1535m, 1503m, 1485m, 1479m, 1443m, 1439m, 1402m, 1333m, 1306m, 1290m, 1271m, 1252w, 1225m, 1207w, 1161m, 1121m, 951w, 899m, 858w, 818m, 787m, 781m, 773m, 743m, 704m; *δ*_H_(500 MHz; DMSO*-d*_6_) 11.66 (1H, s, N*H*), 9.28 (1H, s, N*H*), 8.13 (1H, d, *J* 5.4, Ar *H*), 7.73 (1H, d, *J* 5.4, Ar *H*), 7.38 (1H, dd, *J* 2.9, 2.9, Ar *H*), 6.61 (1H, dd, *J* 3.3, 1.8, Ar *H*), 3.64 (4H, br. s, C*H*_2_), 2.25 (3H, s, C*H*_3_), 4H missing due to overlap with H_2_O peak (2×C*H*_2_); *δ*_C_(125 MHz; DMSO*-d*_6_) 156.7 (*C*q), 151.6 (*C*q), 149.4 (*C*q), 148.6 (*C*q), 143.6 (*C*H), 137.7 (*C*q), 124.2 (*C*H), 110.2 (*C*q), 103.4 (*C*H), 97.0 (*C*H), 54.0 (*C*H_2_), 45.9 (*C*H_2_), 45.4 (*C*H_3_); *m*/*z* (MALDI-TOF) 344 (MH^+^, 100%), 287 (95); HRMS found for MH^+^ 344.12803, C_15_H_18_N_7_OS^+^ requires 344.12935.

*3-[(1*H*-Indazol-5-yl)amino]-5-(4-methylpiperazin-1-yl)-4*H*-1,2,6-thiadiazin-4-one* (**22**). Similar treatment of 3-chloro-5-(4-methylpiperazin-1-yl)-4*H*-1,2,6-thiadiazin-4-one (**12**) (49.4 mg, 0.20 mmol), with 1*H*-indazol-5-amine (29.3 mg, 0.22 mmol) after 16 h gave after chromatography (DCM/*t*-BuOMe/Et_3_N, 50:49:1) the *title compound* **22** (65.2 mg, 92%) as yellow needles, mp 262–263 °C (from EtOH/THF); R*_f_* 0.16 (DCM/*t*-BuOMe/Et_3_N, 50:49:1); (found: C, 52.70; H, 4.78; N, 28.62. C_15_H_17_N_7_OS requires C, 52.46; H, 4.99; N, 28.55%); *λ*_max_(THF)/nm 263 (log *ε* 4.30), 308 (3.69), 469 (3.00); *v*_max_/cm^−1^ 3339w, 1601m, 1585m, 1557m, 1503m, 1493m, 1437w, 1404w, 1306w, 1287w, 1277w, 1246w, 1221m, 1128m, 1078m, 1053s, 978m, 945m, 870m, 799s, 733m; *δ*_H_(500 MHz; DMSO*-d*_6_) 13.01 (1H, br. s, N*H*), 9.65 (1H, s, N*H*), 8.21 (1H, s, Ar *H*), 8.03 (1H, s, Ar *H*), 7.61 (1H, dd, *J* 9.0, 1.8, Ar *H*), 7.49 (1H, d, *J* 9.0, Ar *H*), 3.66 (4H, m, C*H*_2_), 2.86 (4H, m, C*H*_2_), 3H missing due to overlap with DMSO peak (1×C*H*_3_); *δ*_C_(125 MHz; DMSO*-d*_6_) 156.8 (*C*q), 151.0 (*C*q), 149.9 (*C*q), 136.9 (*C*q), 133.2 (*C*H), 131.8 (*C*q), 122.6 (*C*q), 121.7 (*C*H), 110.2 (*C*H), 109.9 (*C*H), 53.1 (*C*H_2_), 45.0 (*C*H_2_), 44.1 (*C*H_3_); *m*/*z* (MALDI-TOF) 344 (MH^+^, 100%), 287 (68); HRMS found for MH^+^ 344.12806, C_15_H_18_N_7_OS^+^ requires 344.12935.

## Figures and Tables

**Table 1:** Activity of anilinothiadiazinones on a panel of seven cancer cell lines.

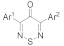										
Compound	Ar^1^	Ar^2^	5637	DU145	PANC1	MCF7	UCH1	UCH2	A431	WS-1
			IC_50_ (μM) ^a^							
**14**	** 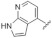 **	** 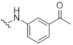 **	15	>100	>100	>100	58	>100	>100	92
**15**		** 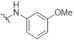 **	15	>100	>100	>100	67	>100	>100	>100
**16**		** 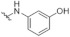 **	1.6	41	33	18	46	>100	>100	>100
**17**		** 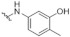 **	2.1	23	>100	14	>100	>100	>100	>100
**18**		** 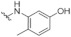 **	55	>100	>100	>100	>100	>100	>100	>100
**19**		** 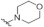 **	>100	14	11	46	>100	>100	>100	>100
**20**		** 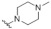 **	>100	>100	>100	>100	nt	nt	nt	nt
**21**	** 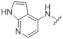 **		>100	51	>100	>100	97	>100	>100	>100
**22**	** 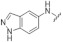 **		>100	50	>100	25	>100	>100	>100	24
**23**		** 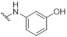 **	>100	84	>100	92	71	>100	>100	>100
**24**		** 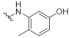 **	>100	15	26	13	67	>100	>100	>100
**25**		** 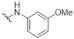 **	13	>100	>100	38	52	99	>100	71
**26**		** 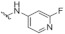 **	8.4	5.7	>100	50	84	>100	>100	>100

^a^ Average of 4 independent experiments, nt = not tested.

## Data Availability

Not applicable.

## Supplementary Materials

The following are available online, mol file, HRMS spectra on the final compounds, ^1^H and ^13^C NMR spectra on all new compounds.

## Sample Availability

Samples of the compounds are available from the authors.
