# Supplementary material for: Synthesis and Evaluation of Novel 1,2,6-Thiadiazinone Kinase Inhibitors as Potent Inhibitors of Solid Tumors
Source: Molecules. 2021 Sep 29;26(19):5911. doi: 10.3390/molecules26195911 (PMC8513058; doi:10.3390/molecules26195911)
Supplement: Supplementary file 1 [file molecules-26-05911-s001.zip › molecules-1390362-supplementary.pdf]

Supplementary Materials

Synthesis and Evaluation of Novel 1,2,6-Thiadiazinone Kinase Inhibitors as Potent Inhibitors of Solid Tumors

Andreas S. Kalogirou [1,2,*], Michael P. East [3], Tuomo Laitinen [4], Chad D. Torrice [5], Kaitlyn A. Maffuid [5], David H. Drewry [6,7], Panayiotis A. Koutentis [2], Gary L. Johnson [3,7], Daniel J. Crona [5,7] and Christopher R. M. Asquith [3,*]

1 Department of Life Sciences, School of Sciences, European University Cyprus, 6 Diogenis Str., Engomi, P.O. Box 22006, Nicosia 1516, Cyprus

2 Department of Chemistry, University of Cyprus, P.O. Box 20537, Nicosia 1678, Cyprus; kouten-ti@ucy.ac.cy

3 Department of Pharmacology, School of Medicine, University of North Carolina, Chapel Hill, NC 27599, USA; Michael_East@med.unc.edu (M.P.E.); gary_johnson@med.unc.edu (G.L.J.)

4 School of Pharmacy, Faculty of Health Sciences, University of Eastern Finland, 70211 Kuopio, Finland; tuomo.laitinen@uef.fi

5 Division of Pharmacotherapy and Experimental Therapeutics, UNC Eshelman School of Pharmacy, University of North Carolina, Chapel Hill, NC 27599, USA; chad_torrice@med.unc.edu (C.D.T.); kmaffuid@email.unc.edu (K.A.M.); crona@email.unc.edu (D.J.C.)

6 Structural Genomics Consortium, UNC Eshelman School of Pharmacy, University of North Carolina, Chapel Hill, NC 27599, USA; david.drewry@unc.edu

7 Lineberger Comprehensive Cancer Center, School of Medicine, University of North Carolina, Chapel Hill, NC 27599, USA

* Correspondence: A.Kalogirou@euc.ac.cy (A.S.K.); chris.asquith@unc.edu (C.R.M.A.); Tel.: +357-22-559655 (A.S.K.); +1-919-491-3177 (C.R.M.A.)

Contents **Page**

S1.	High resolution mass spectra of final products	S3
S2.	^{1}H and ^{13}C NMR spectra of all new compounds	S16-S54

S1. High resolution mass spectra of final products

3-((3-Acetylphenyl)amino)-5-(1*H*-pyrrolo[2,3-*b*]pyridin-4-yl)-4*H*-1,2,6-thiadiazin-4-one (**14**)

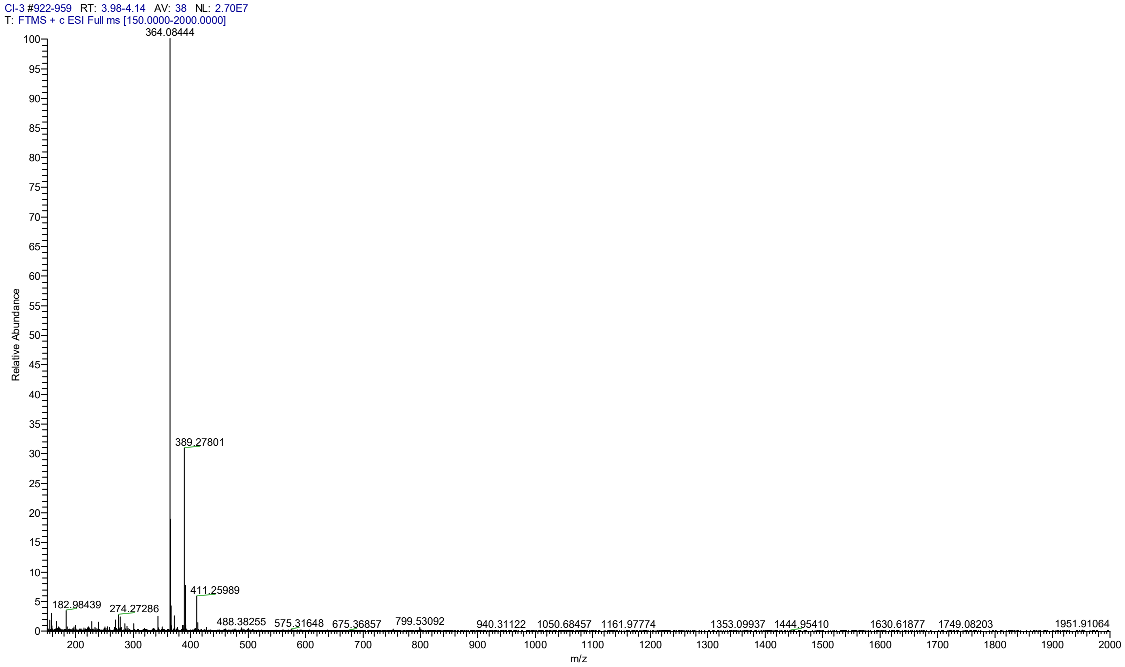

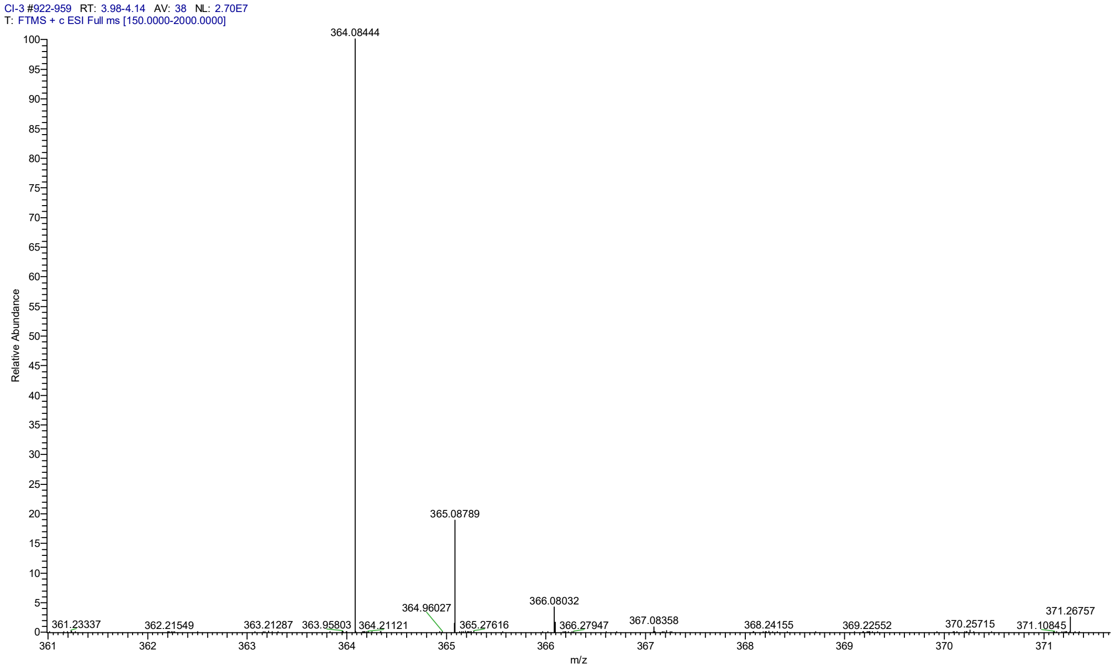

3-((3-Methoxyphenyl)amino)-5-(1*H*-pyrrolo[2,3-*b*]pyridin-4-yl)-4*H*-1,2,6-thiadiazin-4-one (**15**)

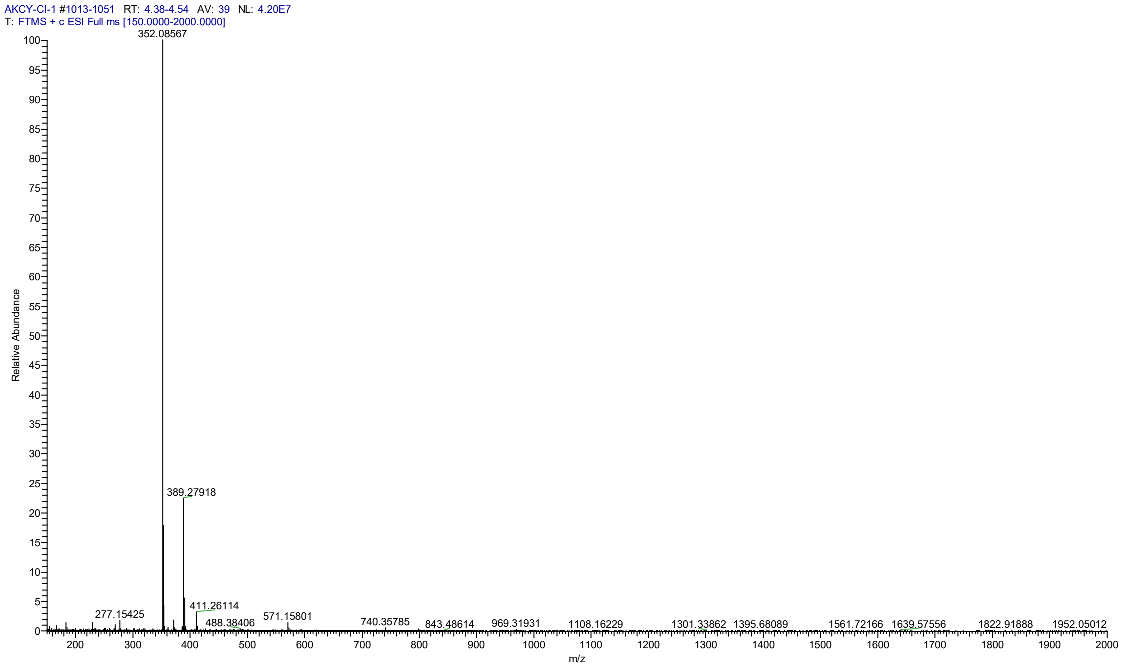

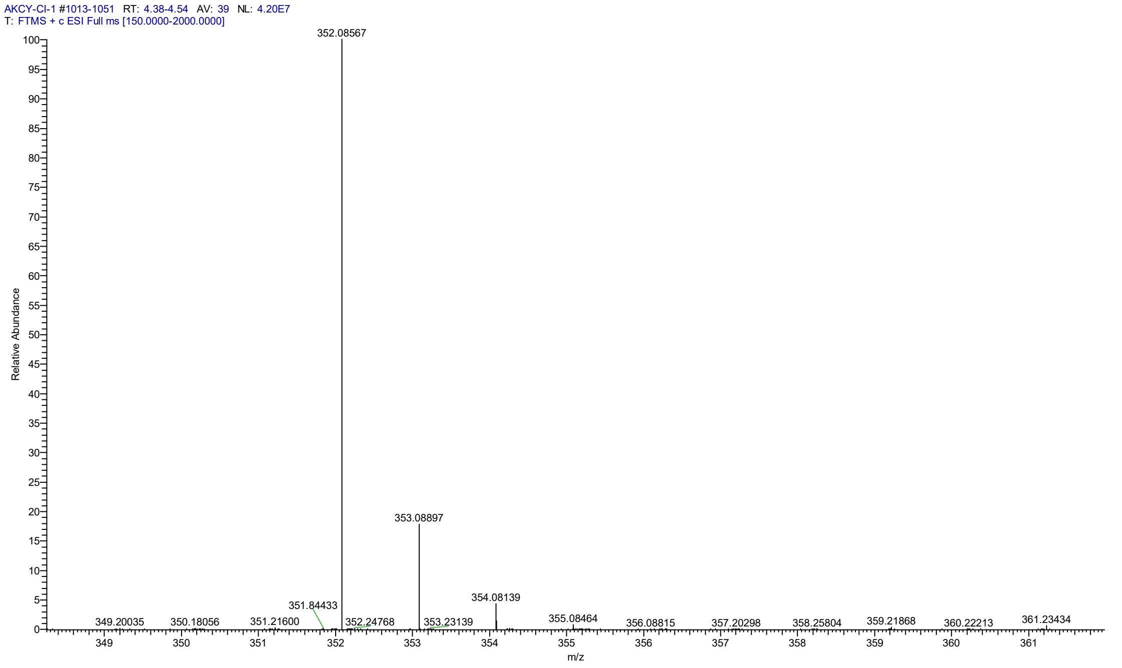

3-((3-Hydroxyphenyl)amino)-5-(1*H*-pyrrolo[2,3-*b*]pyridin-4-yl)-4*H*-1,2,6-thiadiazin-4-one (**16**)

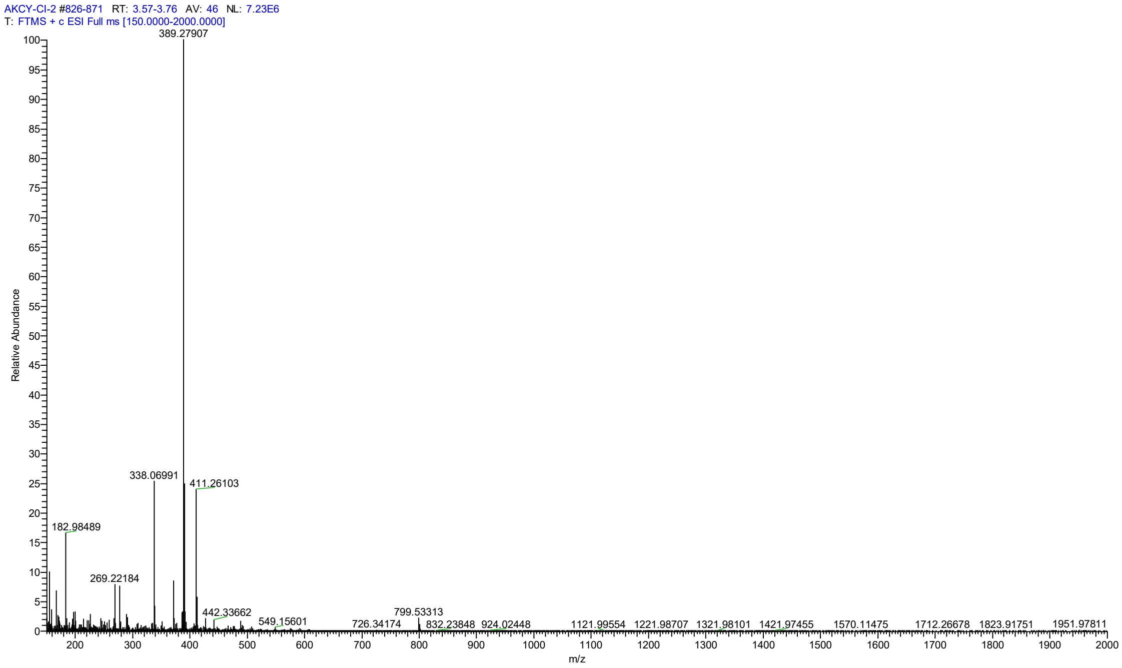

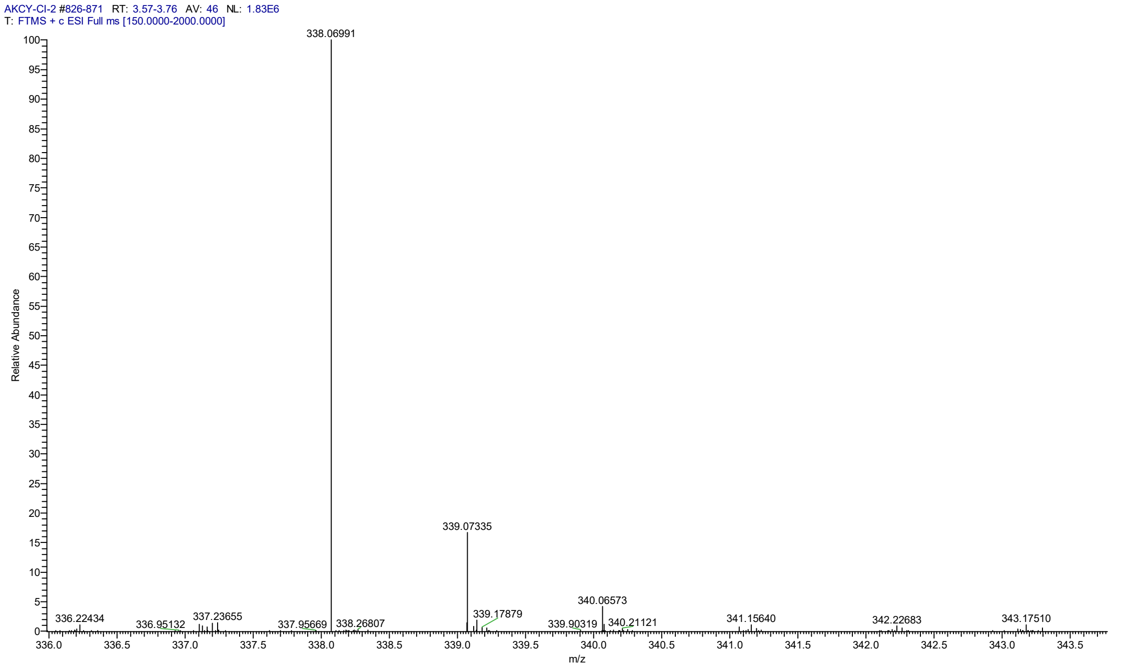

3-((3-Hydroxy-4-methylphenyl)amino)-5-(1*H*-pyrrolo[2,3-*b*]pyridin-4-yl)-4*H*-1,2,6-thiadiazin-4-one (**17**)

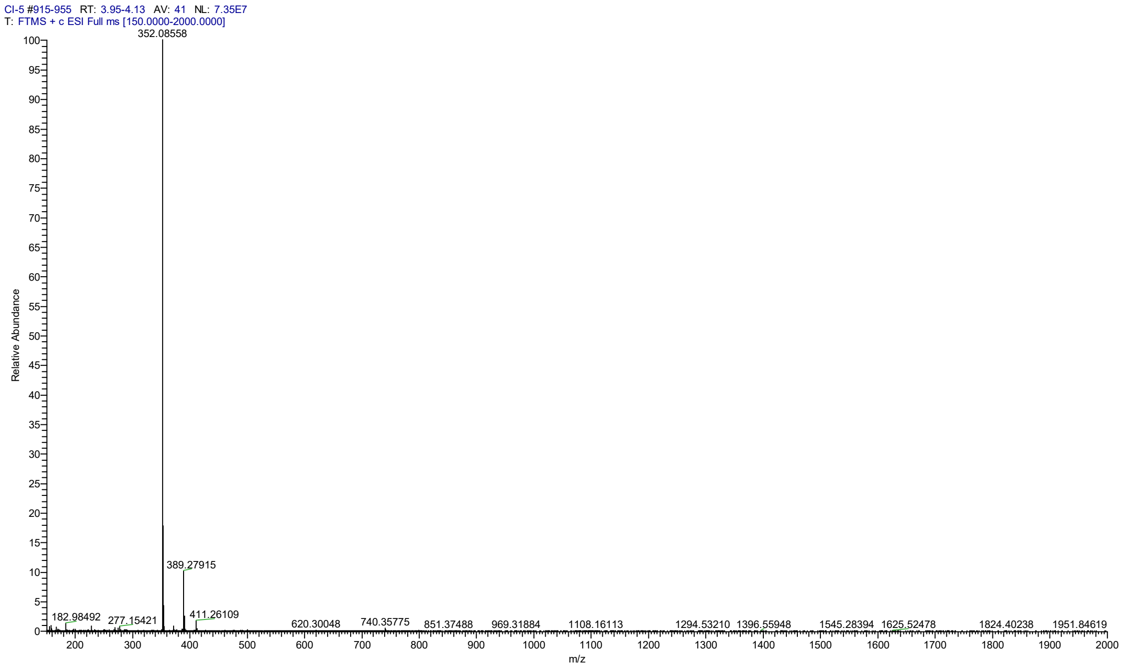

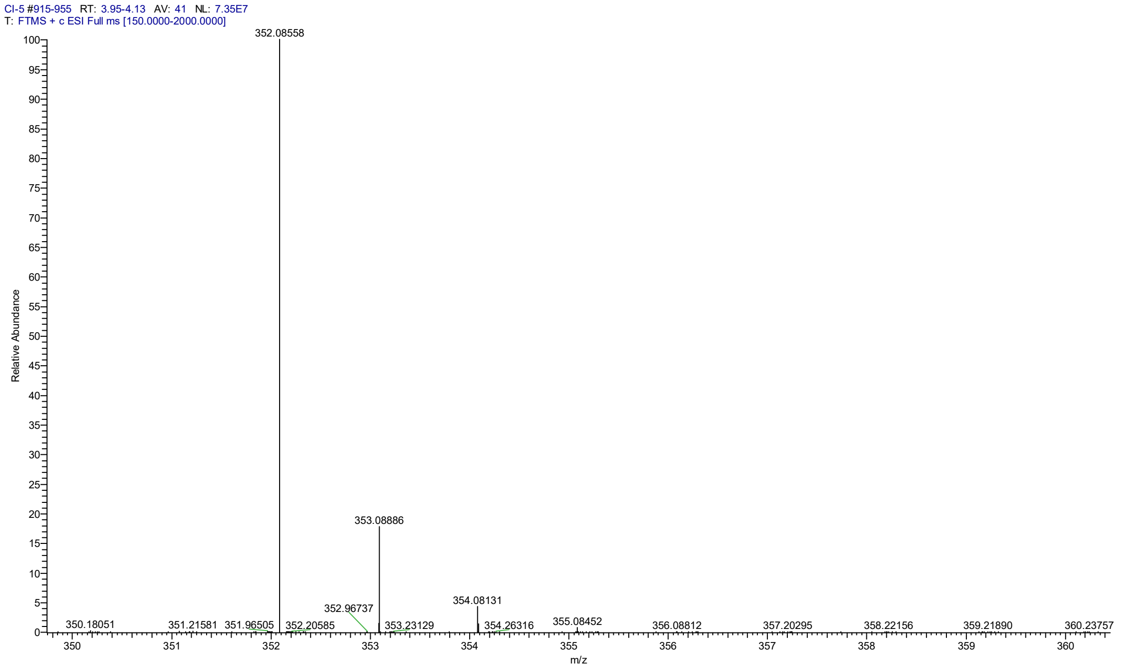

3-((5-Hydroxy-2-methylphenyl)amino)-5-(1*H*-pyrrolo[2,3-*b*]pyridin-4-yl)-4*H*-1,2,6-thiadiazin-4-one (**18**)

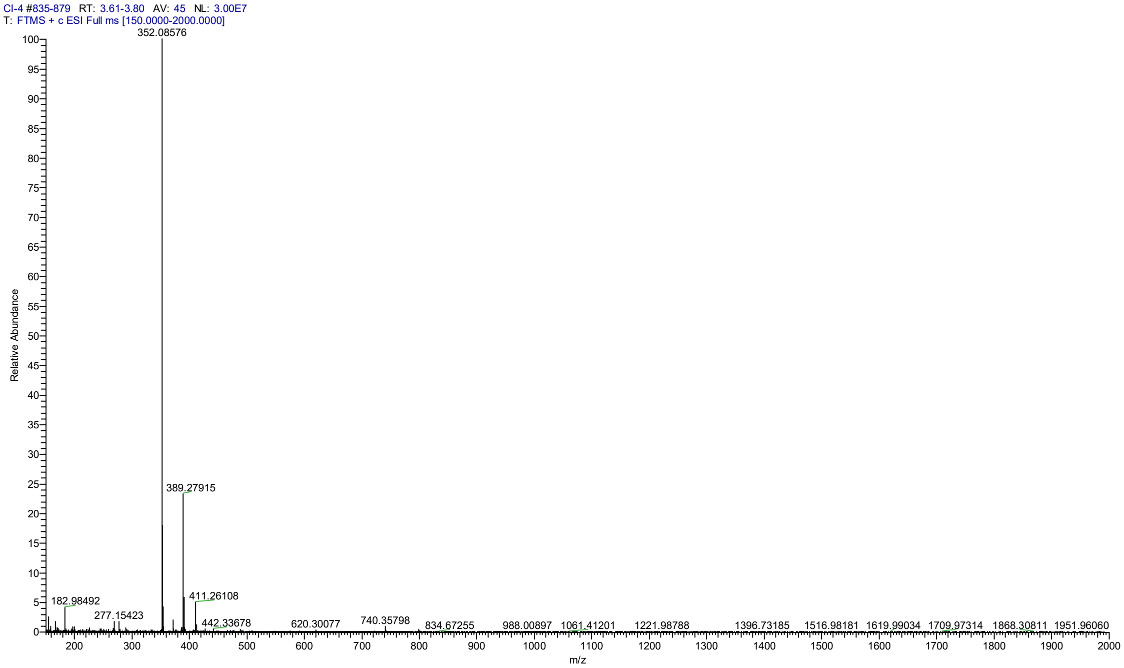

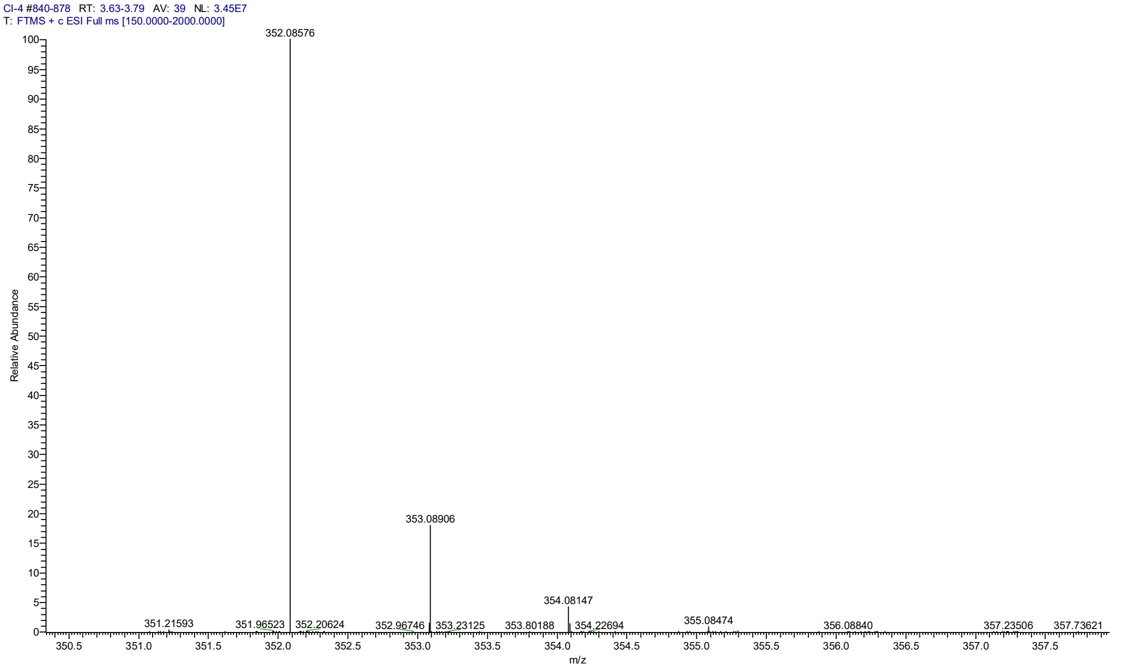

3-Morpholino-5-(1*H*-pyrrolo[2,3-*b*]pyridin-4-yl)-4*H*-1,2,6-thiadiazin-4-one (**19**)

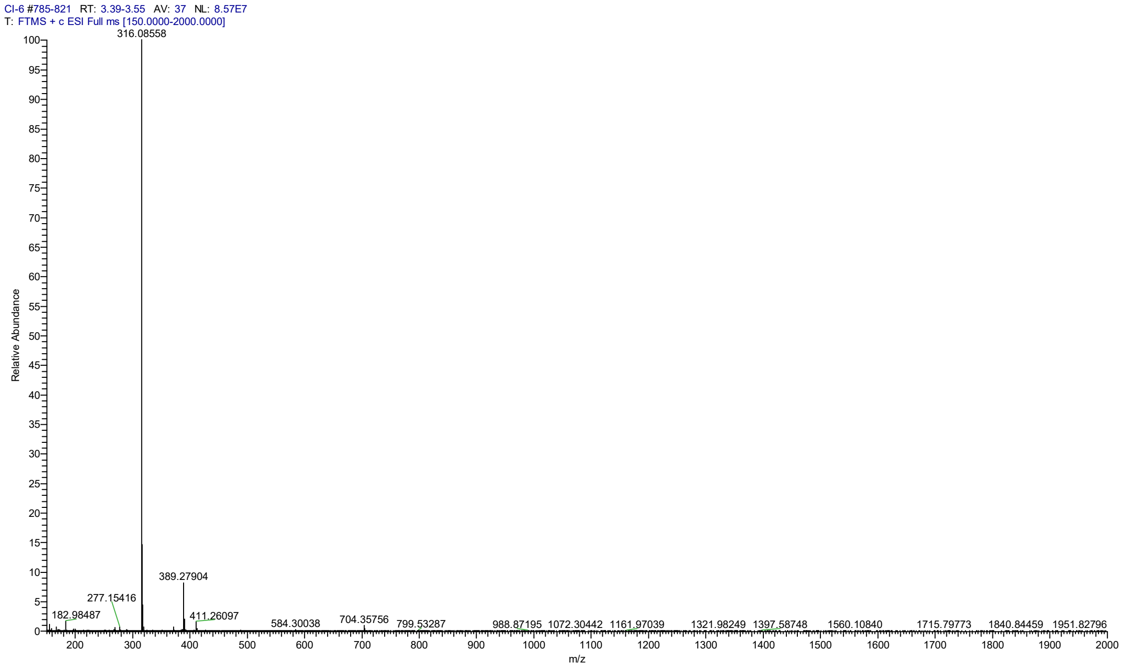

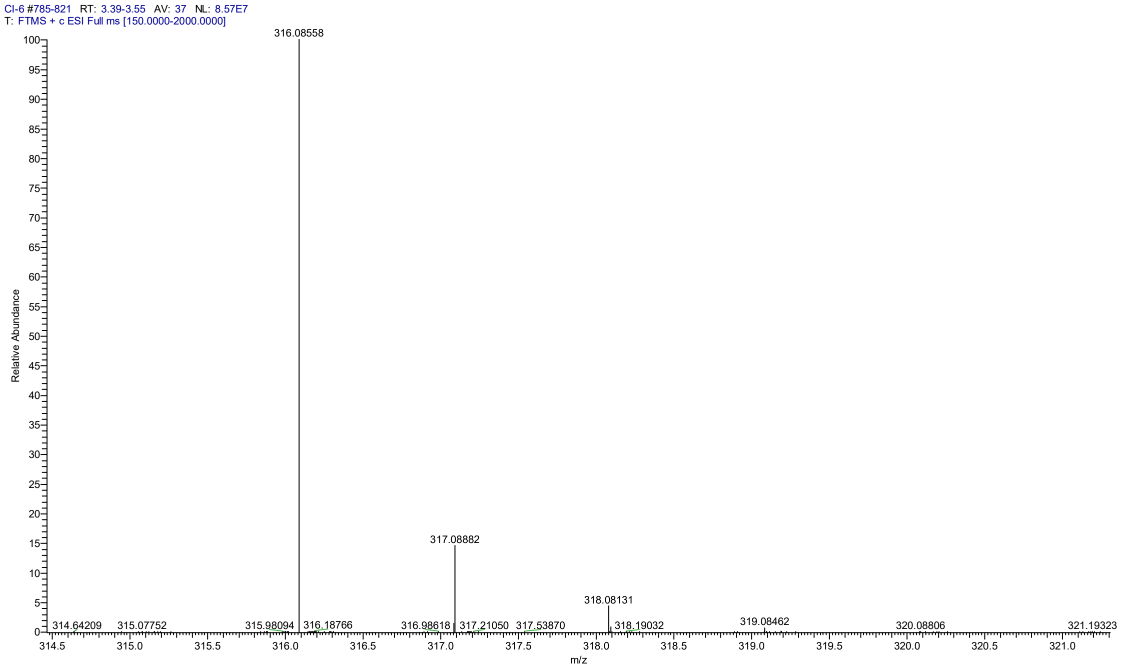

3-(4-Methylpiperazin-1-yl)-5-(1*H*-pyrrolo[2,3-*b*]pyridin-4-yl)-4*H*-1,2,6-thiadiazin-4-one (**20**).

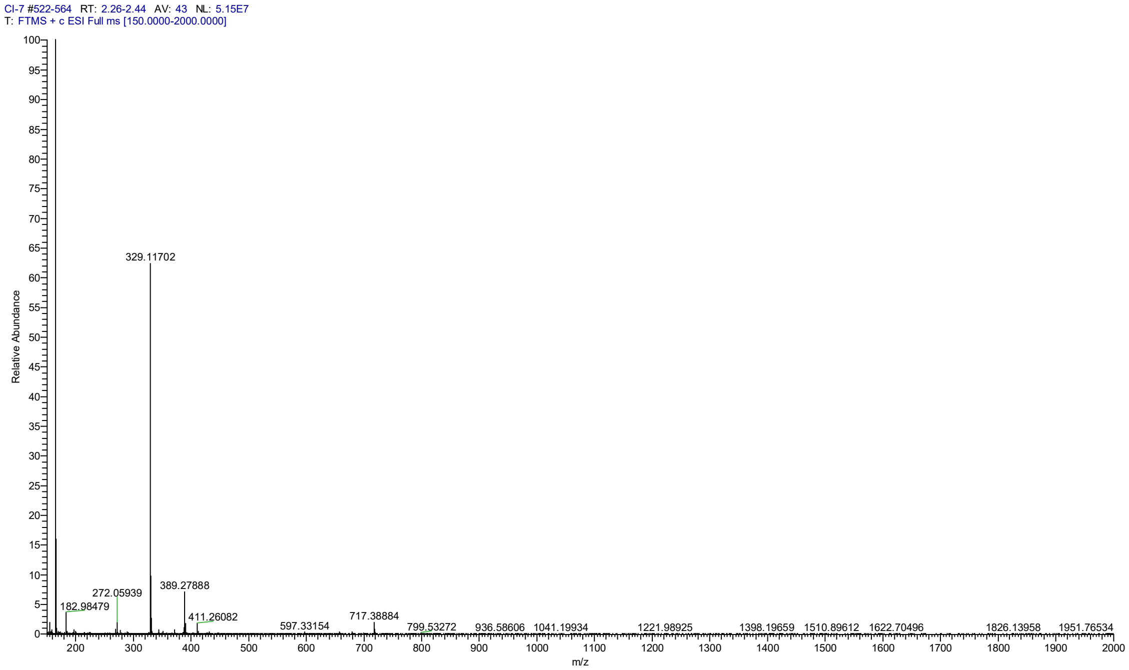

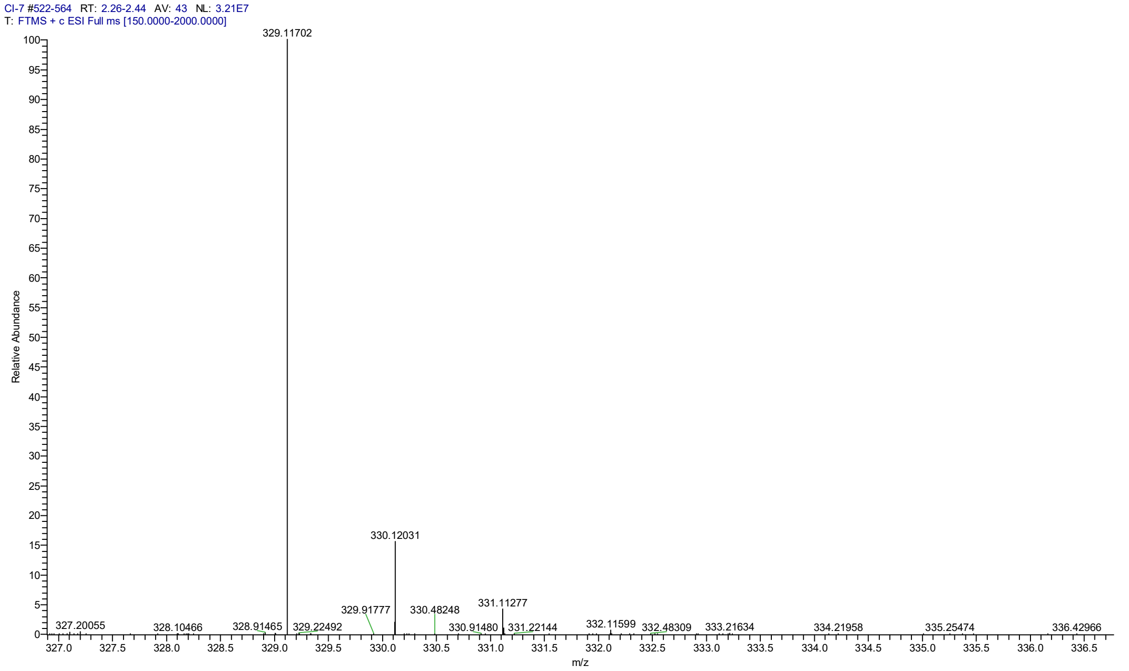

3-((1*H*-pyrrolo[2,3-*b*]pyridin-4-yl)amino)-5-(4-methylpiperazin-1-yl)-4*H*-1,2,6-thiadiazin-4-one (**21**)

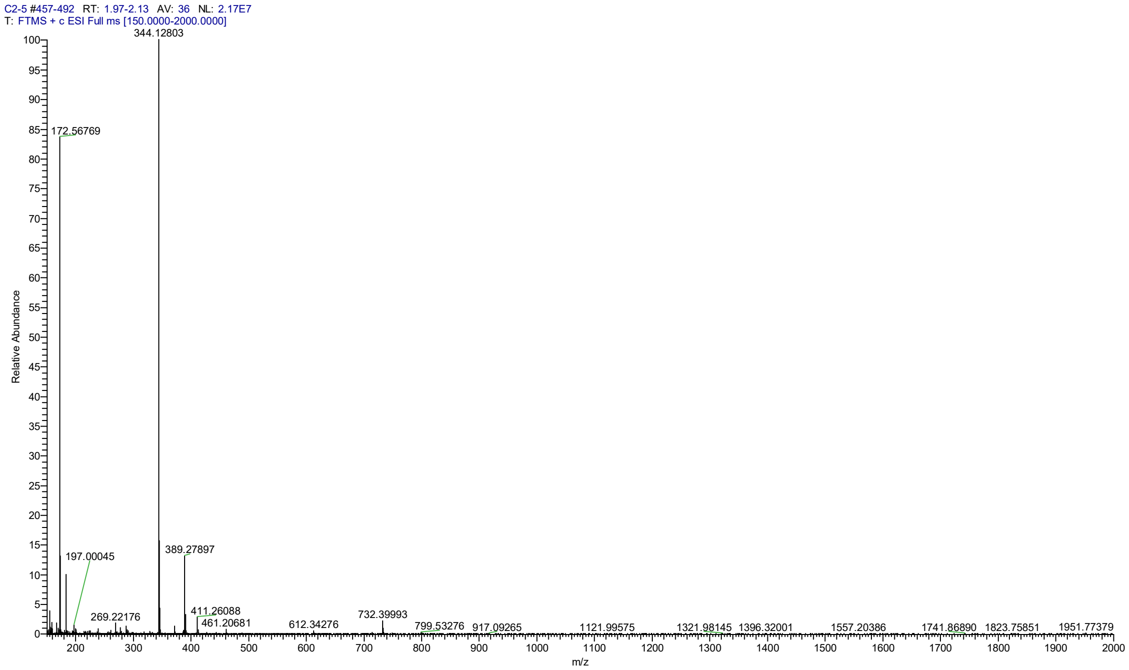

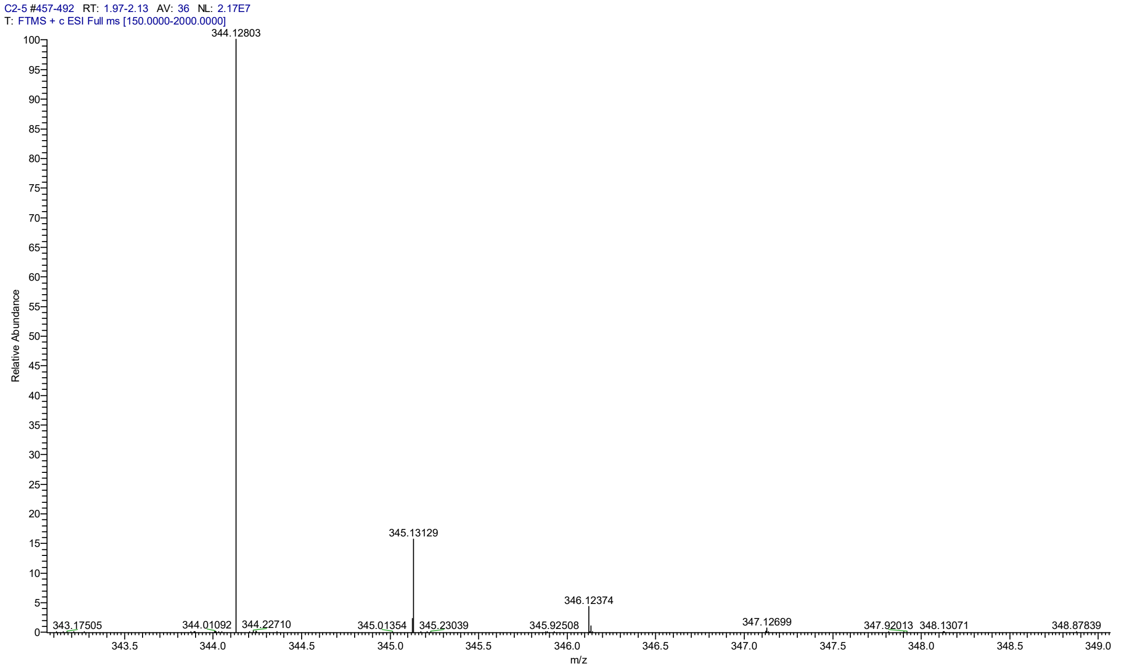

3-((1*H*-indazol-5-yl)amino)-5-(4-methylpiperazin-1-yl)-4*H*-1,2,6-thiadiazin-4-one (**22**)

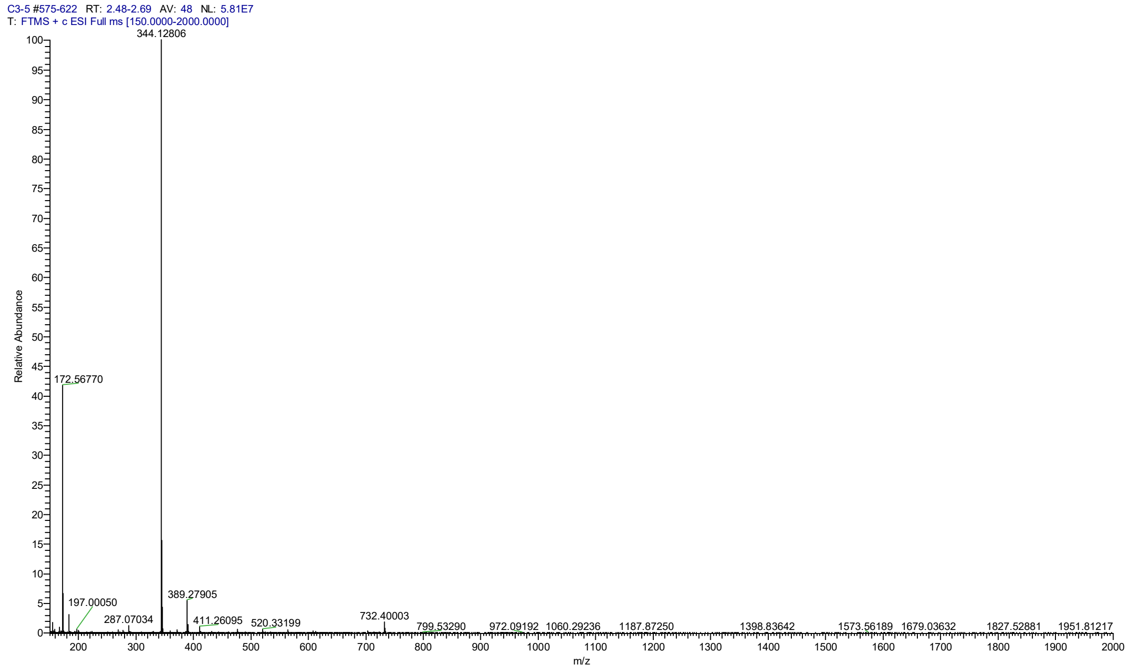

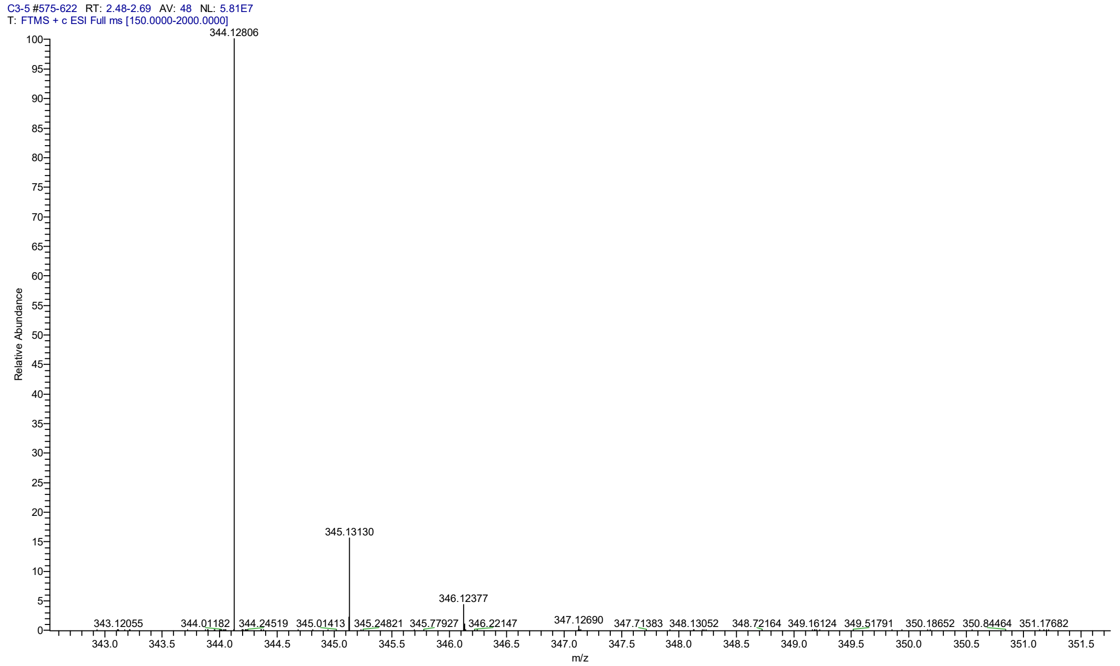

3-((1*H*-indazol-5-yl)amino)-5-(3-hydroxyphenyl)-4*H*-1,2,6-thiadiazin-4-one (**23**)

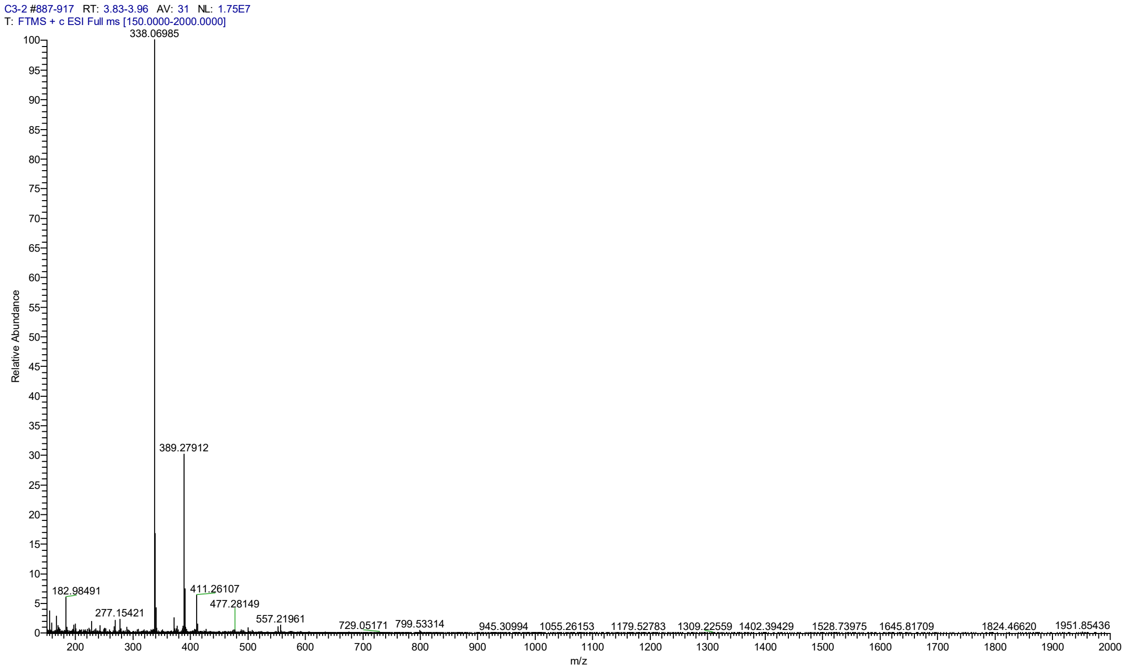

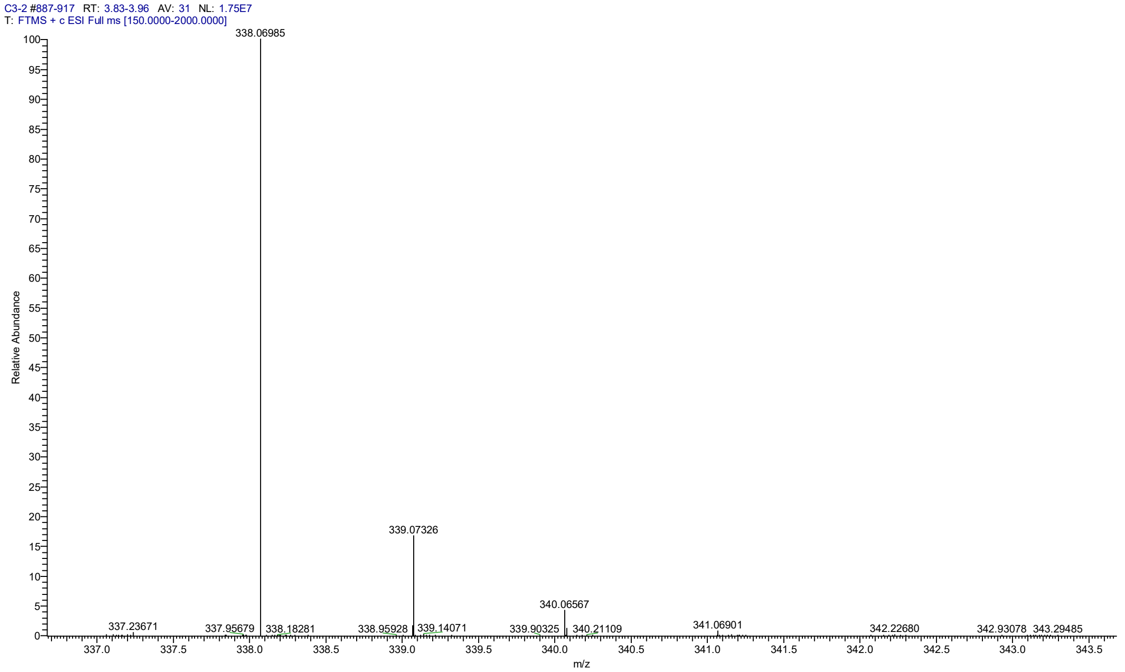

3-((1*H*-indazol-5-yl)amino)-5-(5-hydroxy-2-methylphenyl)-4*H*-1,2,6-thiadiazin-4-one (**24**)

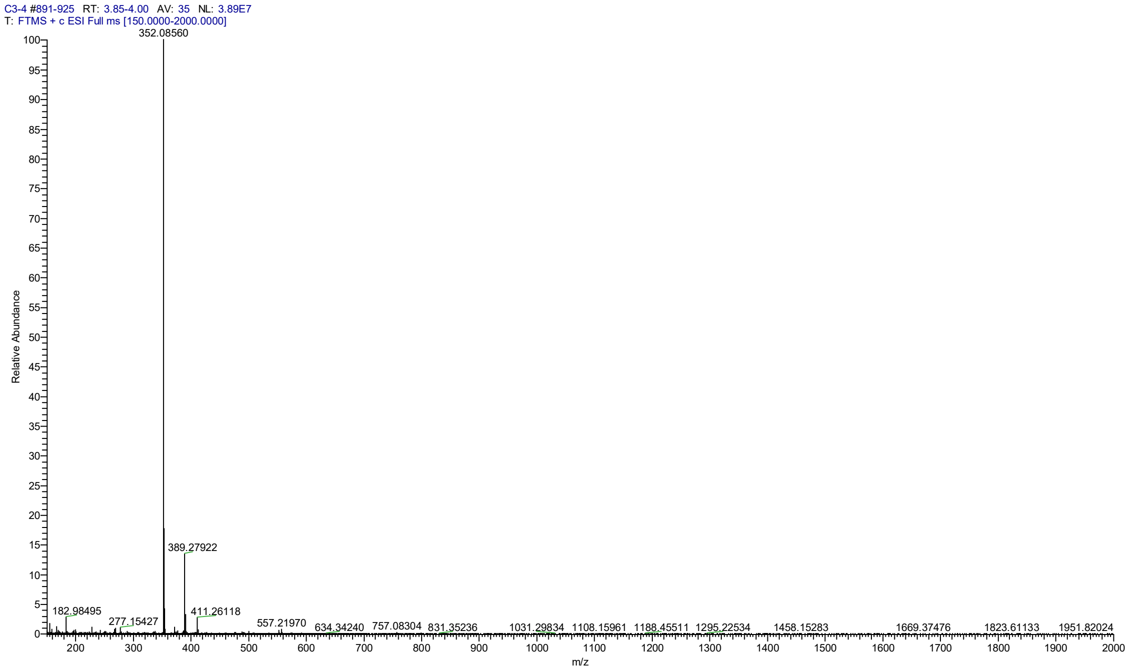

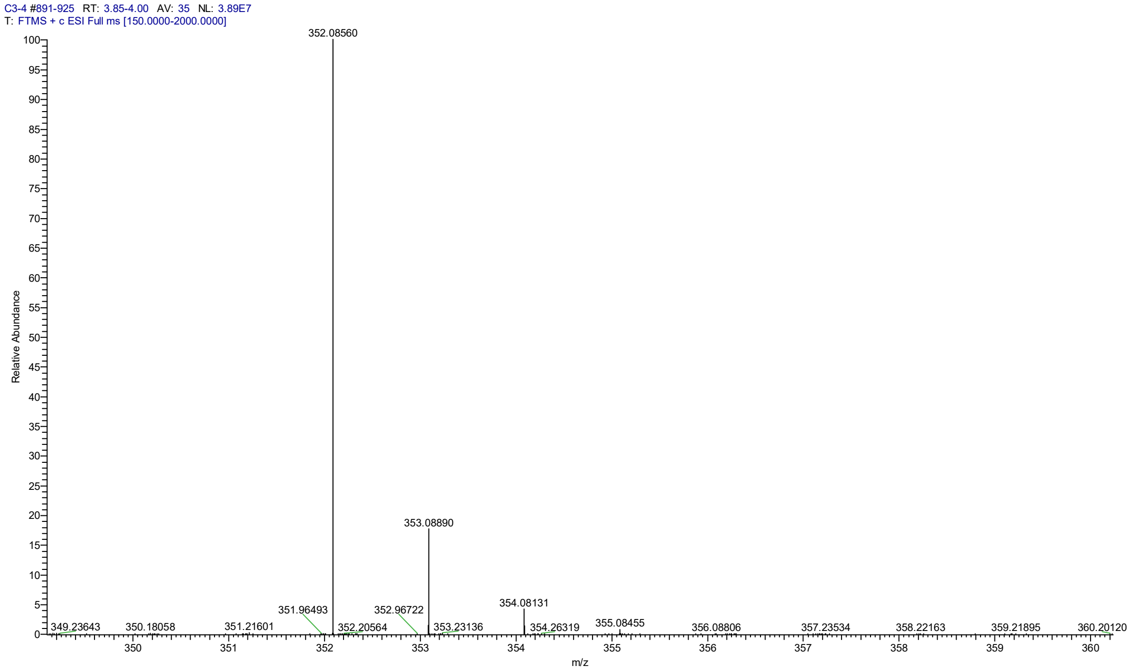

3-((1*H*-indazol-5-yl)amino)-5-(3-methoxyphenyl)-4*H*-1,2,6-thiadiazin-4-one (**25**)

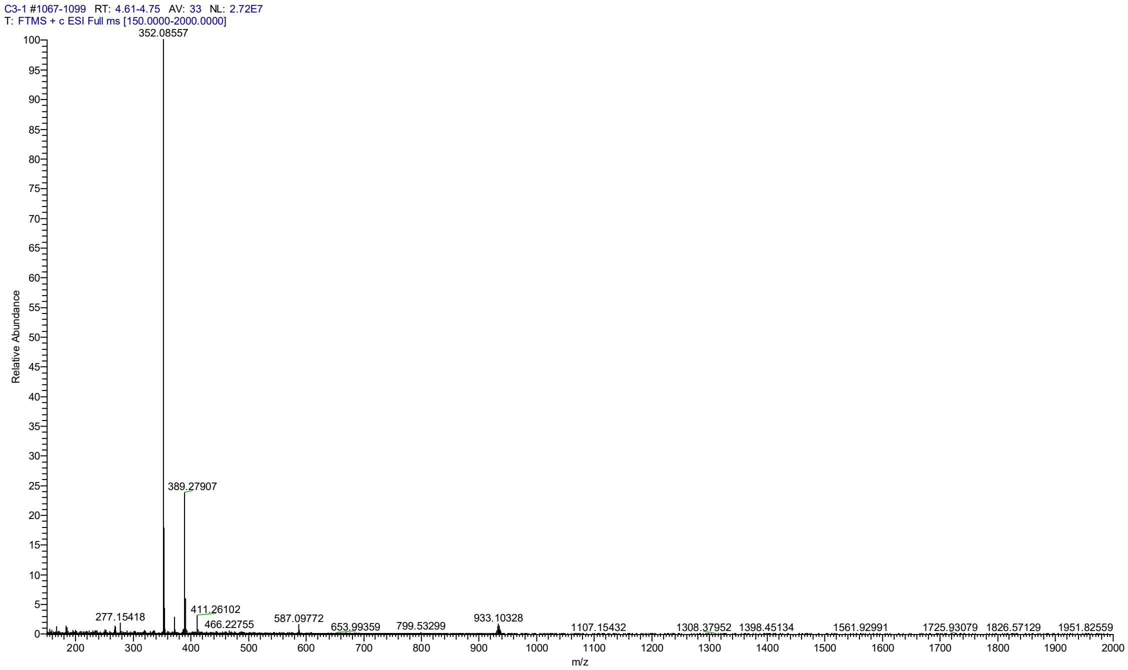

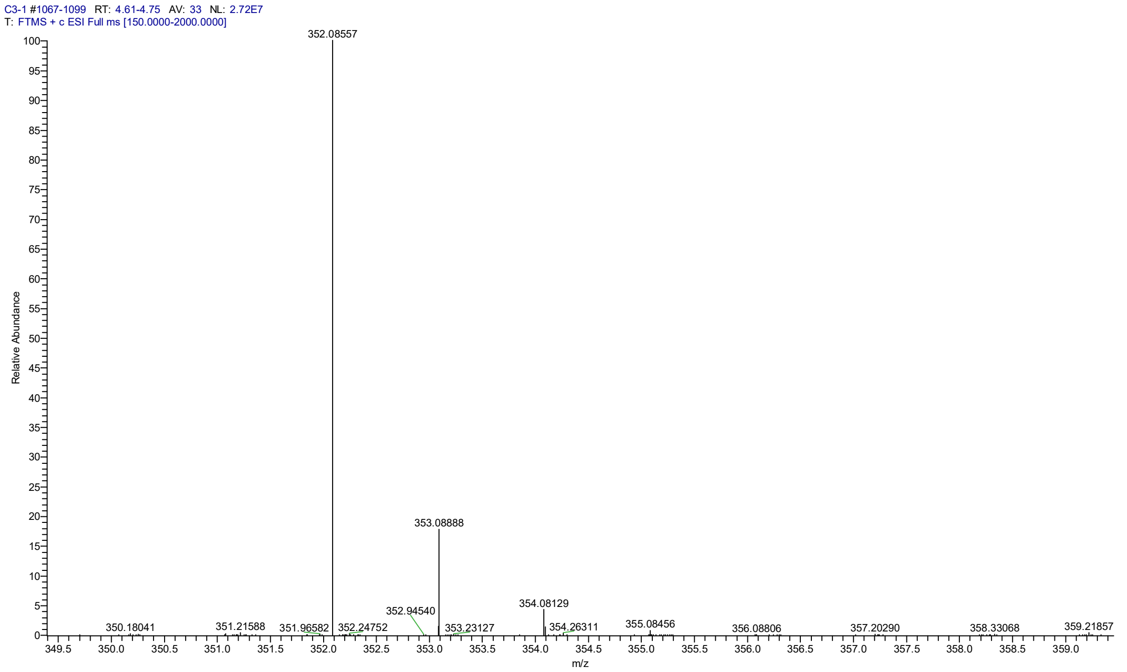

3-((1*H*-indazol-5-yl)amino)-5-(2-fluoropyridin-4-yl)-4*H*-1,2,6-thiadiazin-4-one (**26**)

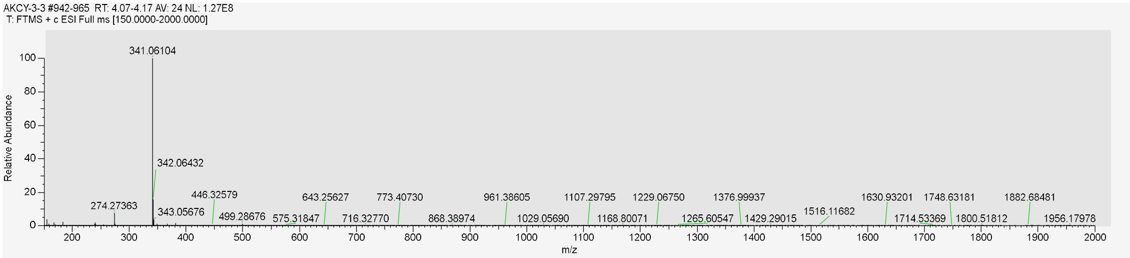

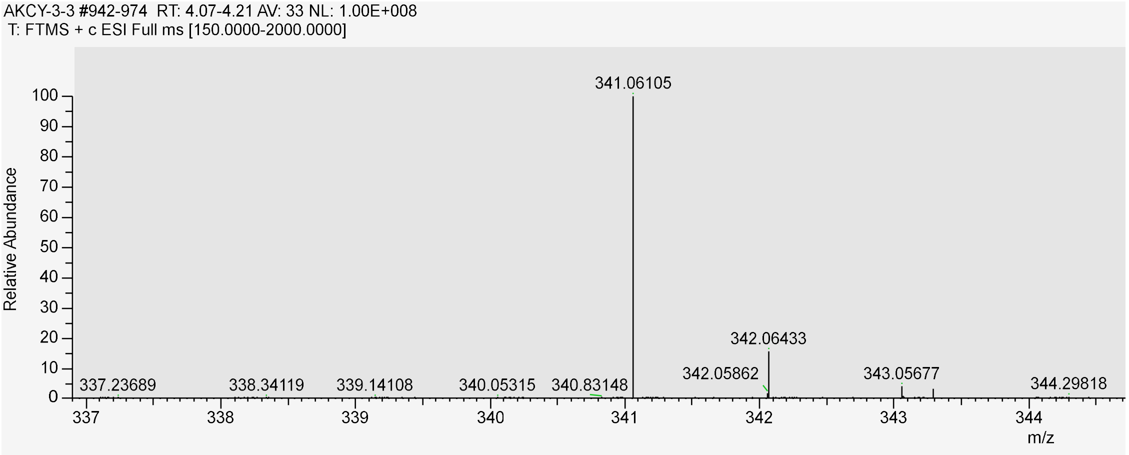

S2. ^{1}H and ^{13}C NMR spectra of all new compounds

^{1}H NMR of 3-((3-Acetylphenyl)amino)-5-chloro-4*H*-1,2,6-thiadiazin-4-one (**6**)

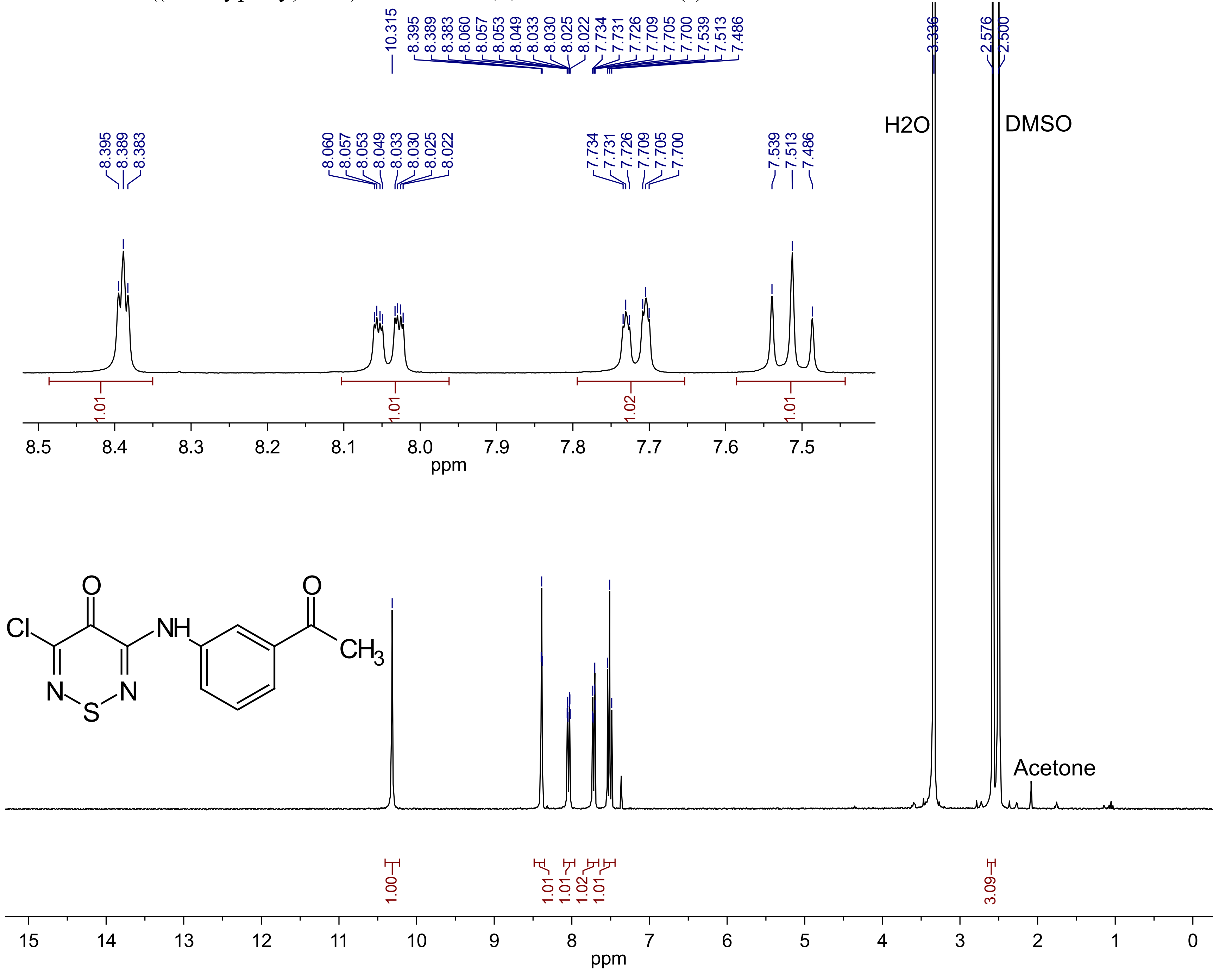

Current Data Parameters
NAME Andreas
EXPNO 82
PROCNO 1
F2 - Acquisition Parameters
Date_ 20171207
Time 19.16 h
INSTRUM spect
PROBHD Z104275_0375 (
PULPROG zg30
TD 65536
SOLVENT DMSO
NS 16
DS 2
SWH 6009.615 Hz
FIDRES 0.183399 Hz
AQ 5.4525952 sec
RG 201.81
DW 83.200 usec
DE 6.50 usec
TE 295.4 K
D1 1.00000000 sec
TD0 1
SFO1 300.1318533 MHz
NUC1 1H
P1 14.00 usec
PLW1 7.50000000 W
F2 - Processing parameters
SI 65536
SF 300.1300022 MHz
WDW EM
SSB 0
LB 0.30 Hz
GB 0
PC 1.00

^{13}C NMR of 3-((3-Acetylphenyl)amino)-5-chloro-4*H*-1,2,6-thiadiazin-4-one (**6**)

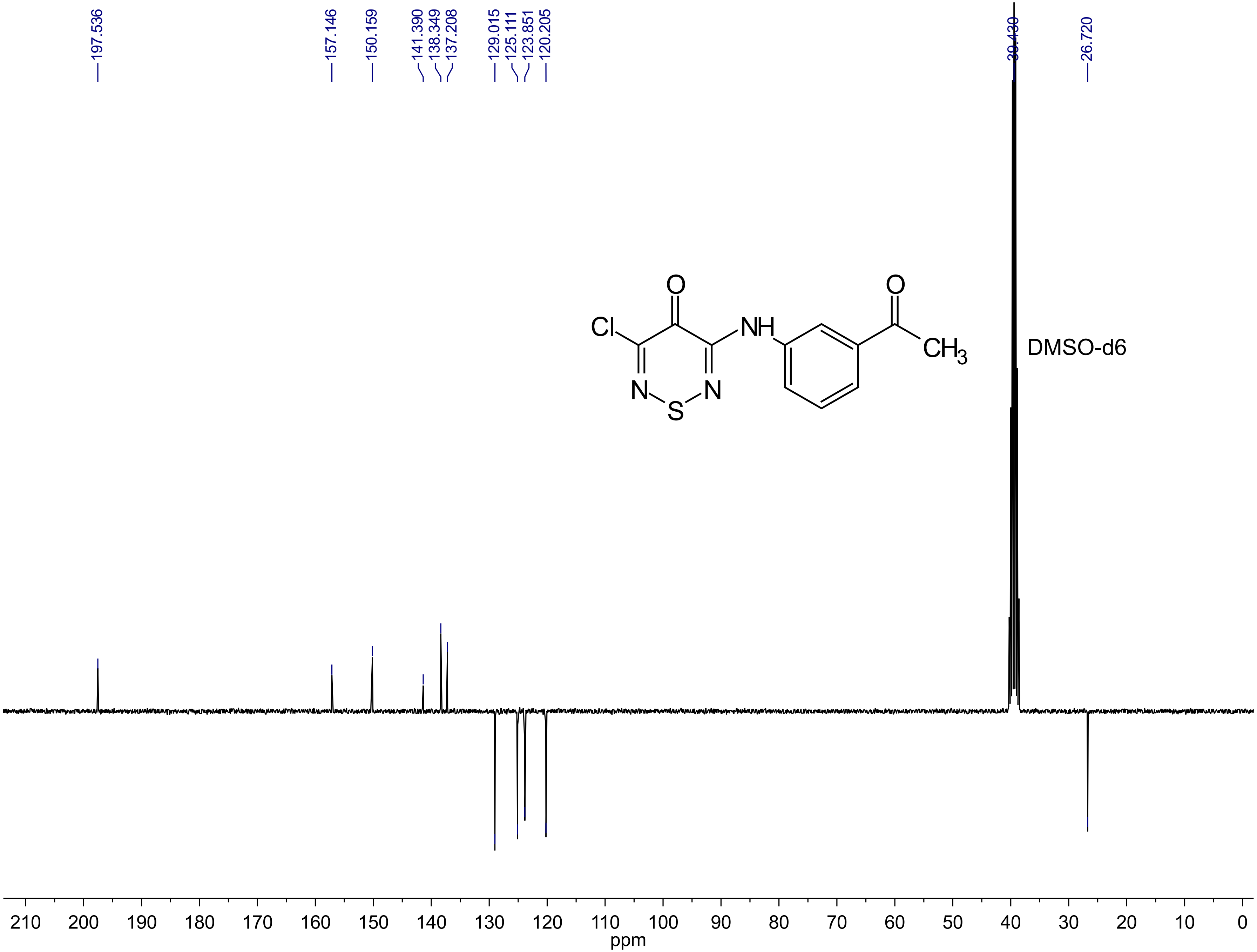

Current Data Parameters

NAME	Andreas
EXPNO	83
PROCNO	1

F2 - Acquisition Parameters

Date_	20171208
Time	9.51 h
INSTRUM	spect
PROBHD	Z104275_0375 (
PULPROG	jmod
TD	65536
SOLVENT	DMSO
NS	13514
DS	4
SWH	18115.941 Hz
FIDRES	0.552855 Hz
AQ	1.8087935 sec
RG	201.81
DW	27.600 usec
DE	6.50 usec
TE	295.7 K
CNST2	145.0000000
CNST11	1.0000000
D1	2.00000000 sec
D20	0.00689655 sec
TD0	1
SFO1	75.4752953 MHz
NUC1	13C
P1	10.00 usec
P2	20.00 usec
PLW1	40.05500031 W
SFO2	300.1312005 MHz
NUC2	1H
CPDPRG[2	waltz16
PCPD2	90.00 usec
PLW2	7.50000000 W
PLW12	0.18148001 W

F2 - Processing parameters

SI	32768
SF	75.4677900 MHz
WDW	EM
SSB	0
LB	1.00 Hz
GB	0
PC	1.40

^{1}H NMR of 3-Chloro-5-((3-methoxyphenyl)amino)-4*H*-1,2,6-thiadiazin-4-one (**7**)

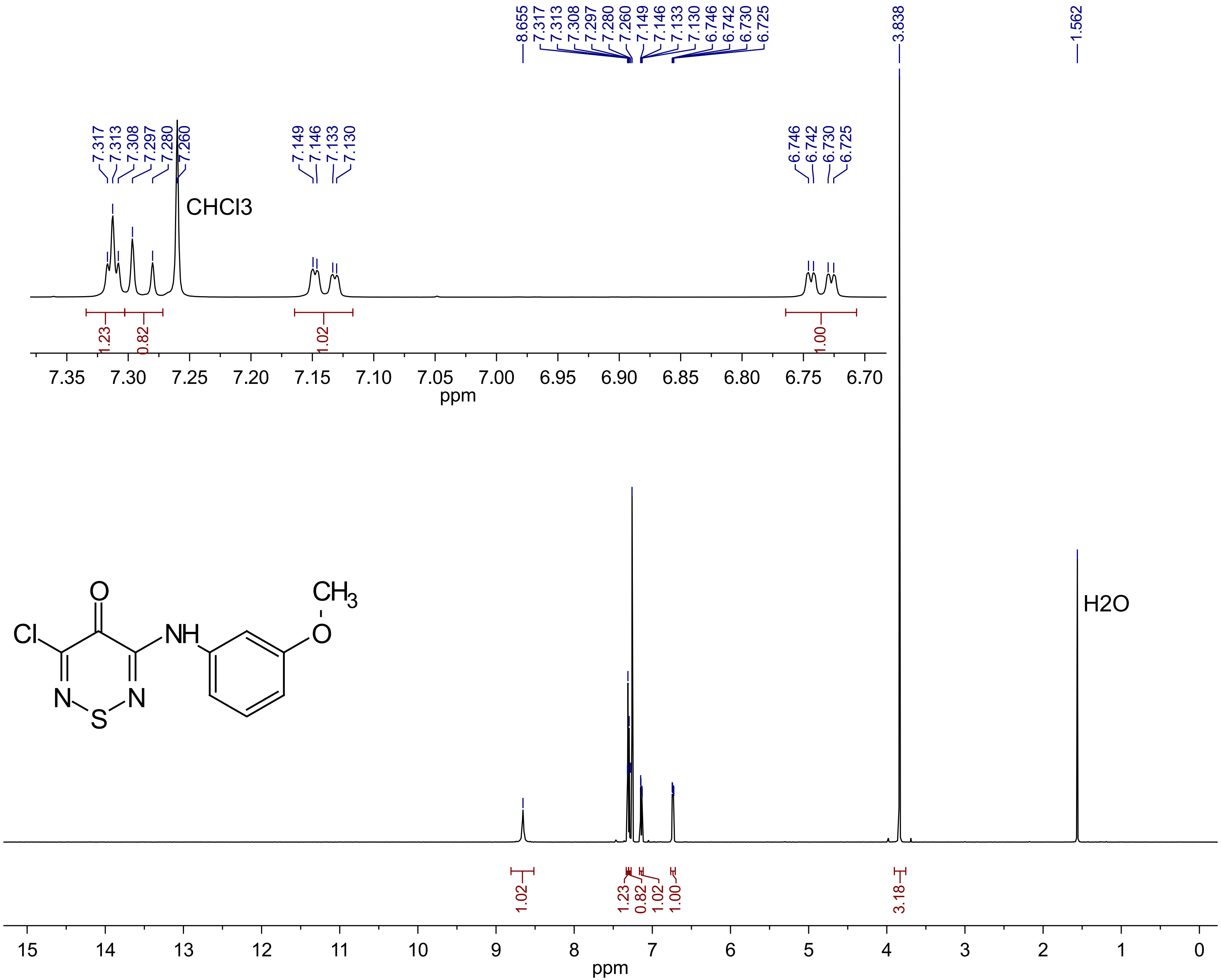

Current Data Parameters
NAME Kalogirou
EXPNO 463
PROCNO 1
F2 - Acquisition Parameters
Date_ 20171202
Time 15.16
INSTRUM spect
PROBHD 5 mm PABBO BB-
PULPROG zg30
TD 65536
SOLVENT CDCl3
NS 16
DS 2
SWH 10000.000 Hz
FIDRES 0.152588 Hz
AQ 3.2767999 sec
RG 181
DW 50.000 usec
DE 6.50 usec
TE 296.8 K
D1 1.00000000 sec
TD0 1
======== CHANNEL f1 ========
SFO1 500.0361158 MHz
NUC1 1H
P1 12.00 usec
PLW1 14.50000000 W
F2 - Processing parameters
SI 65536
SF 500.0330399 MHz
WDW EM
SSB 0
LB 0.30 Hz
GB 0
PC 1.00

^{13}C NMR of 3-Chloro-5-((3-methoxyphenyl)amino)-4*H*-1,2,6-thiadiazin-4-one (**7**)

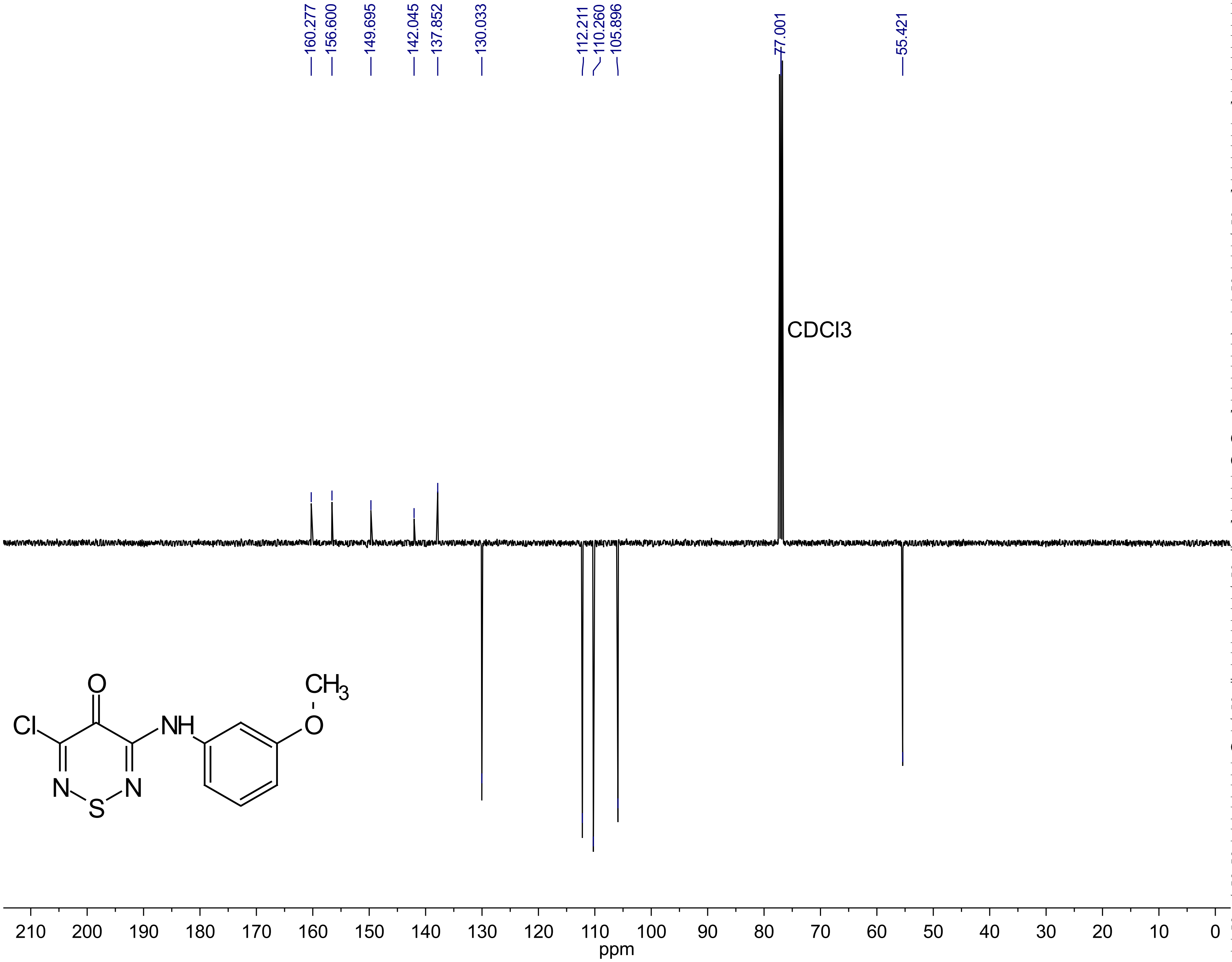

Current Data Parameters
NAME Kalogirou
EXPNO 464
PROCNO 1

F2 - Acquisition Parameters
Date_ 20171202
Time 17.27
INSTRUM spect
PROBHD 5 mm PABBO BB-
PULPROG jmod
TD 65536
SOLVENT CDCl3
NS 5632
DS 4
SWH 29761.904 Hz
FIDRES 0.454131 Hz
AQ 1.1010048 sec
RG 2050
DW 16.800 usec
DE 6.50 usec
TE 297.6 K
CNST2 145.0000000
CNST11 1.0000000
D1 2.00000000 sec
D20 0.00689655 sec
TD0 1

======== CHANNEL f1 ========
SFO1 125.7459782 MHz
NUC1 13C
P1 9.00 usec
P2 18.00 usec
PLW1 140.00000000 W

======== CHANNEL f2 ========
SFO2 500.0350280 MHz
NUC2 1H
CPDPRG[2 waltz16
PCPD2 80.00 usec
PLW2 14.50000000 W
PLW12 0.32624999 W

F2 - Processing parameters
SI 32768
SF 125.7334082 MHz
WDW EM
SSB 0
LB 1.00 Hz
GB 0
PC 1.40

^{1}H NMR of 3-Chloro-5-((3-hydroxyphenyl)amino)-4*H*-1,2,6-thiadiazin-4-one (**8**)

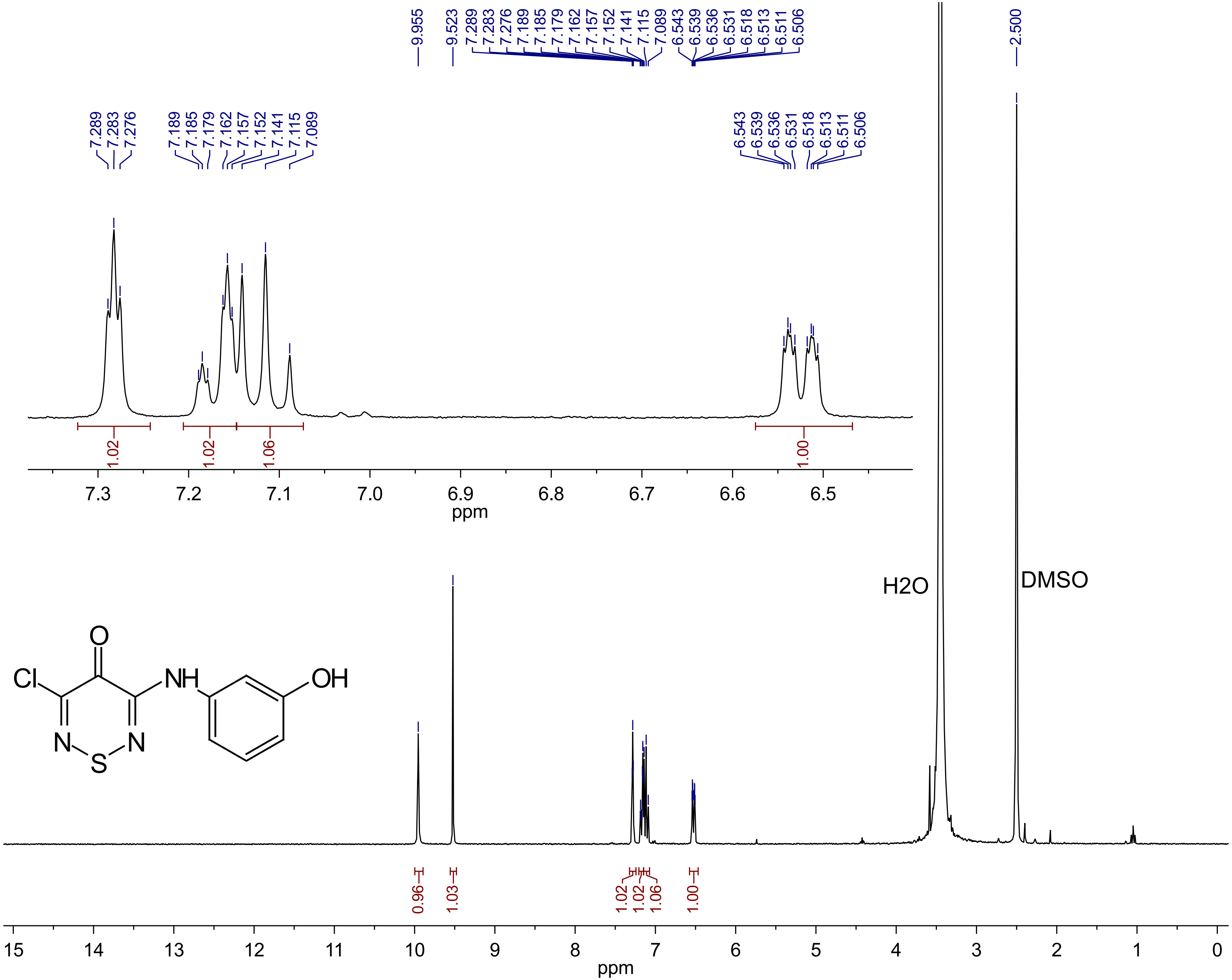

Current Data Parameters	
NAME	Andreas
EXPNO	247
PROCNO	1
F2 - Acquisition Parameters	
Date_	20201202
Time	12.04 h
INSTRUM	spect
PROBHD	Z104275_0375 (
PULPROG	zg30
TD	65536
SOLVENT	DMSO
NS	16
DS	2
SWH	6009.615 Hz
FIDRES	0.183399 Hz
AQ	5.4525952 sec
RG	129.23
DW	83.200 usec
DE	6.50 usec
TE	294.3 K
D1	1.00000000 sec
TD0	1
SFO1	300.1318533 MHz
NUC1	1H
P1	14.00 usec
PLW1	8.19999981 W
F2 - Processing parameters	
SI	65536
SF	300.1300024 MHz
WDW	EM
SSB	0
LB	0.30 Hz
GB	0
PC	1.00

^{13}C NMR of 3-Chloro-5-((3-hydroxyphenyl)amino)-4*H*-1,2,6-thiadiazin-4-one (**8**)

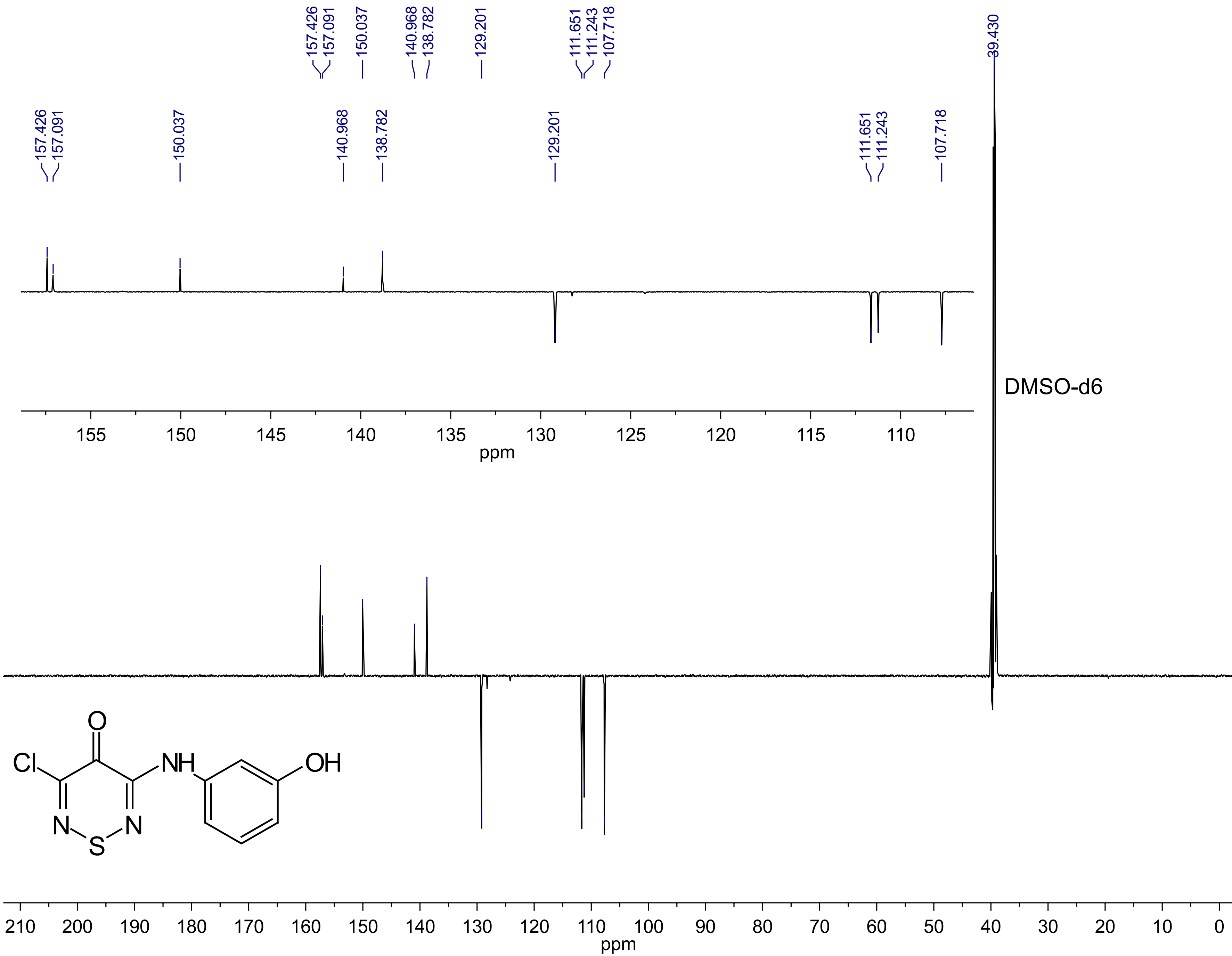

Current Data Parameters
NAME Kalogirou
EXPNO 470
PROCNO 1
F2 - Acquisition Parameters
Date_ 20171207
Time 9.32
INSTRUM spect
PROBHD 5 mm PABBO BB-
PULPROG jmod
TD 65536
SOLVENT DMSO
NS 12288
DS 4
SWH 29761.904 Hz
FIDRES 0.454131 Hz
AQ 1.1010048 sec
RG 2050
DW 16.800 usec
DE 6.50 usec
TE 296.6 K
CNST2 145.0000000
CNST11 1.0000000
D1 2.00000000 sec
D20 0.00689655 sec
TD0 1
======== CHANNEL f1 ========
SFO1 125.7459782 MHz
NUC1 13C
P1 9.00 usec
P2 18.00 usec
PLW1 140.00000000 W
======== CHANNEL f2 ========
SFO2 500.0350280 MHz
NUC2 1H
CPDPRG[2 waltz16
PCPD2 80.00 usec
PLW2 14.50000000 W
PLW12 0.32624999 W
F2 - Processing parameters
SI 32768
SF 125.7334742 MHz
WDW EM
SSB 0
LB 1.00 Hz
GB 0
PC 1.40

^{1}H NMR of 3-Chloro-5-((5-hydroxy-2-methylphenyl)amino)-4*H*-1,2,6-thiadiazin-4-one (**10**)

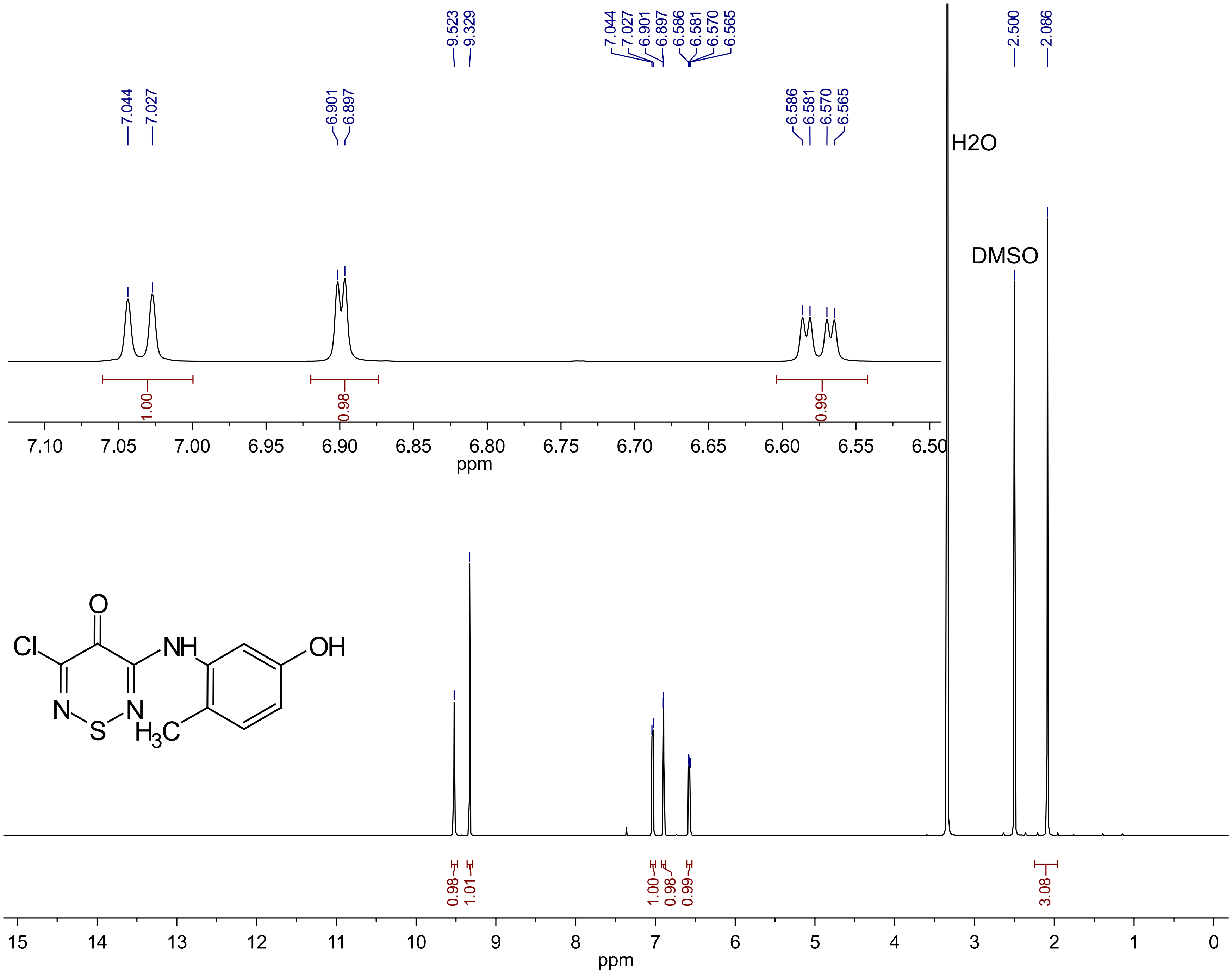

Current Data Parameters
NAME Kalogirou
EXPNO 474
PROCNO 1
F2 - Acquisition Parameters
Date_ 20171212
Time 19.56
INSTRUM spect
PROBHD 5 mm PABBO BB-
PULPROG zg30
TD 65536
SOLVENT DMSO
NS 16
DS 2
SWH 10000.000 Hz
FIDRES 0.152588 Hz
AQ 3.2767999 sec
RG 144
DW 50.000 usec
DE 6.50 usec
TE 295.0 K
D1 1.00000000 sec
TD0 1
======== CHANNEL f1 ========
SFO1 500.0361158 MHz
NUC1 1H
P1 12.00 usec
PLW1 14.50000000 W
F2 - Processing parameters
SI 65536
SF 500.0330315 MHz
WDW EM
SSB 0
LB 0.30 Hz
GB 0
PC 1.00

^{13}C NMR of 3-Chloro-5-((5-hydroxy-2-methylphenyl)amino)-4*H*-1,2,6-thiadiazin-4-one (**10**)

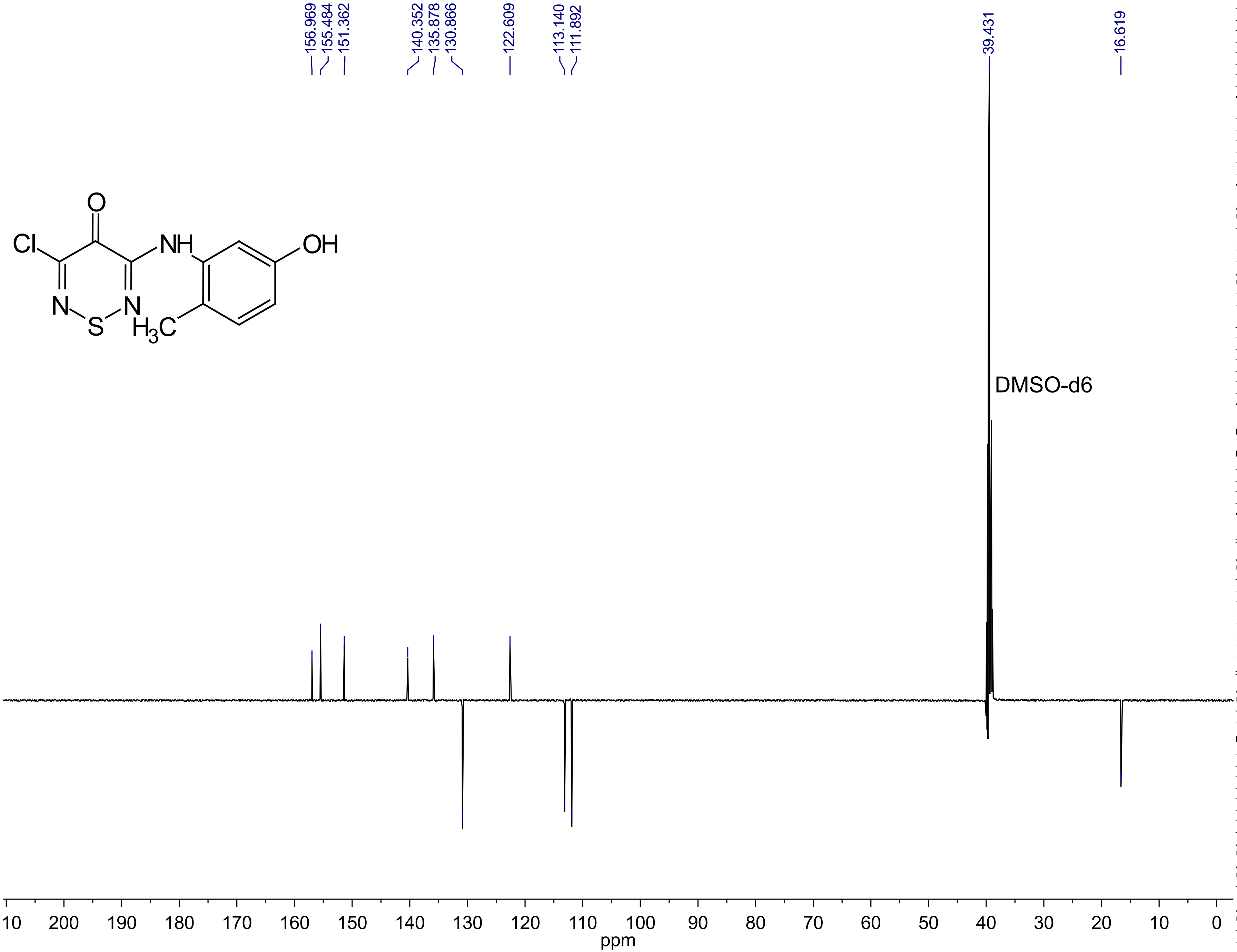

Current Data Parameters
NAME Kalogirou
EXPNO 475
PROCNO 1
F2 - Acquisition Parameters
Date_ 20171213
Time 9.24
INSTRUM spect
PROBHD 5 mm PABBO BB-
PULPROG jmod
TD 65536
SOLVENT DMSO
NS 15360
DS 4
SWH 29761.904 Hz
FIDRES 0.454131 Hz
AQ 1.1010048 sec
RG 2050
DW 16.800 usec
DE 6.50 usec
TE 296.3 K
CNST2 145.0000000
CNST11 1.0000000
D1 2.00000000 sec
D20 0.00689655 sec
TD0 1
======== CHANNEL f1 ========
SFO1 125.7459782 MHz
NUC1 13C
P1 9.00 usec
P2 18.00 usec
PLW1 140.00000000 W
======== CHANNEL f2 ========
SFO2 500.0350280 MHz
NUC2 1H
CPDPRG[2 waltz16
PCPD2 80.00 usec
PLW2 14.50000000 W
PLW12 0.32624999 W
F2 - Processing parameters
SI 32768
SF 125.7334738 MHz
WDW EM
SSB 0
LB 1.00 Hz
GB 0
PC 1.40

^{1}H NMR of 3-Chloro-5-(4-methylpiperazin-1-yl)-4*H*-1,2,6-thiadiazin-4-one (**12**)

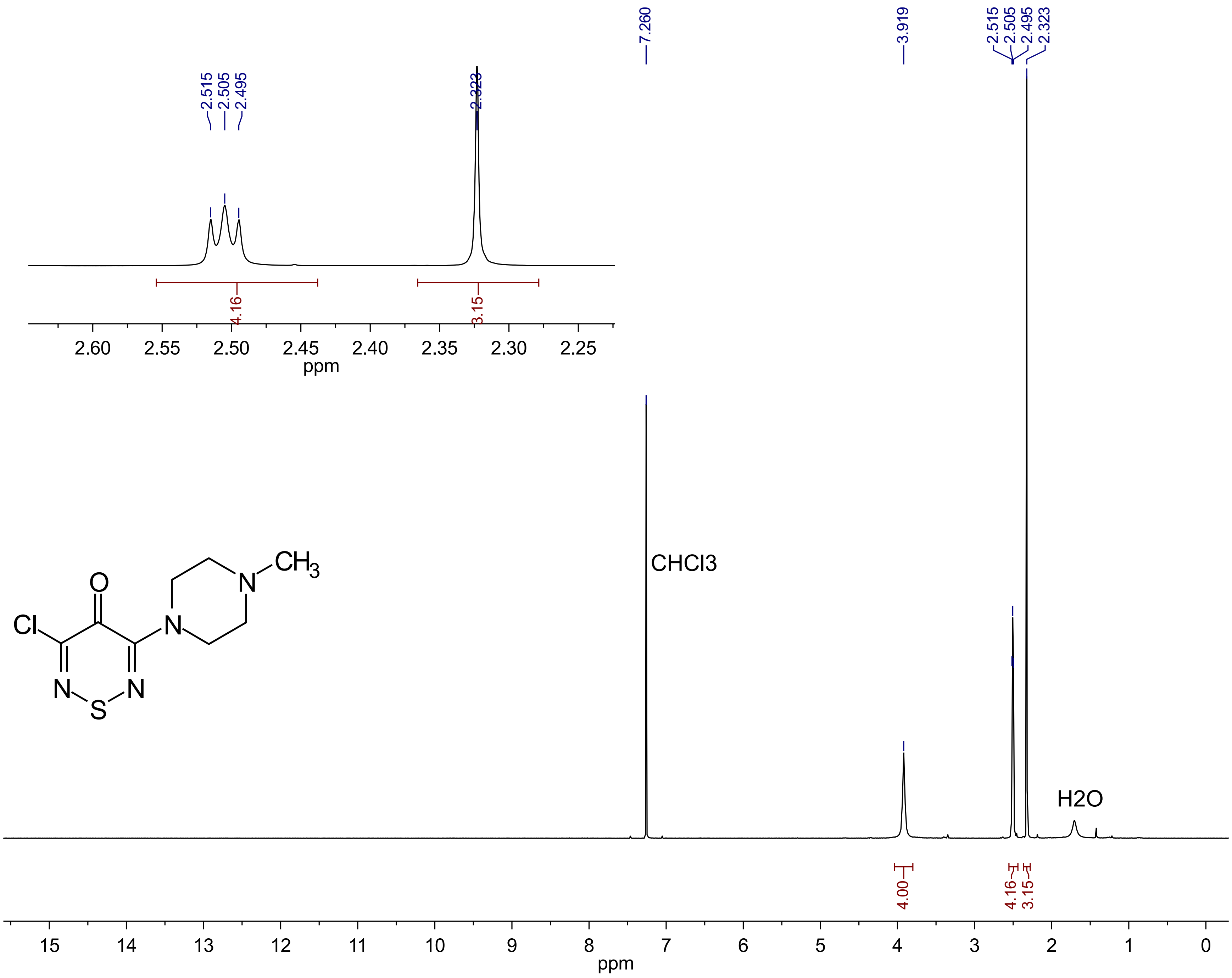

Current Data Parameters
NAME Kalogirou
EXPNO 431
PROCNO 1
F2 - Acquisition Parameters
Date_ 20171015
Time 9.04
INSTRUM spect
PROBHD 5 mm PABBO BB-
PULPROG zg30
TD 65536
SOLVENT CDCl3
NS 16
DS 2
SWH 10000.000 Hz
FIDRES 0.152588 Hz
AQ 3.2767999 sec
RG 161
DW 50.000 usec
DE 6.50 usec
TE 295.2 K
D1 1.00000000 sec
TD0 1
CHANNEL f1
SFO1 500.0361158 MHz
NUC1 1H
P1 12.00 usec
PLW1 14.50000000 W

F2 - Processing parameters
SI 65536
SF 500.0330403 MHz
WDW EM
SSB 0
LB 0.30 Hz
GB 0
PC 1.00

^{13}C NMR of 3-Chloro-5-(4-methylpiperazin-1-yl)-4*H*-1,2,6-thiadiazin-4-one (**12**)

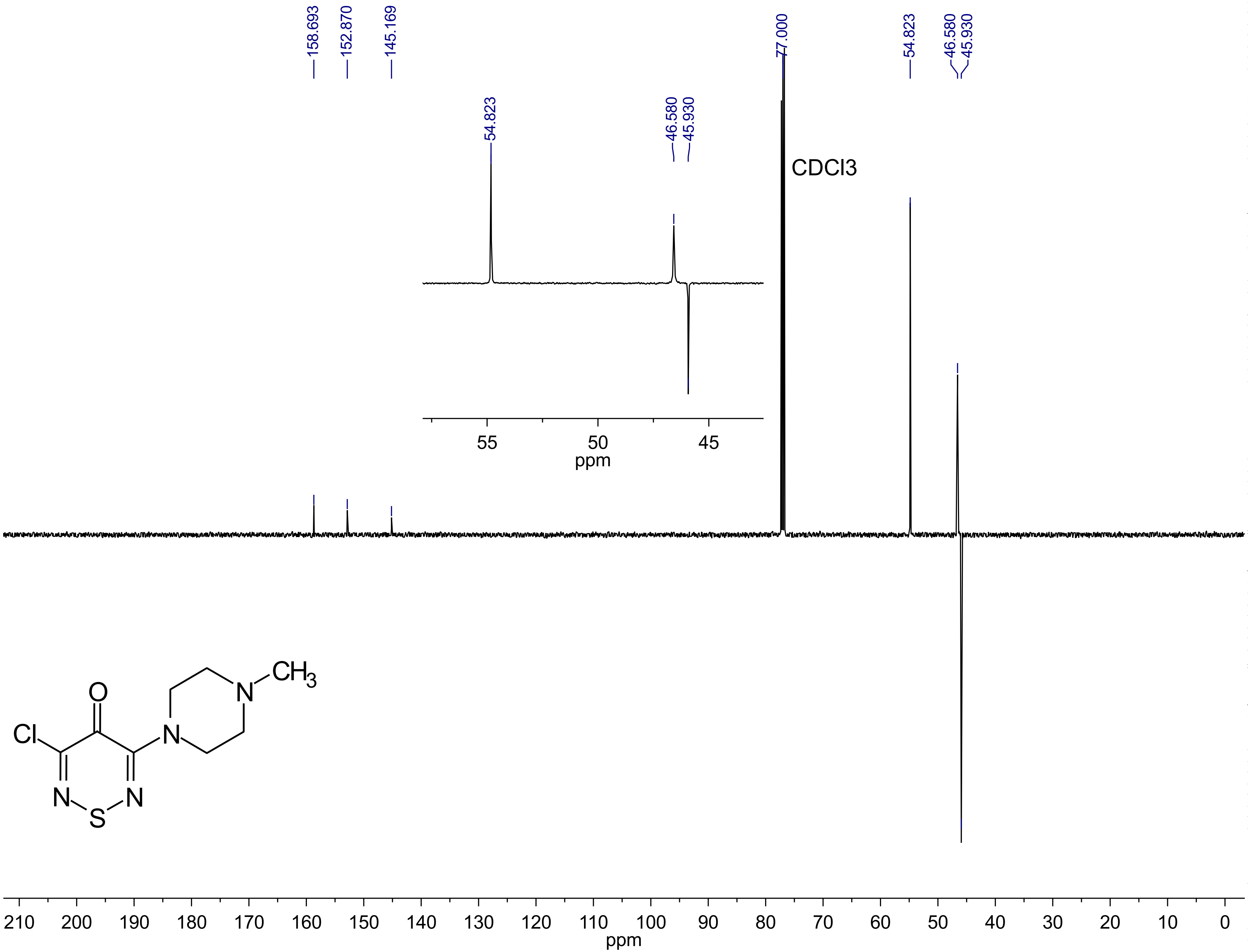

Current Data Parameters
NAME Kalogirou
EXPNO 432
PROCNO 1
F2 - Acquisition Parameters
Date_ 20171015
Time 17.16
INSTRUM spect
PROBHD 5 mm PABBO BB-
PULPROG jmod
TD 65536
SOLVENT CDCl3
NS 9216
DS 4
SWH 29761.904 Hz
FIDRES 0.454131 Hz
AQ 1.1010048 sec
RG 2050
DW 16.800 usec
DE 6.50 usec
TE 295.6 K
CNST2 145.0000000
CNST11 1.0000000
D1 2.00000000 sec
D20 0.00689655 sec
TD0 1
======== CHANNEL f1 ========
SFO1 125.7459782 MHz
NUC1 13C
P1 9.00 usec
P2 18.00 usec
PLW1 140.00000000 W
======== CHANNEL f2 ========
SFO2 500.0350280 MHz
NUC2 1H
CPDPRG[2 waltz16
PCPD2 80.00 usec
PLW2 14.50000000 W
PLW12 0.32624999 W
F2 - Processing parameters
SI 32768
SF 125.7334087 MHz
WDW EM
SSB 0
LB 1.00 Hz
GB 0
PC 1.40

^{1}H NMR of 3-((1*H*-indazol-5-yl)amino)-5-chloro-4*H*-1,2,6-thiadiazin-4-one (**13**)

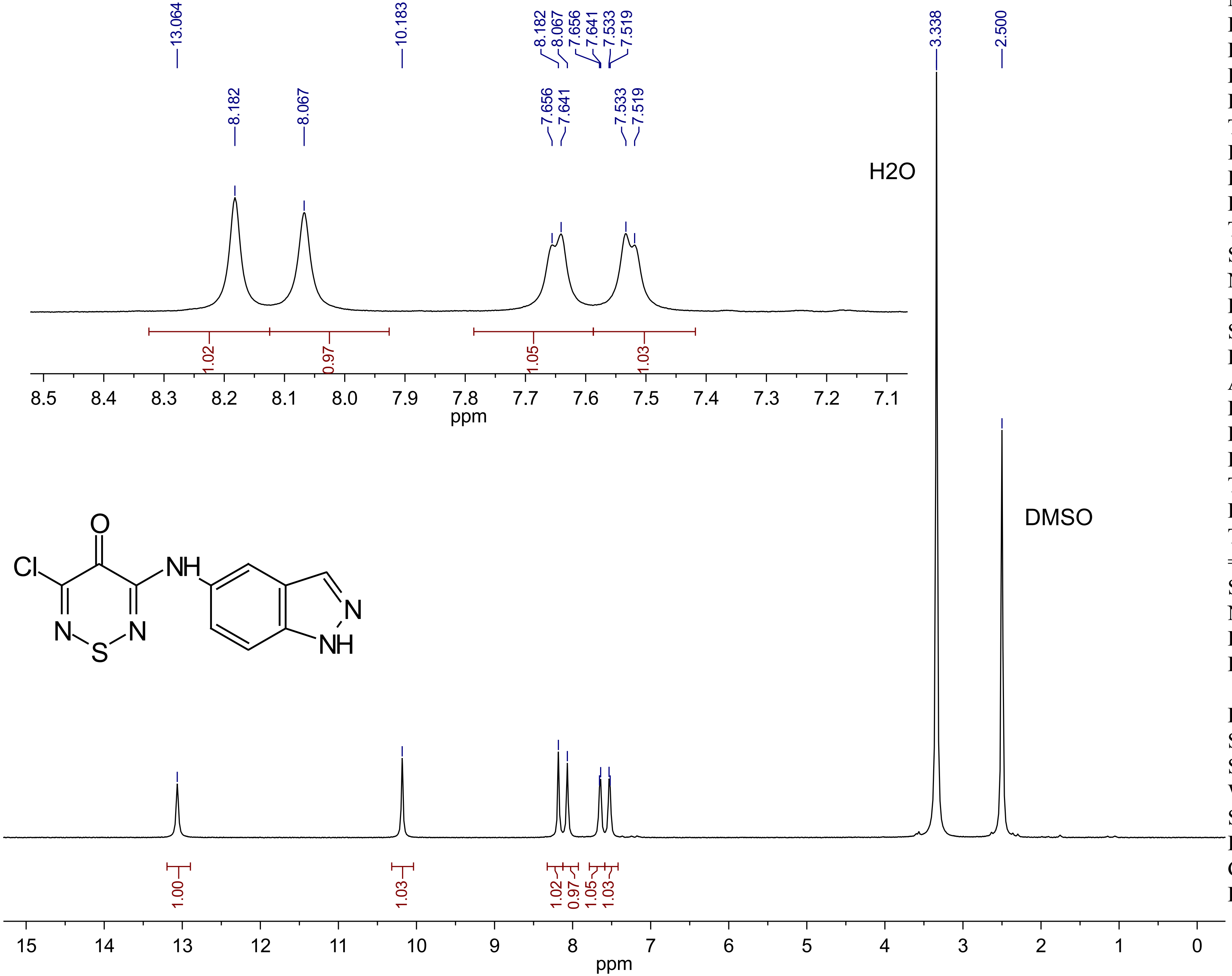

Current Data Parameters
NAME Kalogirou
EXPNO 457
PROCNO 1
F2 - Acquisition Parameters
Date_ 20171128
Time 20.02
INSTRUM spect
PROBHD 5 mm PABBO BB-
PULPROG zg30
TD 65536
SOLVENT DMSO
NS 16
DS 2
SWH 10000.000 Hz
FIDRES 0.152588 Hz
AQ 3.2767999 sec
RG 144
DW 50.000 usec
DE 6.50 usec
TE 296.3 K
D1 1.00000000 sec
TD0 1
======== CHANNEL f1 ========
SFO1 500.0361158 MHz
NUC1 1H
P1 12.00 usec
PLW1 14.50000000 W

F2 - Processing parameters
SI 65536
SF 500.0330310 MHz
WDW EM
SSB 0
LB 0.30 Hz
GB 0
PC 1.00

^{13}C NMR of 3-((1*H*-indazol-5-yl)amino)-5-chloro-4*H*-1,2,6-thiadiazin-4-one (**13**)

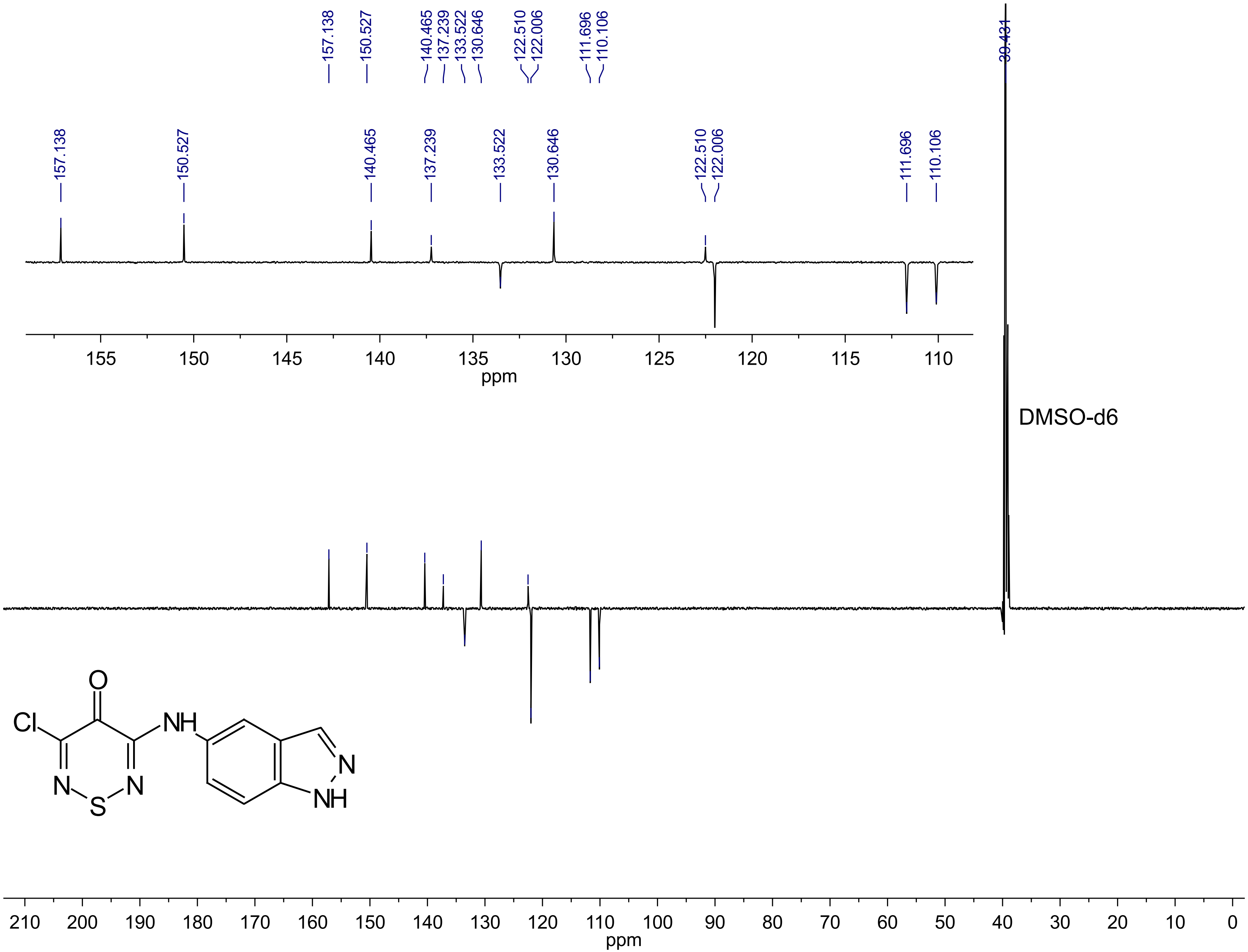

Current Data Parameters
NAME Kalogirou
EXPNO 458
PROCNO 1
F2 - Acquisition Parameters
Date_ 20171129
Time 9.03
INSTRUM spect
PROBHD 5 mm PABBO BB-
PULPROG jmod
TD 65536
SOLVENT DMSO
NS 14848
DS 4
SWH 29761.904 Hz
FIDRES 0.454131 Hz
AQ 1.1010048 sec
RG 2050
DW 16.800 usec
DE 6.50 usec
TE 297.1 K
CNST2 145.0000000
CNST11 1.0000000
D1 2.00000000 sec
D20 0.00689655 sec
TD0 1
CHANNEL f1
SFO1 125.7459782 MHz
NUC1 13C
P1 9.00 usec
P2 18.00 usec
PLW1 140.00000000 W
CHANNEL f2
SFO2 500.0350280 MHz
NUC2 1H
CPDPRG[2 waltz16
PCPD2 80.00 usec
PLW2 14.50000000 W
PLW12 0.32624999 W
F2 - Processing parameters
SI 32768
SF 125.7334752 MHz
WDW EM
SSB 0
LB 1.00 Hz
GB 0
PC 1.40

^{1}H NMR of 3-((3-Acetylphenyl)amino)-5-(1*H*-pyrrolo[2,3-*b*]pyridin-4-yl)-4*H*-1,2,6-thiadiazin-4-one (**14**)

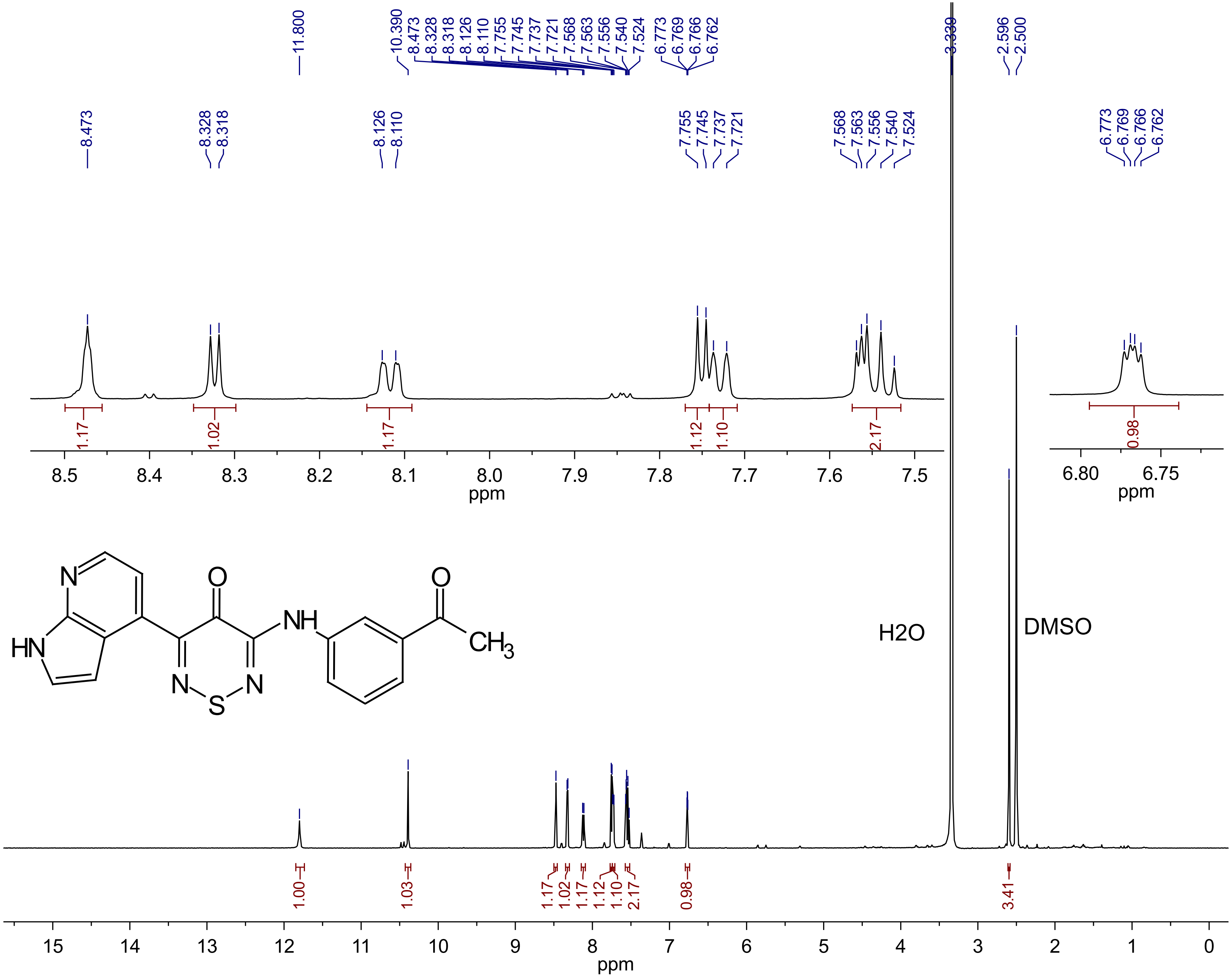

Current Data Parameters
NAME Kalogirou
EXPNO 554
PROCNO 1
F2 - Acquisition Parameters
Date_ 20180824
Time 14.24
INSTRUM spect
PROBHD 5 mm PABBO BB-
PULPROG zg30
TD 65536
SOLVENT DMSO
NS 16
DS 2
SWH 10000.000 Hz
FIDRES 0.152588 Hz
AQ 3.2767999 sec
RG 114
DW 50.000 usec
DE 6.50 usec
TE 298.9 K
D1 1.00000000 sec
TD0 1
======== CHANNEL f1 ========
SFO1 500.0361158 MHz
NUC1 1H
P1 12.00 usec
PLW1 14.80000019 W

F2 - Processing parameters
SI 65536
SF 500.0330327 MHz
WDW EM
SSB 0
LB 0.30 Hz
GB 0
PC 1.00

^{13}C NMR of 3-((3-Acetylphenyl)amino)-5-(1*H*-pyrrolo[2,3-*b*]pyridin-4-yl)-4*H*-1,2,6-thiadiazin-4-one (**14**)

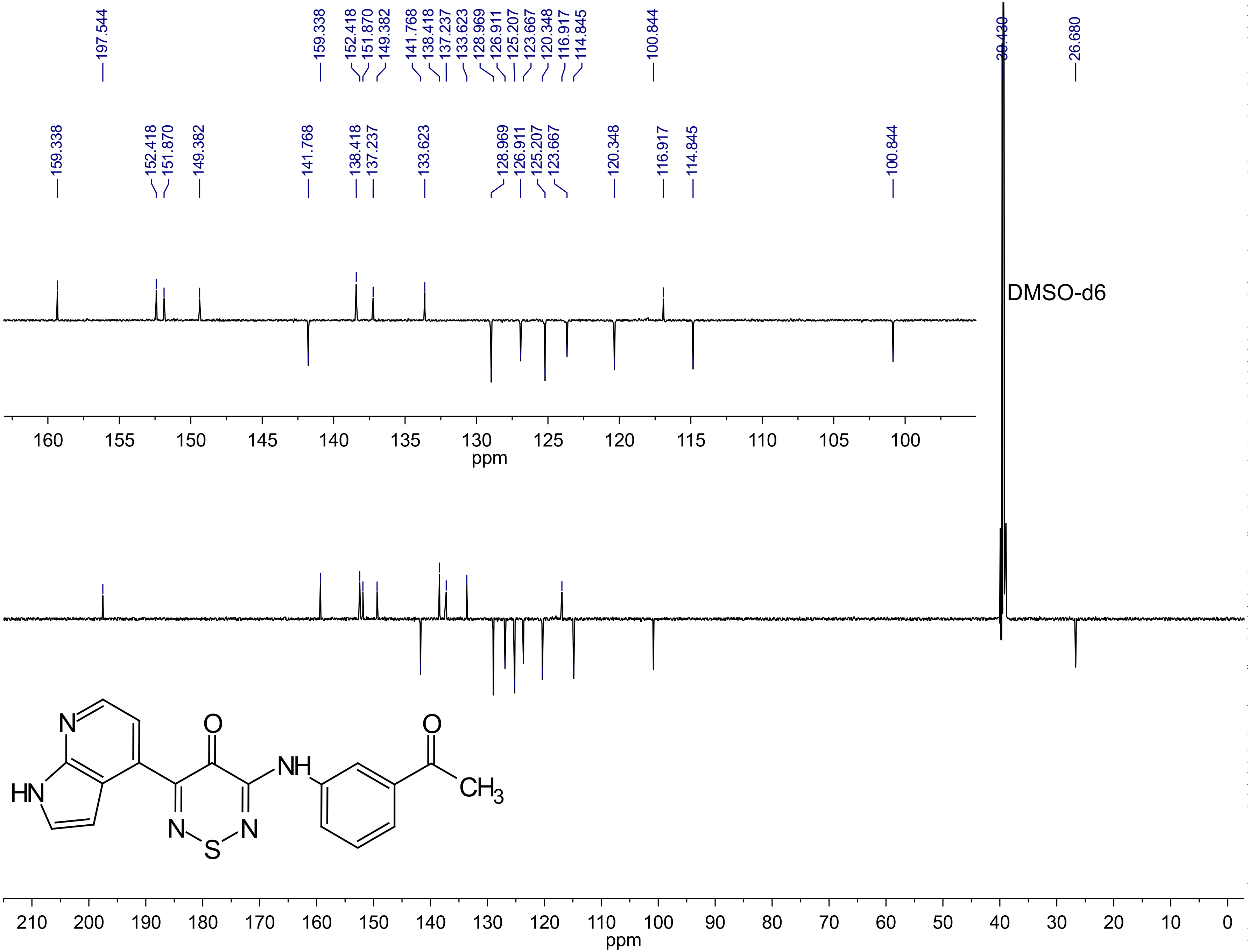

Current Data Parameters
NAME Kalogirou
EXPNO 553
PROCNO 1
F2 - Acquisition Parameters
Date_ 20180824
Time 8.26
INSTRUM spect
PROBHD 5 mm PABBO BB-
PULPROG jmod
TD 65536
SOLVENT DMSO
NS 16384
DS 4
SWH 29761.904 Hz
FIDRES 0.454131 Hz
AQ 1.1010048 sec
RG 2050
DW 16.800 usec
DE 6.50 usec
TE 302.1 K
CNST2 145.0000000
CNST11 1.0000000
D1 2.00000000 sec
D20 0.00689655 sec
TD0 1
======== CHANNEL f1 ========
SFO1 125.7459782 MHz
NUC1 13C
P1 9.40 usec
P2 18.80 usec
PLW1 140.00000000 W
======== CHANNEL f2 ========
SFO2 500.0350280 MHz
NUC2 1H
CPDPRG[2 waltz16
PCPD2 80.00 usec
PLW2 14.80000019 W
PLW12 0.33300000 W
F2 - Processing parameters
SI 32768
SF 125.7334792 MHz
WDW EM
SSB 0
LB 1.00 Hz
GB 0
PC 1.40

^{1}H NMR of 3-((3-Methoxyphenyl)amino)-5-(1*H*-pyrrolo[2,3-*b*]pyridin-4-yl)-4*H*-1,2,6-thiadiazin-4-one (**15**)

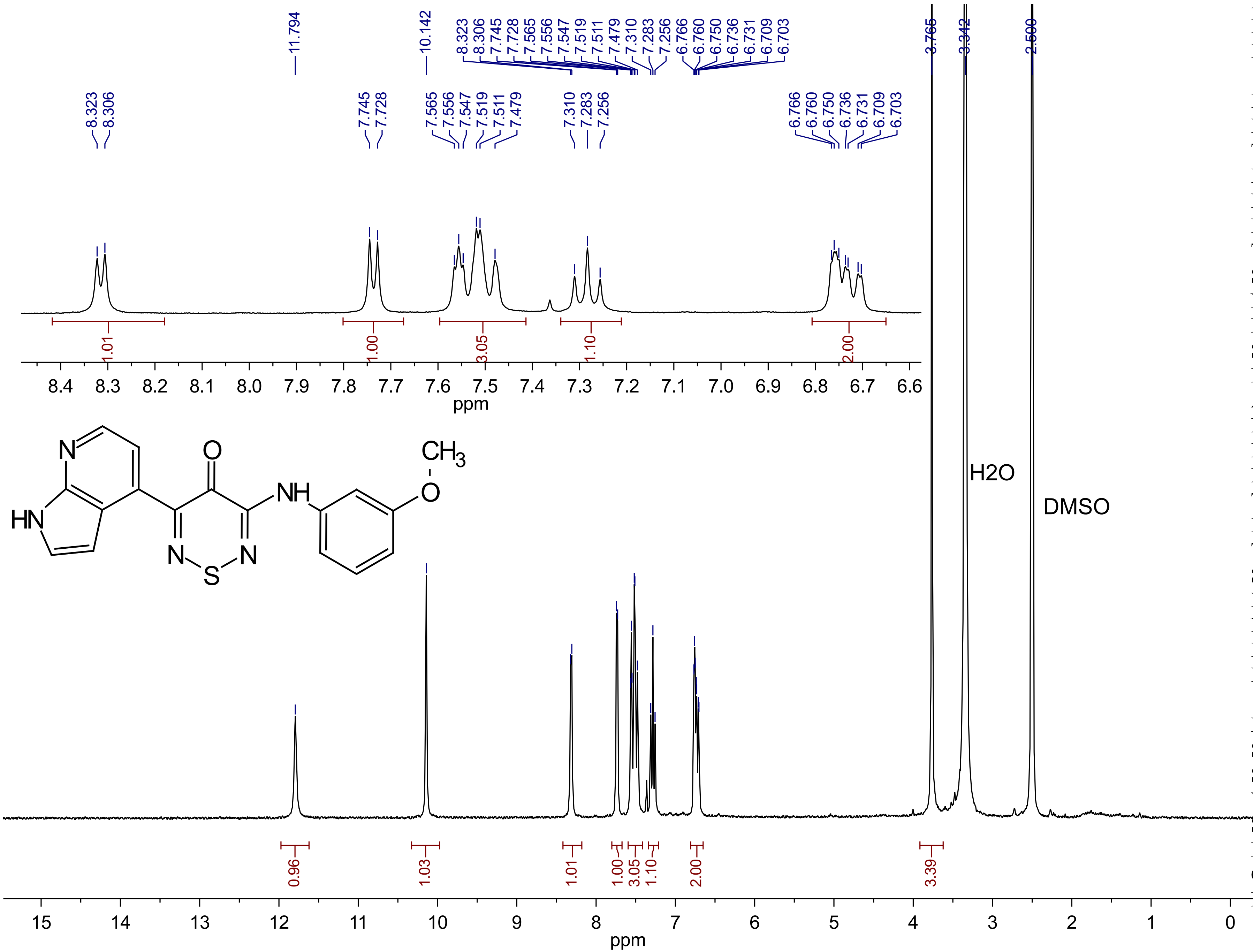

Current Data Parameters
NAME Andreas
EXPNO 132
PROCNO 1

F2 - Acquisition Parameters
Date_ 20180813
Time 12.44 h
INSTRUM spect
PROBHD Z104275_0375 (
PULPROG zg30
TD 65536
SOLVENT DMSO
NS 16
DS 2
SWH 6009.615 Hz
FIDRES 0.183399 Hz
AQ 5.4525952 sec
RG 201.81
DW 83.200 usec
DE 6.50 usec
TE 298.0 K
D1 1.00000000 sec
TD0 1
SFO1 300.1318533 MHz
NUC1 1H
P1 14.00 usec
PLW1 7.50000000 W

F2 - Processing parameters
SI 65536
SF 300.1300026 MHz
WDW EM
SSB 0
LB 0.30 Hz
GB 0
PC 1.00

^{13}C NMR of 3-((3-Methoxyphenyl)amino)-5-(1*H*-pyrrolo[2,3-*b*]pyridin-4-yl)-4*H*-1,2,6-thiadiazin-4-one (**15**)

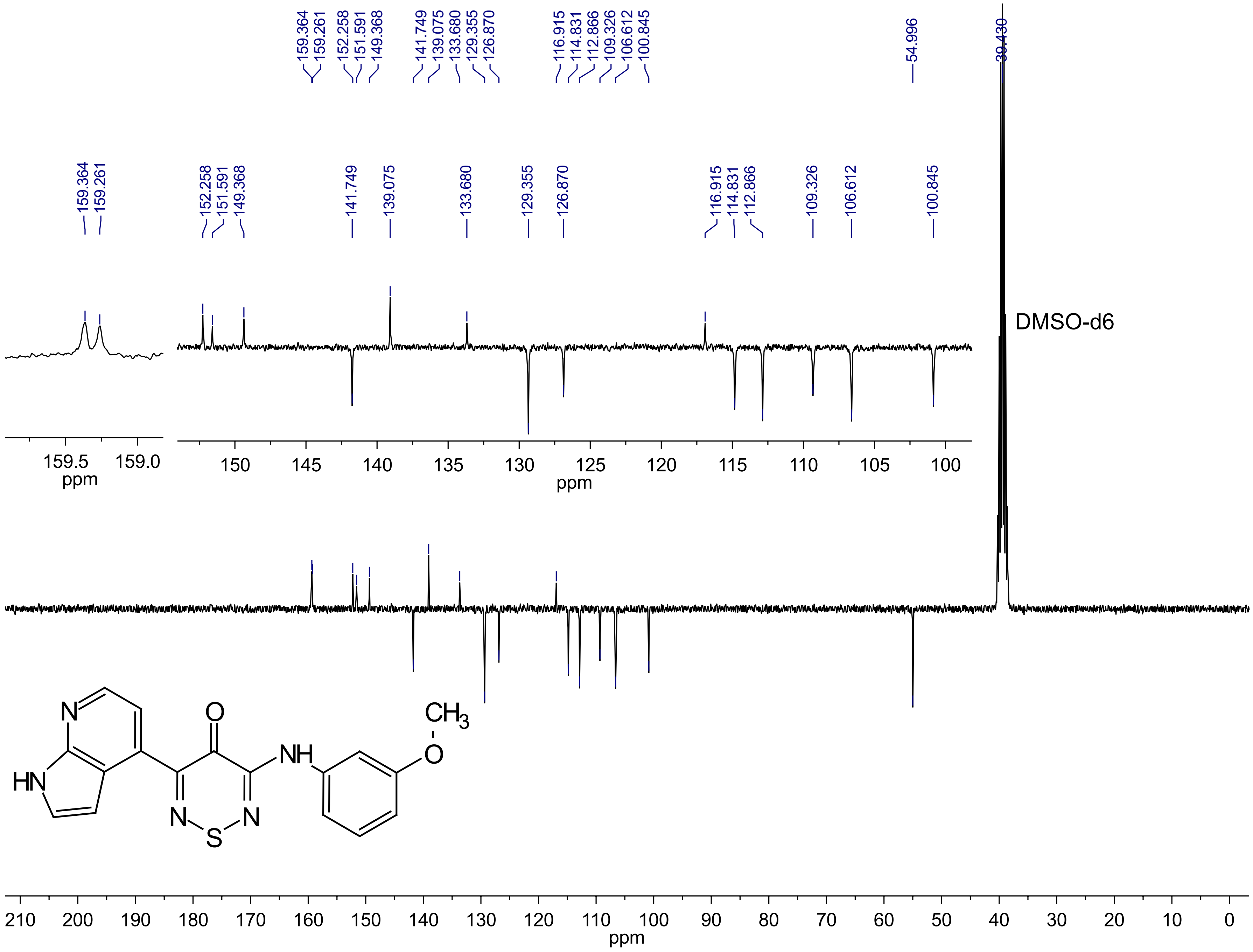

Current Data Parameters
NAME Andreas
EXPNO 133
PROCNO 1
F2 - Acquisition Parameters
Date_ 20180814
Time 8.11 h
INSTRUM spect
PROBHD Z104275_0375 (
PULPROG jmod
TD 65536
SOLVENT DMSO
NS 18000
DS 4
SWH 18115.941 Hz
FIDRES 0.552855 Hz
AQ 1.8087935 sec
RG 201.81
DW 27.600 usec
DE 6.50 usec
TE 298.4 K
CNST2 145.0000000
CNST11 1.0000000
D1 2.00000000 sec
D20 0.00689655 sec
TD0 1
SFO1 75.4752953 MHz
NUC1 13C
P1 10.00 usec
P2 20.00 usec
PLW1 40.05500031 W
SFO2 300.1312005 MHz
NUC2 1H
CPDPRG[2 waltz16
PCPD2 90.00 usec
PLW2 7.50000000 W
PLW12 0.18148001 W
F2 - Processing parameters
SI 32768
SF 75.4677922 MHz
WDW EM
SSB 0
LB 1.00 Hz
GB 0
PC 1.40

^{1}H NMR of 3-((3-Hydroxyphenyl)amino)-5-(1*H*-pyrrolo[2,3-*b*]pyridin-4-yl)-4*H*-1,2,6-thiadiazin-4-one (**16**)

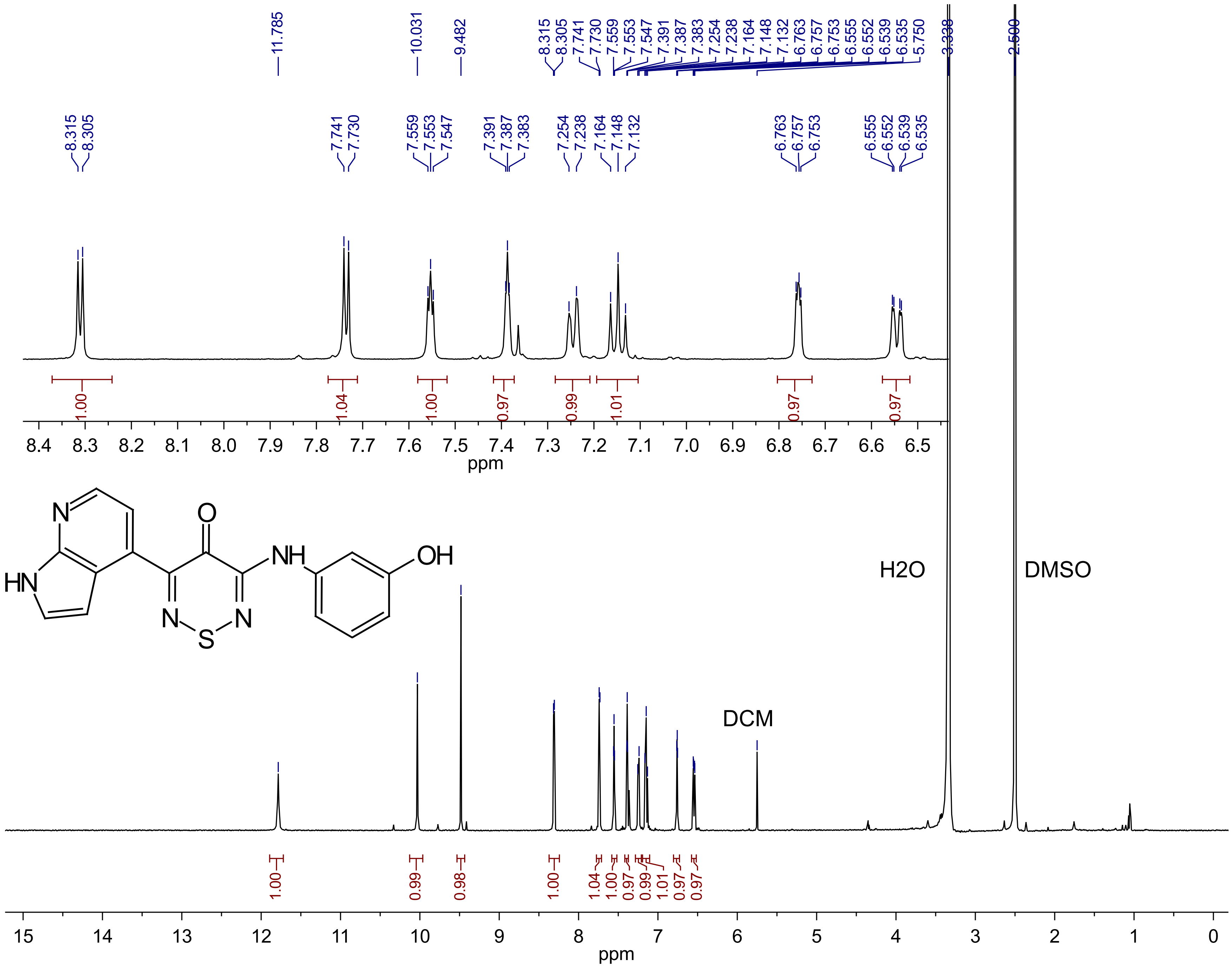

Current Data Parameters
NAME Kalogirou
EXPNO 555
PROCNO 1
F2 - Acquisition Parameters
Date_ 20180828
Time 17.59
INSTRUM spect
PROBHD 5 mm PABBO BB-
PULPROG zg30
TD 65536
SOLVENT DMSO
NS 16
DS 2
SWH 10000.000 Hz
FIDRES 0.152588 Hz
AQ 3.2767999 sec
RG 128
DW 50.000 usec
DE 6.50 usec
TE 299.0 K
D1 1.00000000 sec
TD0 1
CHANNEL f1
SFO1 500.0361158 MHz
NUC1 1H
P1 12.00 usec
PLW1 14.80000019 W
F2 - Processing parameters
SI 65536
SF 500.0330327 MHz
WDW EM
SSB 0
LB 0.30 Hz
GB 0
PC 1.00

^{13}C NMR of 3-((3-Hydroxyphenyl)amino)-5-(1*H*-pyrrolo[2,3-*b*]pyridin-4-yl)-4*H*-1,2,6-thiadiazin-4-one (**16**)

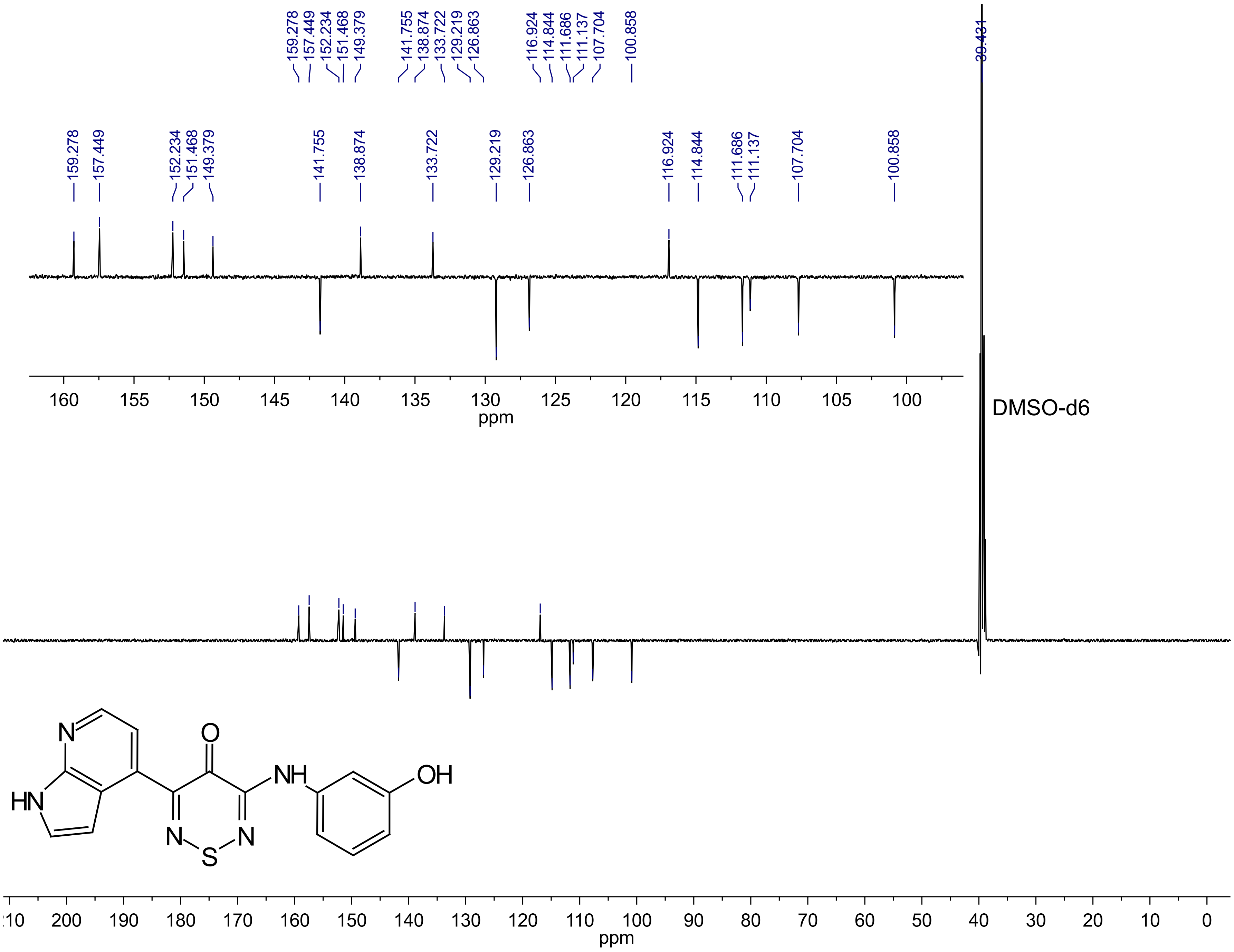

Current Data Parameters
NAME Kalogirou
EXPNO 556
PROCNO 1
F2 - Acquisition Parameters
Date_ 20180829
Time 9.14
INSTRUM spect
PROBHD 5 mm PABBO BB-
PULPROG jmod
TD 65536
SOLVENT DMSO
NS 17408
DS 4
SWH 29761.904 Hz
FIDRES 0.454131 Hz
AQ 1.1010048 sec
RG 1820
DW 16.800 usec
DE 6.50 usec
TE 301.0 K
CNST2 145.0000000
CNST11 1.0000000
D1 2.00000000 sec
D20 0.00689655 sec
TD0 1
======== CHANNEL f1 ========
SFO1 125.7459782 MHz
NUC1 13C
P1 9.40 usec
P2 18.80 usec
PLW1 140.00000000 W
======== CHANNEL f2 ========
SFO2 500.0350280 MHz
NUC2 1H
CPDPRG[2 waltz16
PCPD2 80.00 usec
PLW2 14.80000019 W
PLW12 0.33300000 W
F2 - Processing parameters
SI 32768
SF 125.7334776 MHz
WDW EM
SSB 0
LB 1.00 Hz
GB 0
PC 1.40

^{1}H NMR of 3-((3-Hydroxy-4-methylphenyl)amino)-5-(1*H*-pyrrolo[2,3-*b*]pyridin-4-yl)-4*H*-1,2,6-thiadiazin-4-one (**17**)

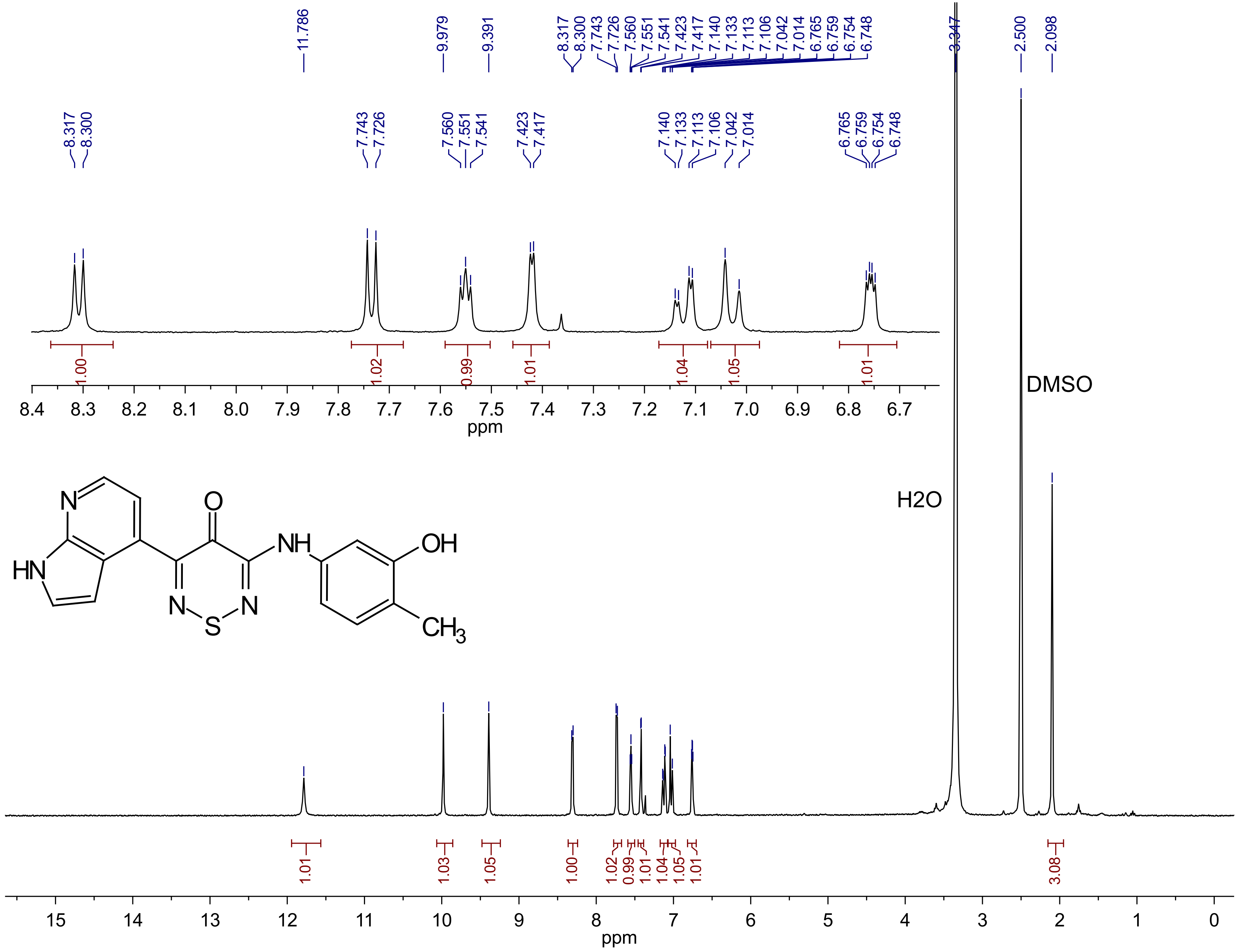

Current Data Parameters
NAME Andreas
EXPNO 136
PROCNO 1

F2 - Acquisition Parameters
Date_ 20180827
Time 17.49 h
INSTRUM spect
PROBHD Z104275_0375 (
PULPROG zg30
TD 65536
SOLVENT DMSO
NS 16
DS 2
SWH 6009.615 Hz
FIDRES 0.183399 Hz
AQ 5.4525952 sec
RG 201.81
DW 83.200 usec
DE 6.50 usec
TE 298.1 K
D1 1.00000000 sec
TD0 1
SFO1 300.1318533 MHz
NUC1 1H
P1 14.00 usec
PLW1 7.50000000 W

F2 - Processing parameters
SI 65536
SF 300.1300027 MHz
WDW EM
SSB 0
LB 0.30 Hz
GB 0
PC 1.00

^{13}C NMR of 3-((3-Hydroxy-4-methylphenyl)amino)-5-(1*H*-pyrrolo[2,3-*b*]pyridin-4-yl)-4*H*-1,2,6-thiadiazin-4-one (**17**)

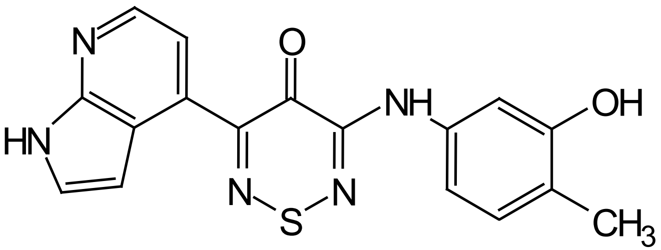

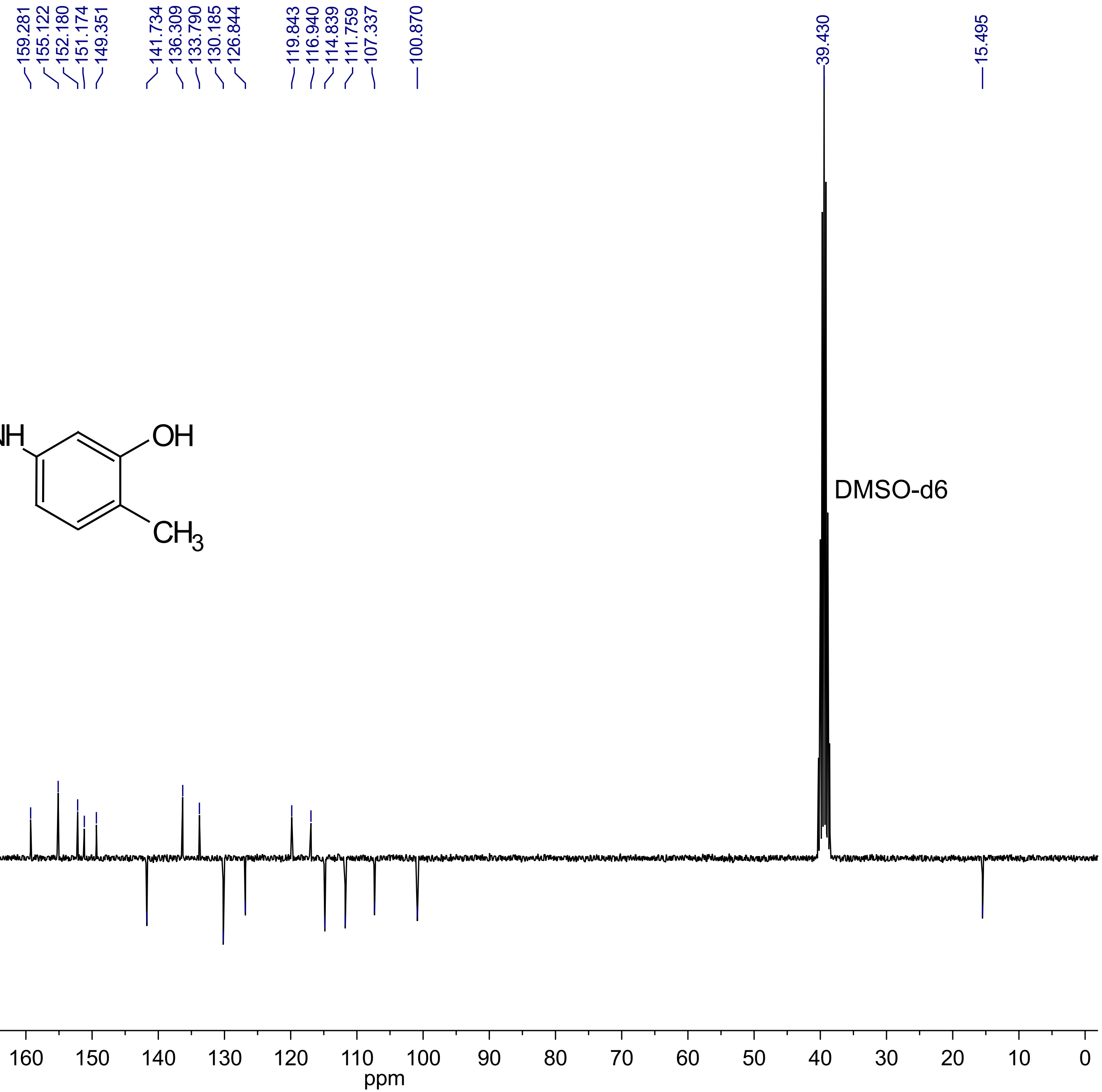

Current Data Parameters
NAME Andreas
EXPNO 137
PROCNO 1
F2 - Acquisition Parameters
Date_ 20180828
Time 12.58 h
INSTRUM spect
PROBHD Z104275_0375 (
PULPROG jmod
TD 65536
SOLVENT DMSO
NS 17794
DS 4
SWH 18115.941 Hz
FIDRES 0.552855 Hz
AQ 1.8087935 sec
RG 201.81
DW 27.600 usec
DE 6.50 usec
TE 298.6 K
CNST2 145.0000000
CNST11 1.0000000
D1 2.00000000 sec
D20 0.00689655 sec
TD0 1
SFO1 75.4752953 MHz
NUC1 13C
P1 10.00 usec
P2 20.00 usec
PLW1 40.05500031 W
SFO2 300.1312005 MHz
NUC2 1H
CPDPRG[2 waltz16
PCPD2 90.00 usec
PLW2 7.50000000 W
PLW12 0.18148001 W
F2 - Processing parameters
SI 32768
SF 75.4677920 MHz
WDW EM
SSB 0
LB 1.00 Hz
GB 0
PC 1.40

^{1}H NMR of 3-((5-Hydroxy-2-methylphenyl)amino)-5-(1*H*-pyrrolo[2,3-*b*]pyridin-4-yl)-4*H*-1,2,6-thiadiazin-4-one (**18**)

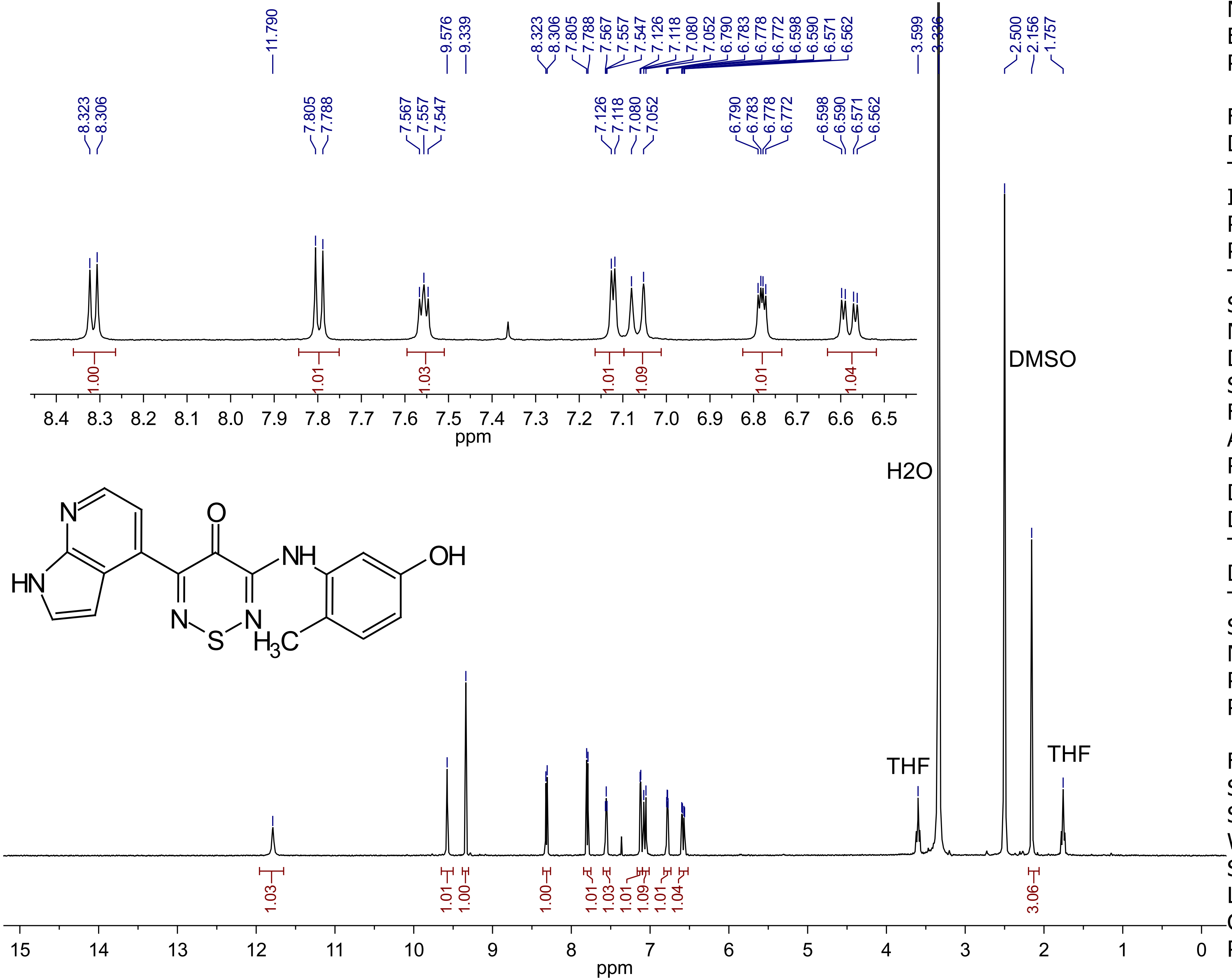

Current Data Parameters

NAME	Andreas
EXPNO	134
PROCNO	1

F2 - Acquisition Parameters

Date_	20180824
Time	18.44 h
INSTRUM	spect
PROBHD	Z104275_0375 (
PULPROG	zg30
TD	65536
SOLVENT	DMSO
NS	16
DS	2
SWH	6009.615 Hz
FIDRES	0.183399 Hz
AQ	5.4525952 sec
RG	201.81
DW	83.200 usec
DE	6.50 usec
TE	299.3 K
D1	1.00000000 sec
TD0	1
SFO1	300.1318533 MHz
NUC1	1H
P1	14.00 usec
PLW1	7.50000000 W

F2 - Processing parameters

SI	65536
SF	300.1300028 MHz
WDW	EM
SSB	0
LB	0.30 Hz
GB	0
PC	1.00

^{13}C NMR of 3-((5-Hydroxy-2-methylphenyl)amino)-5-(1*H*-pyrrolo[2,3-*b*]pyridin-4-yl)-4*H*-1,2,6-thiadiazin-4-one (**18**)

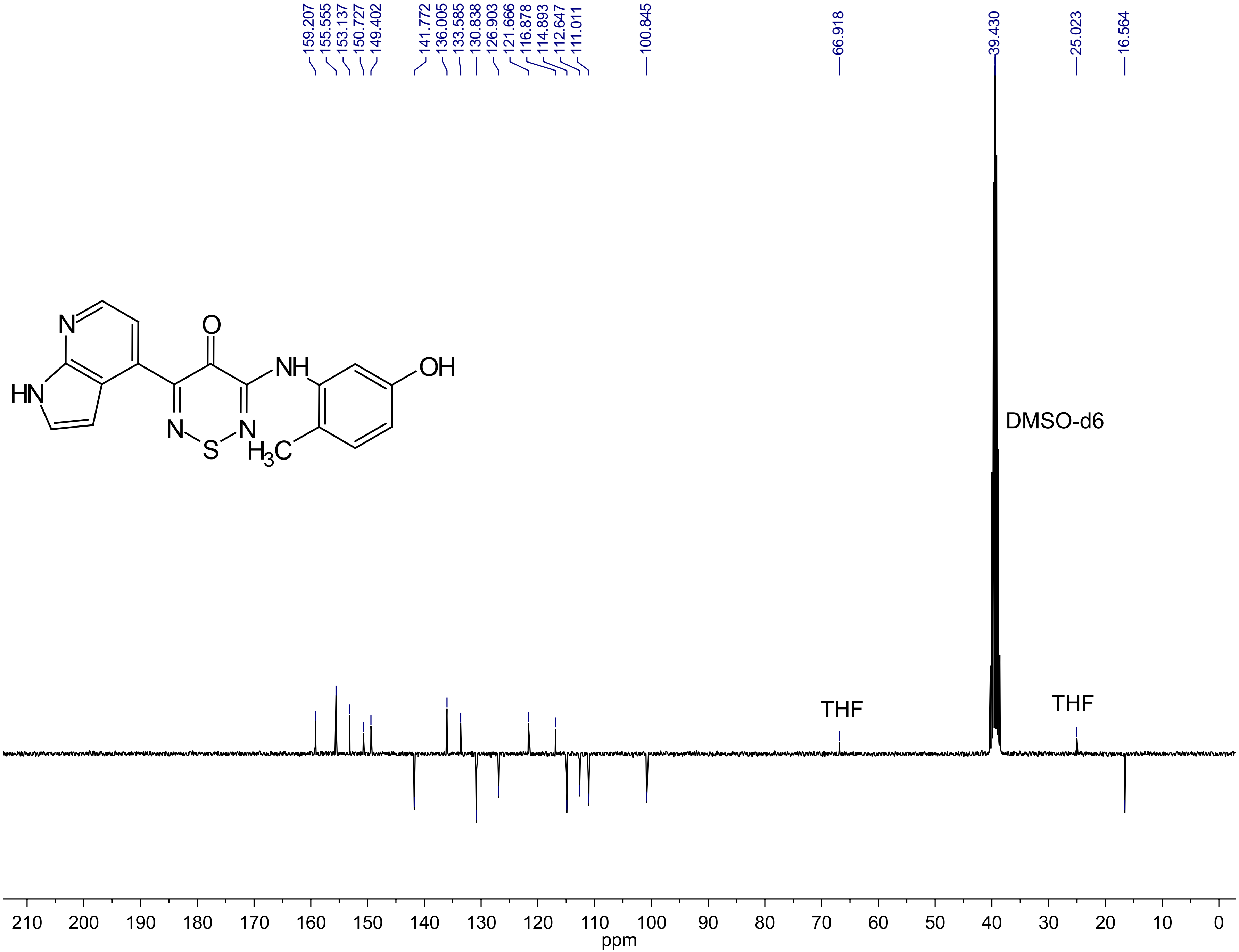

Current Data Parameters
NAME Andreas
EXPNO 135
PROCNO 1
F2 - Acquisition Parameters
Date_ 20180825
Time 20.33 h
INSTRUM spect
PROBHD Z104275_0375 (
PULPROG jmod
TD 65536
SOLVENT DMSO
NS 24000
DS 4
SWH 18115.941 Hz
FIDRES 0.552855 Hz
AQ 1.8087935 sec
RG 201.81
DW 27.600 usec
DE 6.50 usec
TE 299.6 K
CNST2 145.0000000
CNST11 1.0000000
D1 2.00000000 sec
D20 0.00689655 sec
TD0 1
SFO1 75.4752953 MHz
NUC1 13C
P1 10.00 usec
P2 20.00 usec
PLW1 40.05500031 W
SFO2 300.1312005 MHz
NUC2 1H
CPDPRG[2 waltz16
PCPD2 90.00 usec
PLW2 7.50000000 W
PLW12 0.18148001 W
F2 - Processing parameters
SI 32768
SF 75.4677922 MHz
WDW EM
SSB 0
LB 1.00 Hz
GB 0
PC 1.40

^{1}H NMR of 3-Morpholino-5-(1*H*-pyrrolo[2,3-*b*]pyridin-4-yl)-4*H*-1,2,6-thiadiazin-4-one (**19**)

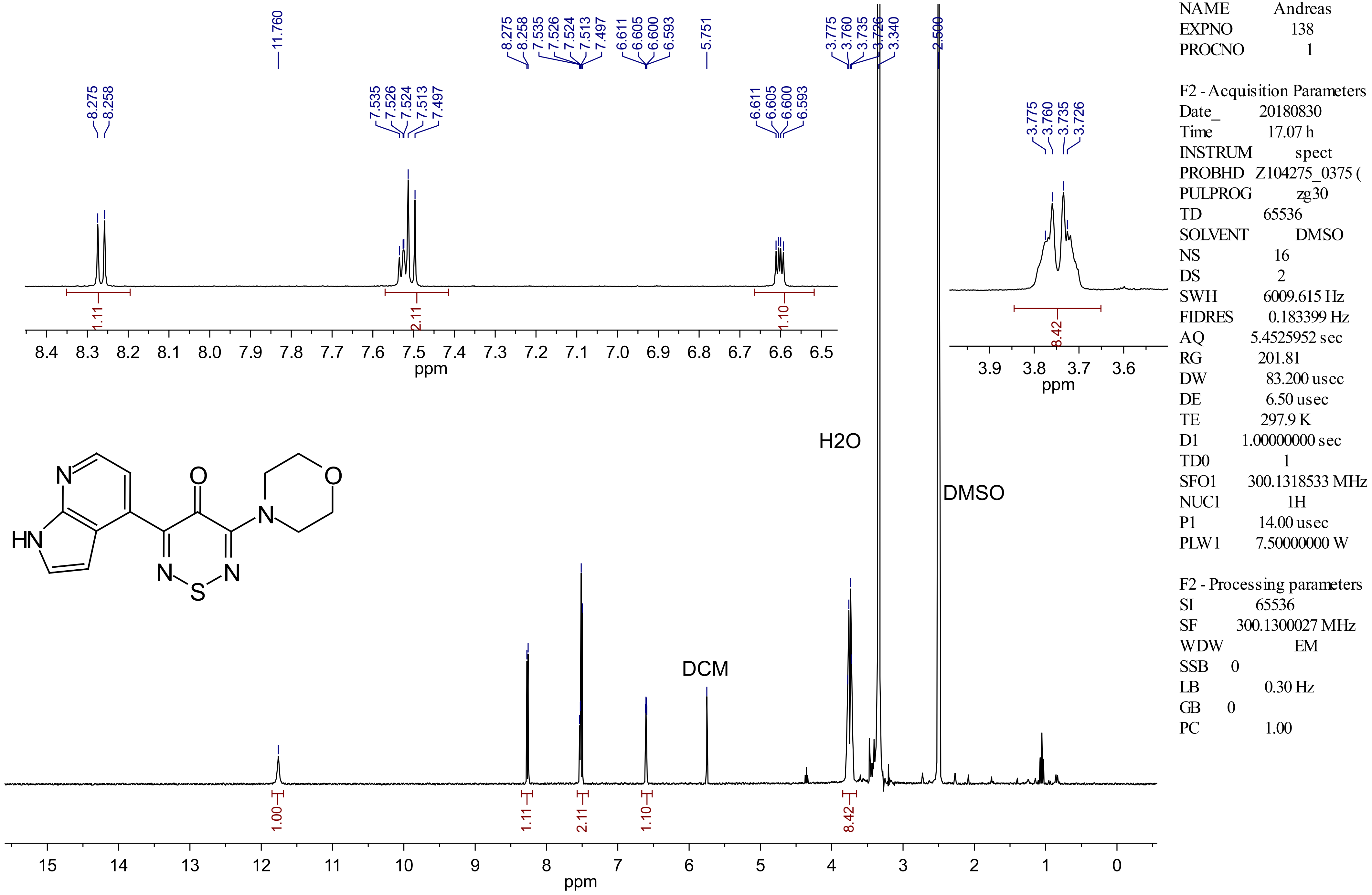

^{13}C NMR of 3-Morpholino-5-(1*H*-pyrrolo[2,3-*b*]pyridin-4-yl)-4*H*-1,2,6-thiadiazin-4-one (**19**)

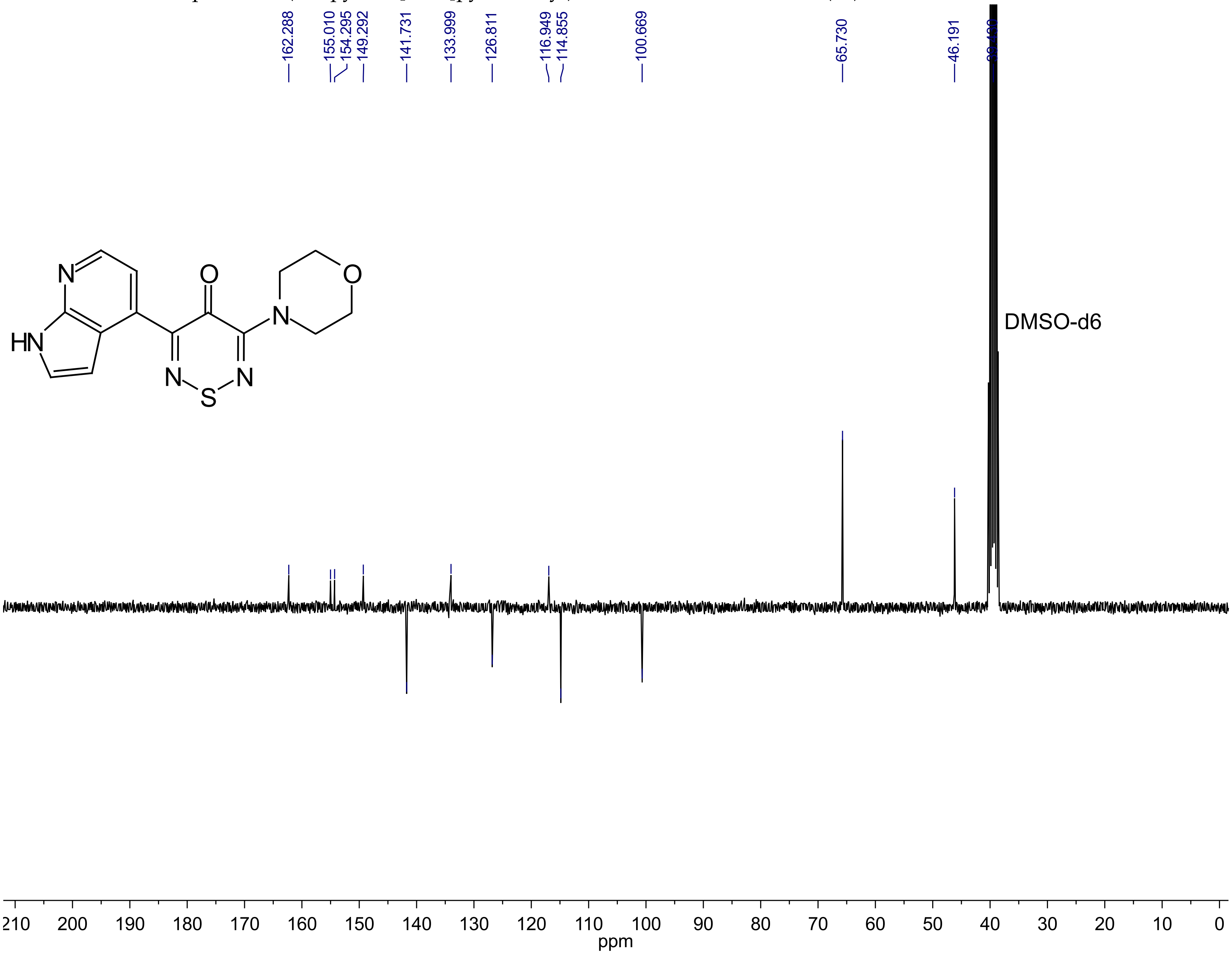

Current Data Parameters
NAME Andreas
EXPNO 139
PROCNO 1
F2 - Acquisition Parameters
Date_ 20180831
Time 18.13 h
INSTRUM spect
PROBHD Z104275_0375 (
PULPROG jmod
TD 65536
SOLVENT DMSO
NS 23338
DS 4
SWH 18115.941 Hz
FIDRES 0.552855 Hz
AQ 1.8087935 sec
RG 201.81
DW 27.600 usec
DE 6.50 usec
TE 298.3 K
CNST2 145.0000000
CNST11 1.0000000
D1 2.00000000 sec
D20 0.00689655 sec
TD0 1
SFO1 75.4752953 MHz
NUC1 13C
P1 10.00 usec
P2 20.00 usec
PLW1 40.05500031 W
SFO2 300.1312005 MHz
NUC2 1H
CPDPRG[2 waltz16
PCPD2 90.00 usec
PLW2 7.50000000 W
PLW12 0.18148001 W
F2 - Processing parameters
SI 32768
SF 75.4677913 MHz
WDW EM
SSB 0
LB 1.00 Hz
GB 0
PC 1.40

^{1}H NMR of 3-(4-Methylpiperazin-1-yl)-5-(1*H*-pyrrolo[2,3-*b*]pyridin-4-yl)-4*H*-1,2,6-thiadiazin-4-one (**20**)

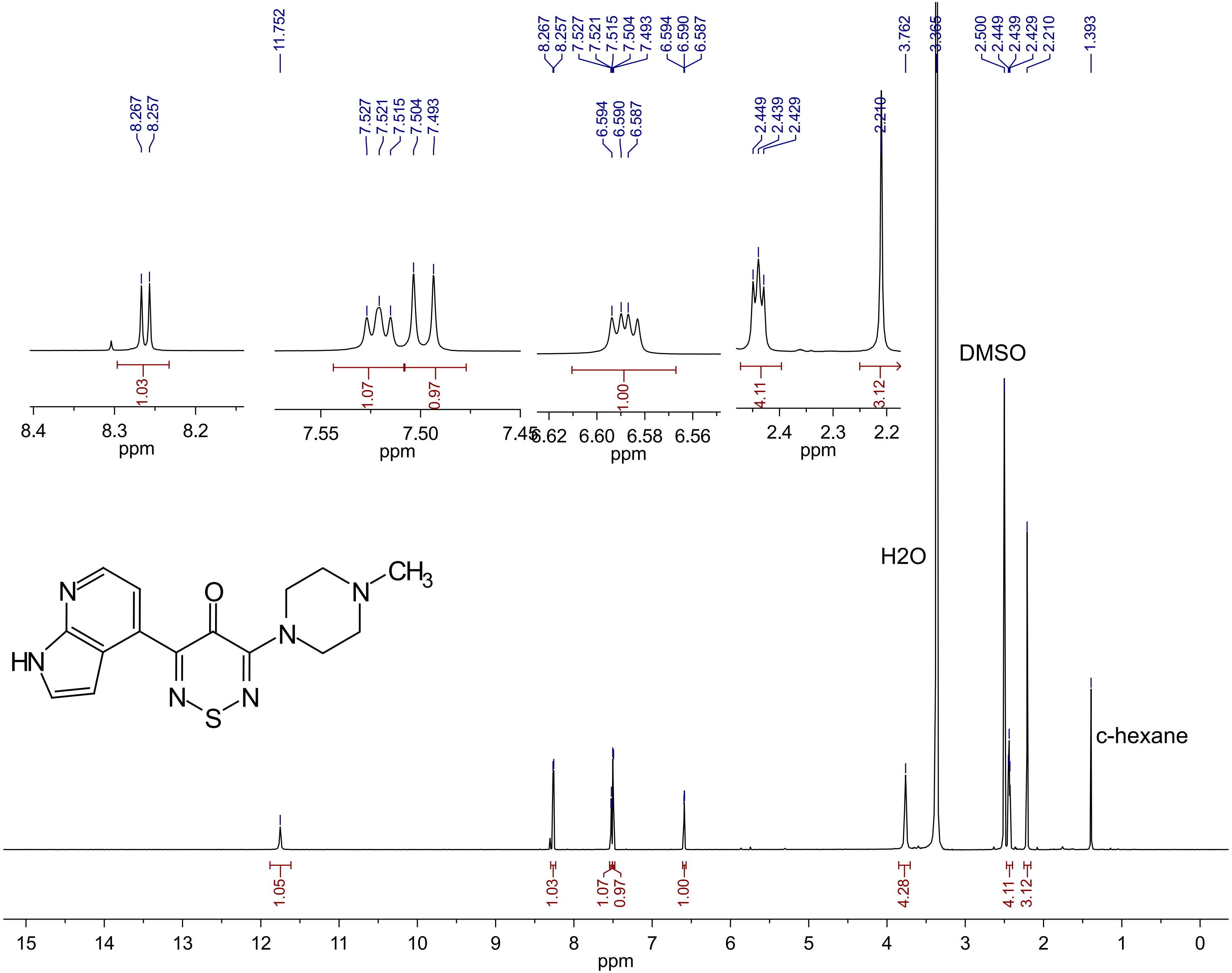

Current Data Parameters
NAME Kalogirou
EXPNO 633
PROCNO 1
F2 - Acquisition Parameters
Date_ 20190802
Time 16.12
INSTRUM spect
PROBHD 5 mm PABBO BB-
PULPROG zg30
TD 65536
SOLVENT DMSO
NS 16
DS 2
SWH 10000.000 Hz
FIDRES 0.152588 Hz
AQ 3.2767999 sec
RG 90.5
DW 50.000 usec
DE 6.50 usec
TE 298.4 K
D1 1.00000000 sec
TD0 1
======== CHANNEL f1 =
SFO1 500.0361158 MHz
NUC1 1H
P1 12.00 usec
PLW1 14.50000000 W
F2 - Processing parameters
SI 65536
SF 500.0330279 MHz
WDW EM
SSB 0
LB 0.30 Hz
GB 0
PC 1.00

^{13}C NMR of 3-(4-Methylpiperazin-1-yl)-5-(1*H*-pyrrolo[2,3-*b*]pyridin-4-yl)-4*H*-1,2,6-thiadiazin-4-one (**20**)

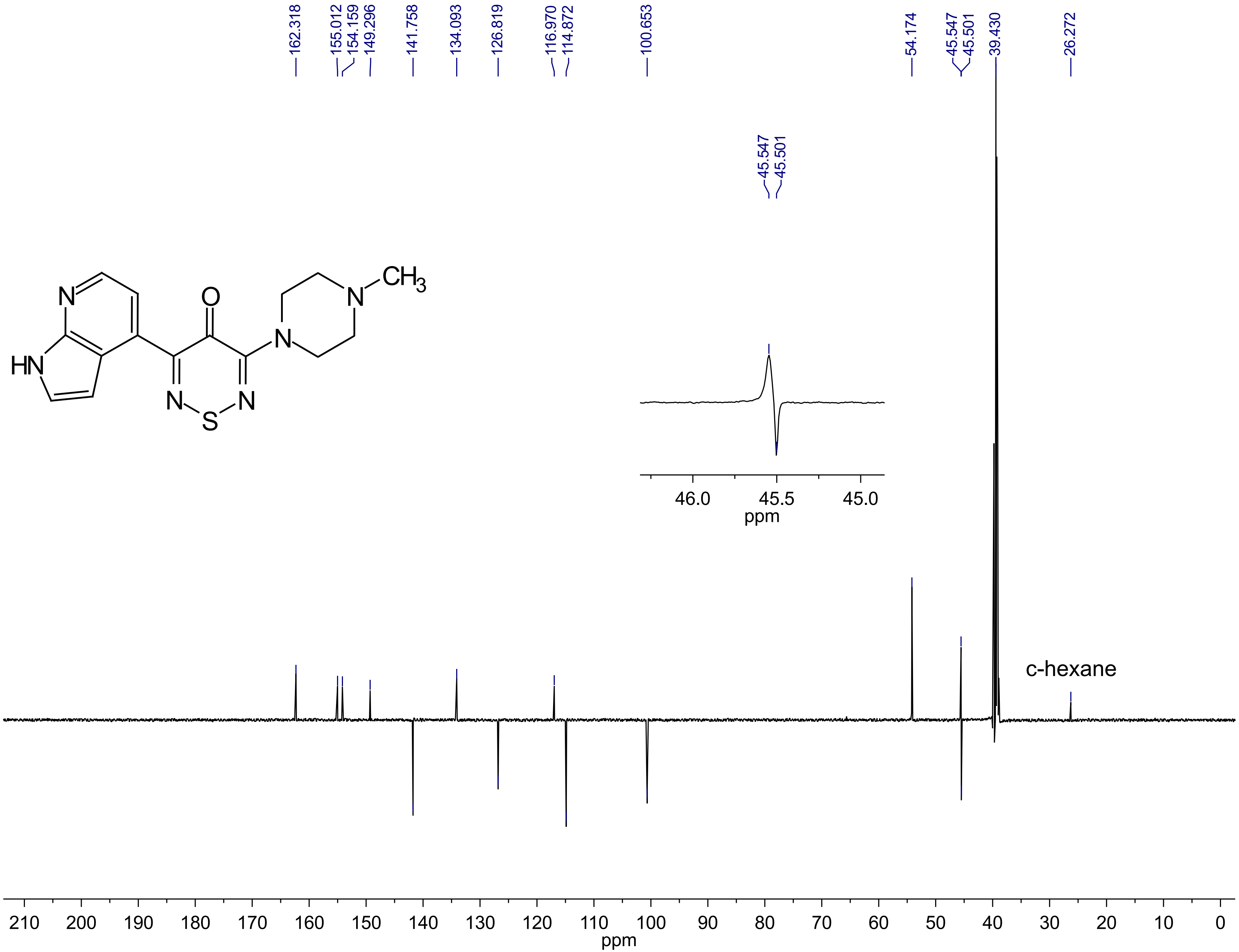

Current Data Parameters
NAME Kalogirou
EXPNO 634
PROCNO 1
F2 - Acquisition Parameters
Date_ 20190803
Time 12.39
INSTRUM spect
PROBHD 5 mm PABBO BB-
PULPROG jmod
TD 65536
SOLVENT DMSO
NS 19000
DS 4
SWH 29761.904 Hz
FIDRES 0.454131 Hz
AQ 1.1010048 sec
RG 2050
DW 16.800 usec
DE 6.50 usec
TE 298.5 K
CNST2 145.0000000
CNST11 1.0000000
D1 2.00000000 sec
D20 0.00689655 sec
TD0 1
======== CHANNEL f1 ========
SFO1 125.7459782 MHz
NUC1 13C
P1 9.20 usec
P2 18.40 usec
PLW1 140.00000000 W
======== CHANNEL f2 ========
SFO2 500.0350280 MHz
NUC2 1H
CPDPRG[2 waltz16
PCPD2 80.00 usec
PLW2 14.50000000 W
PLW12 0.32624999 W
F2 - Processing parameters
SI 32768
SF 125.7334732 MHz
WDW EM
SSB 0
LB 1.00 Hz
GB 0
PC 1.40

^{1}H NMR of 3-((1*H*-pyrrolo[2,3-*b*]pyridin-4-yl)amino)-5-(4-methylpiperazin-1-yl)-4*H*-1,2,6-thiadiazin-4-one (**21**)

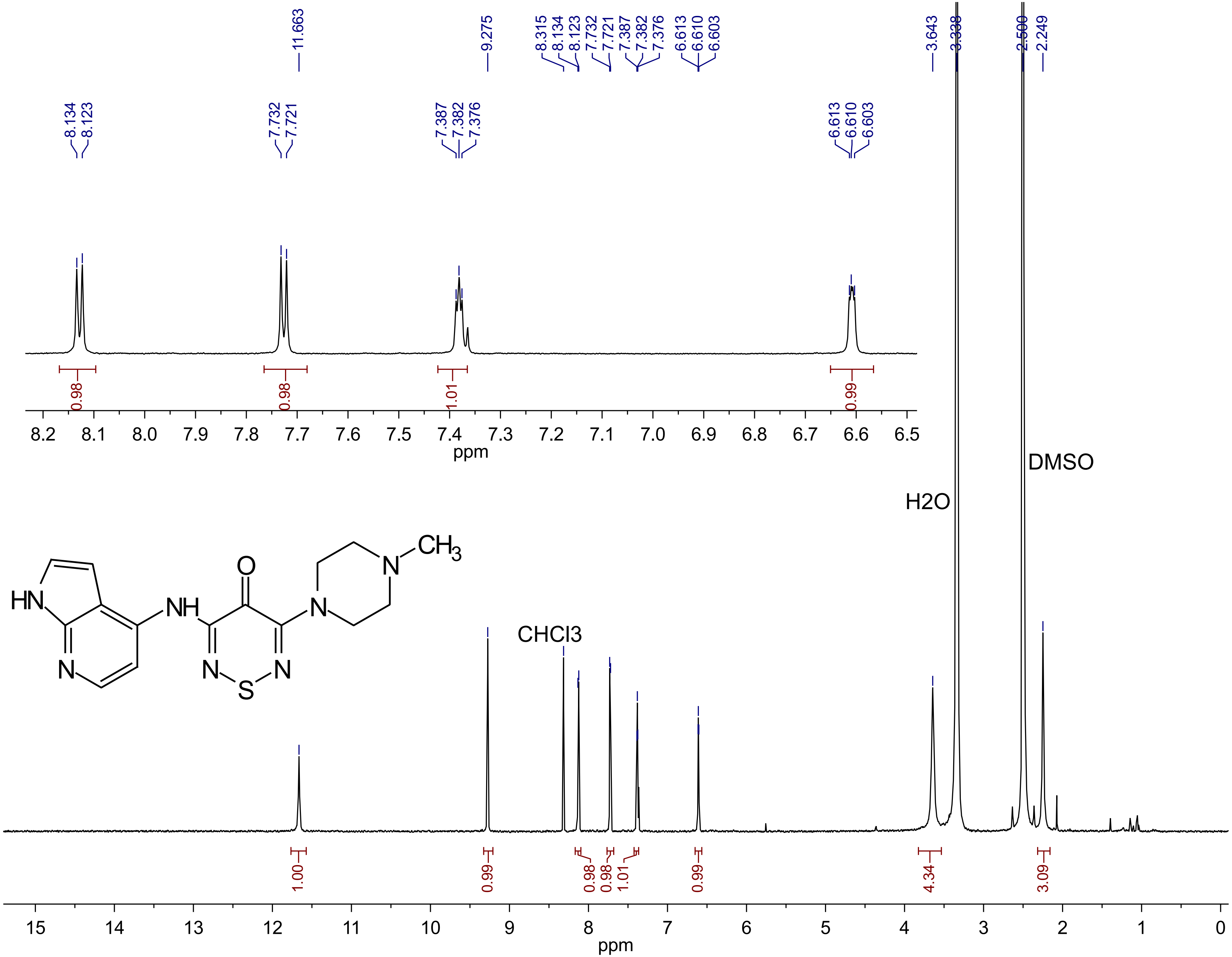

Current Data Parameters
NAME Kalogirou
EXPNO 488
PROCNO 1

F2 - Acquisition Parameters
Date_ 20171224
Time 11.04
INSTRUM spect
PROBHD 5 mm PABBO BB-
PULPROG zg30
TD 65536
SOLVENT DMSO
NS 16
DS 2
SWH 10000.000 Hz
FIDRES 0.152588 Hz
AQ 3.2767999 sec
RG 144
DW 50.000 usec
DE 6.50 usec
TE 295.9 K
D1 1.00000000 sec
TD0 1

======== CHANNEL f1 ========
SFO1 500.0361158 MHz
NUC1 1H
P1 12.00 usec
PLW1 14.50000000 W

F2 - Processing parameters
SI 65536
SF 500.0330315 MHz
WDW EM
SSB 0
LB 0.30 Hz
GB 0
PC 1.00

^{13}C NMR of 3-((1*H*-pyrrolo[2,3-*b*]pyridin-4-yl)amino)-5-(4-methylpiperazin-1-yl)-4*H*-1,2,6-thiadiazin-4-one (**21**)

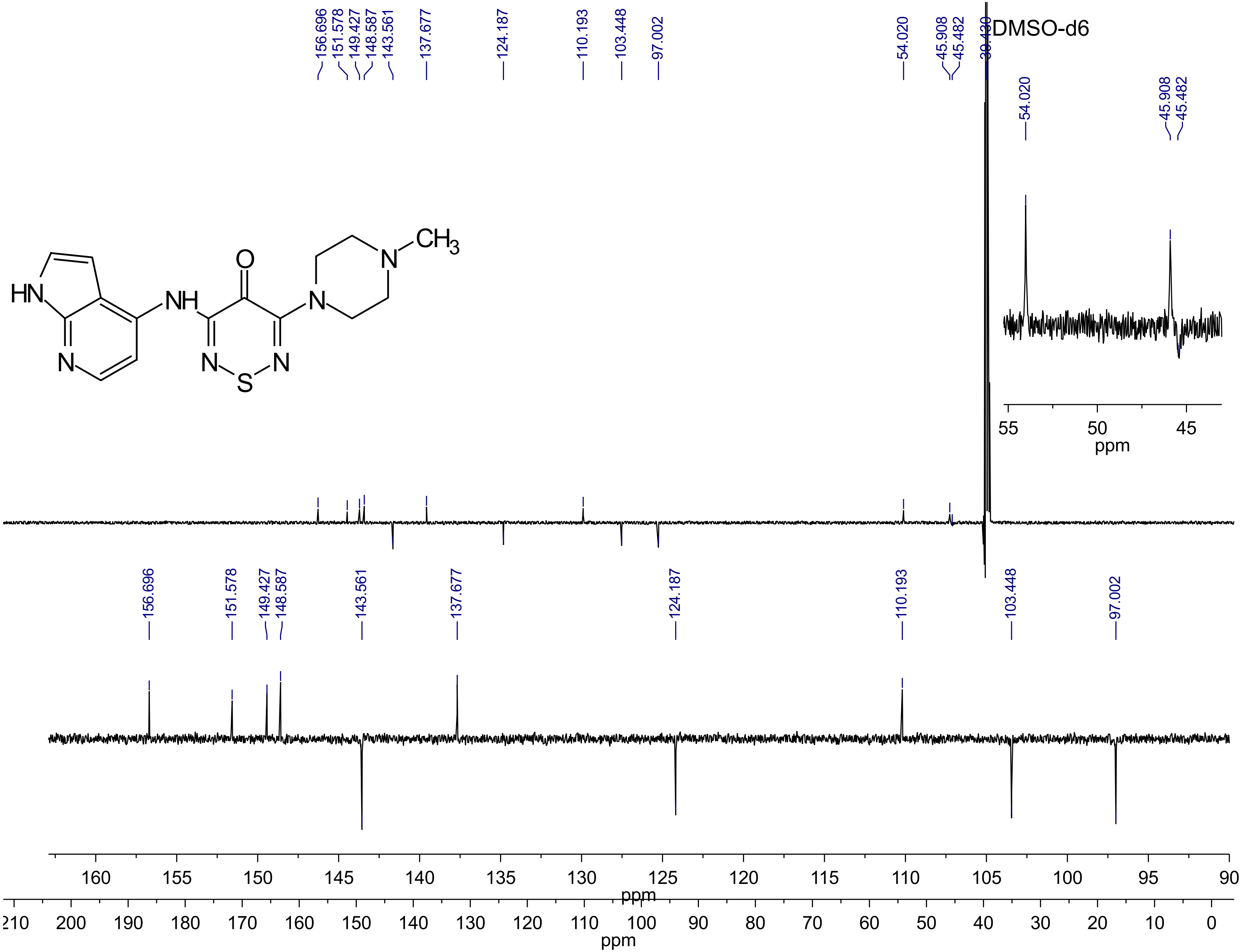

Current Data Parameters
NAME Kalogirou
EXPNO 489
PROCNO 1
F2 - Acquisition Parameters
Date_ 20171225
Time 2.19
INSTRUM spect
PROBHD 5 mm PABBO BB-
PULPROG jmod
TD 65536
SOLVENT DMSO
NS 17408
DS 4
SWH 29761.904 Hz
FIDRES 0.454131 Hz
AQ 1.1010048 sec
RG 2050
DW 16.800 usec
DE 6.50 usec
TE 297.3 K
CNST2 145.0000000
CNST11 1.0000000
D1 2.00000000 sec
D20 0.00689655 sec
TD0 1
======== CHANNEL f1 ========
SFO1 125.7459782 MHz
NUC1 13C
P1 9.00 usec
P2 18.00 usec
PLW1 140.00000000 W
======== CHANNEL f2 ========
SFO2 500.0350280 MHz
NUC2 1H
CPDPRG[2 waltz16
PCPD2 80.00 usec
PLW2 14.50000000 W
PLW12 0.32624999 W
F2 - Processing parameters
SI 32768
SF 125.7334738 MHz
WDW EM
SSB 0
LB 1.00 Hz
GB 0
PC [illegible]

^{1}H NMR of 3-((1*H*-indazol-5-yl)amino)-5-(4-methylpiperazin-1-yl)-4*H*-1,2,6-thiadiazin-4-one (**22**)

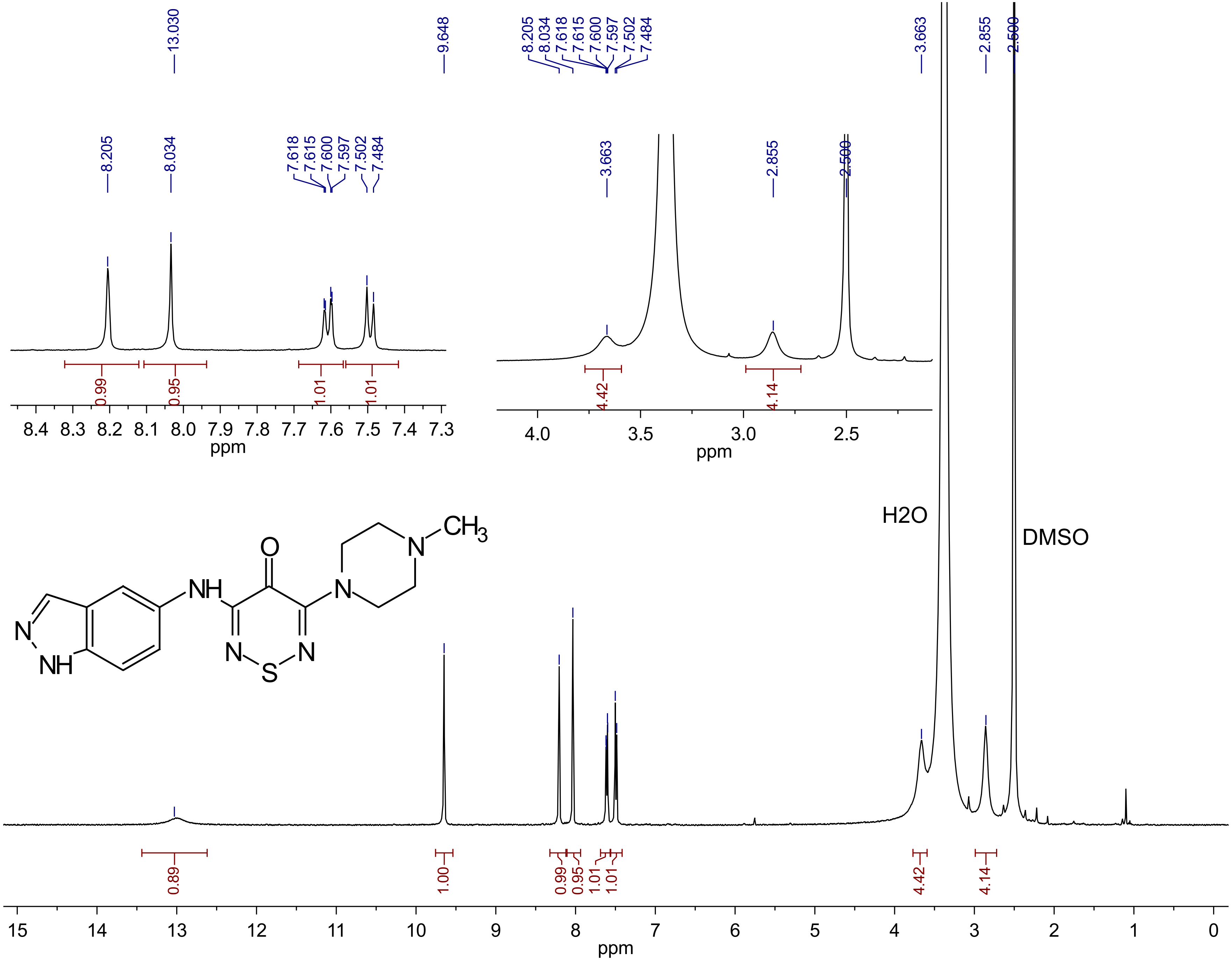

Current Data Parameters
NAME Kalogirou
EXPNO 572
PROCNO 1

F2 - Acquisition Parameters
Date_ 20181031
Time 15.46
INSTRUM spect
PROBHD 5 mm PABBO BB-
PULPROG zg30
TD 65536
SOLVENT DMSO
NS 16
DS 2
SWH 10000.000 Hz
FIDRES 0.152588 Hz
AQ 3.2767999 sec
RG 101
DW 50.000 usec
DE 6.50 usec
TE 294.8 K
D1 1.00000000 sec
TD0 1

======== CHANNEL f1 ========
SFO1 500.0361158 MHz
NUC1 1H
P1 12.00 usec
PLW1 14.80000019 W

F2 - Processing parameters
SI 65536
SF 500.0330315 MHz
WDW EM
SSB 0
LB 0.30 Hz
GB 0
PC 1.00

^{13}C NMR of 3-((1*H*-indazol-5-yl)amino)-5-(4-methylpiperazin-1-yl)-4*H*-1,2,6-thiadiazin-4-one (**22**)

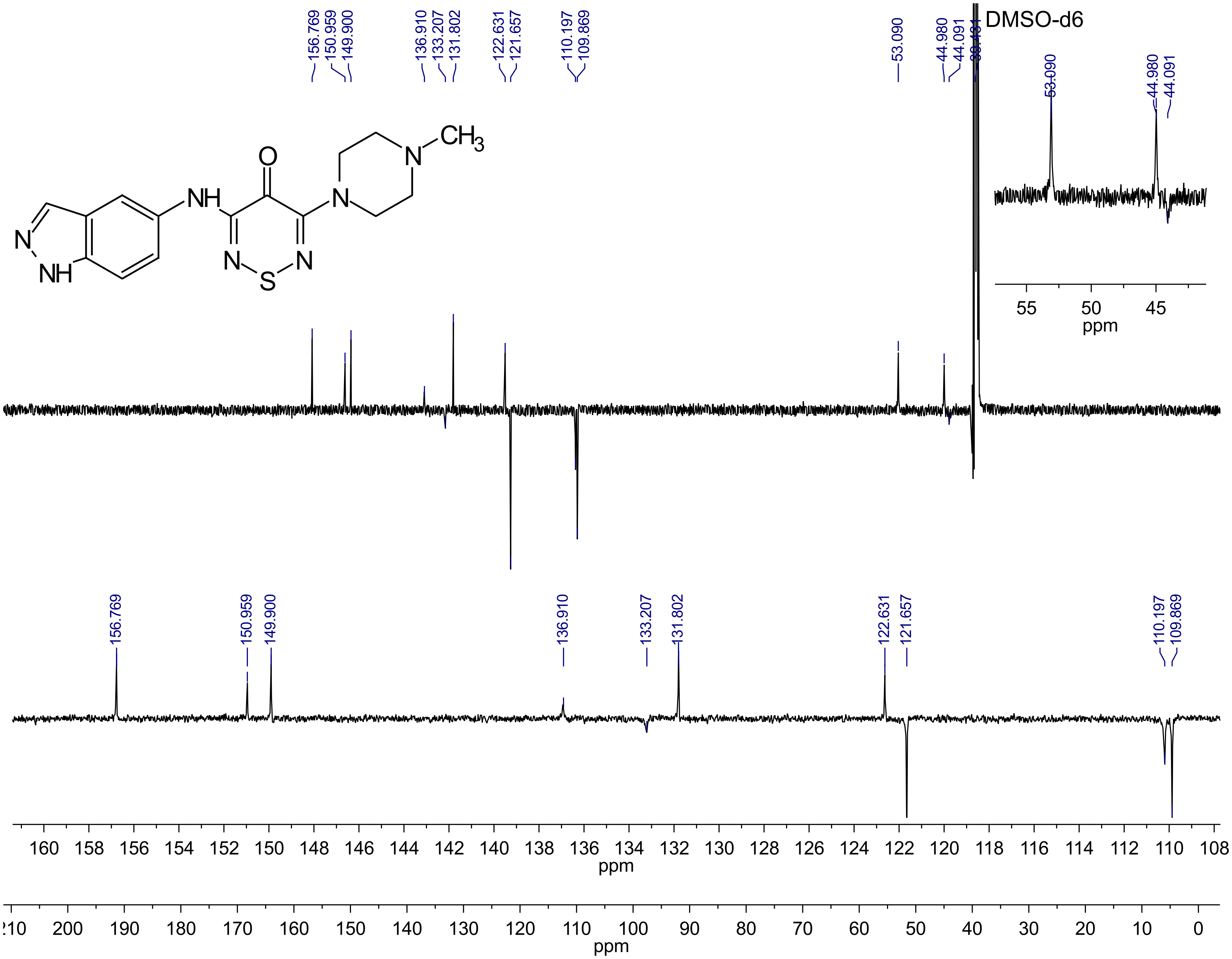

Current Data Parameters
NAME Kalogirou
EXPNO 571
PROCNO 1

F2 - Acquisition Parameters
Date_ 20181031
Time 9.13
INSTRUM spect
PROBHD 5 mm PABBO BB-
PULPROG jmod
TD 65536
SOLVENT DMSO
NS 16000
DS 4
SWH 29761.904 Hz
FIDRES 0.454131 Hz
AQ 1.1010048 sec
RG 2050
DW 16.800 usec
DE 6.50 usec
TE 296.6 K
CNST2 145.0000000
CNST11 1.0000000
D1 2.00000000 sec
D20 0.00689655 sec
TD0 1

======== CHANNEL f1 ========
SFO1 125.7459782 MHz
NUC1 13C
P1 9.40 usec
P2 18.80 usec
PLW1 140.00000000 W

======== CHANNEL f2 ========
SFO2 500.0350280 MHz
NUC2 1H
CPDPRG[2 waltz16
PCPD2 80.00 usec
PLW2 14.80000019 W
PLW12 0.33300000 W

F2 - Processing parameters
SI 32768
SF 125.7334711 MHz
WDW EM
SSB 0
LB 1.00 Hz
GB 0
PC 1.40

^{1}H NMR of 3-((1*H*-indazol-5-yl)amino)-5-(3-hydroxyphenyl)-4*H*-1,2,6-thiadiazin-4-one (**23**)

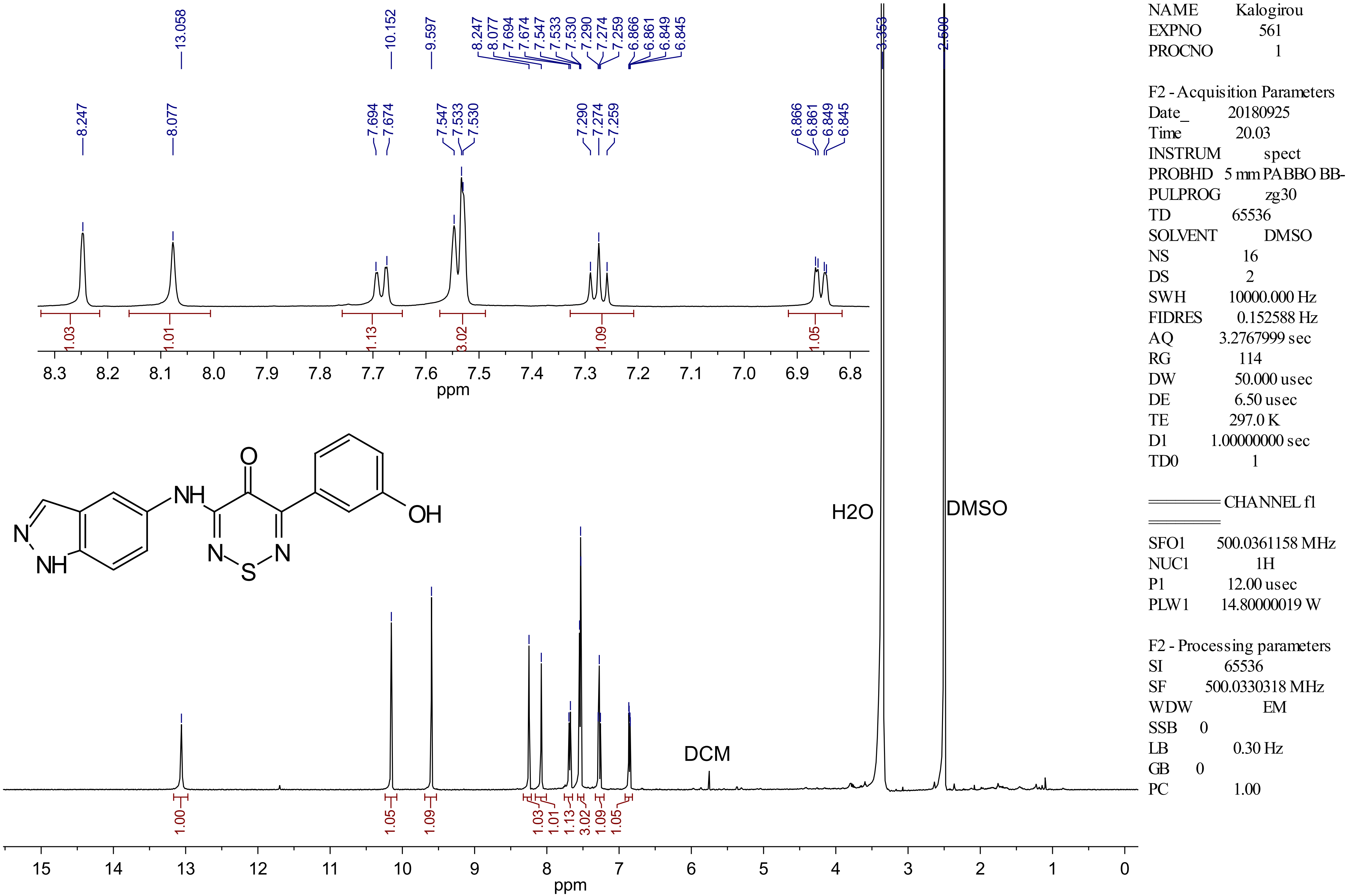

^{1}H NMR of 3-((1*H*-indazol-5-yl)amino)-5-(3-hydroxyphenyl)-4*H*-1,2,6-thiadiazin-4-one (**23**)

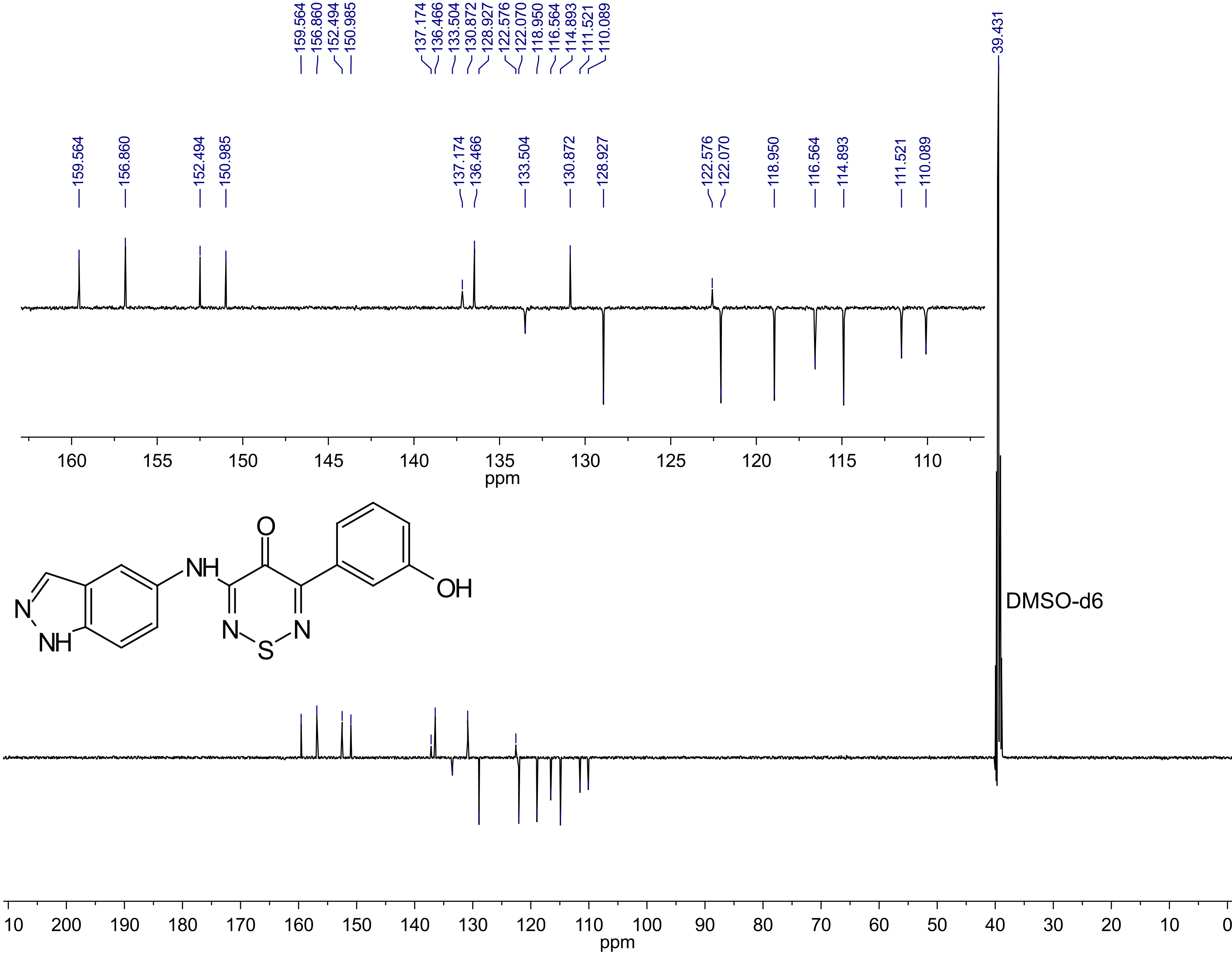

Current Data Parameters
NAME Kalogirou
EXPNO 562
PROCNO 1
F2 - Acquisition Parameters
Date_ 20180926
Time 8.53
INSTRUM spect
PROBHD 5 mm PABBO BB-
PULPROG jmod
TD 65536
SOLVENT DMSO
NS 14500
DS 4
SWH 29761.904 Hz
FIDRES 0.454131 Hz
AQ 1.1010048 sec
RG 2050
DW 16.800 usec
DE 6.50 usec
TE 299.7 K
CNST2 145.0000000
CNST11 1.0000000
D1 2.00000000 sec
D20 0.00689655 sec
TD0 1

======== CHANNEL f1 ========

SFO1 125.7459782 MHz
NUC1 13C
P1 9.40 usec
P2 18.80 usec
PLW1 140.00000000 W

======== CHANNEL f2 ========

SFO2 500.0350280 MHz
NUC2 1H
CPDPRG[2 waltz16
PCPD2 80.00 usec
PLW2 14.80000019 W
PLW12 0.33300000 W
F2 - Processing parameters
SI 32768
SF 125.7334757 MHz
WDW EM
SSB 0
LB 1.00 Hz
GB 0
PC 1.40

^{1}H NMR of 3-((1*H*-indazol-5-yl)amino)-5-(5-hydroxy-2-methylphenyl)-4*H*-1,2,6-thiadiazin-4-one (**24**)

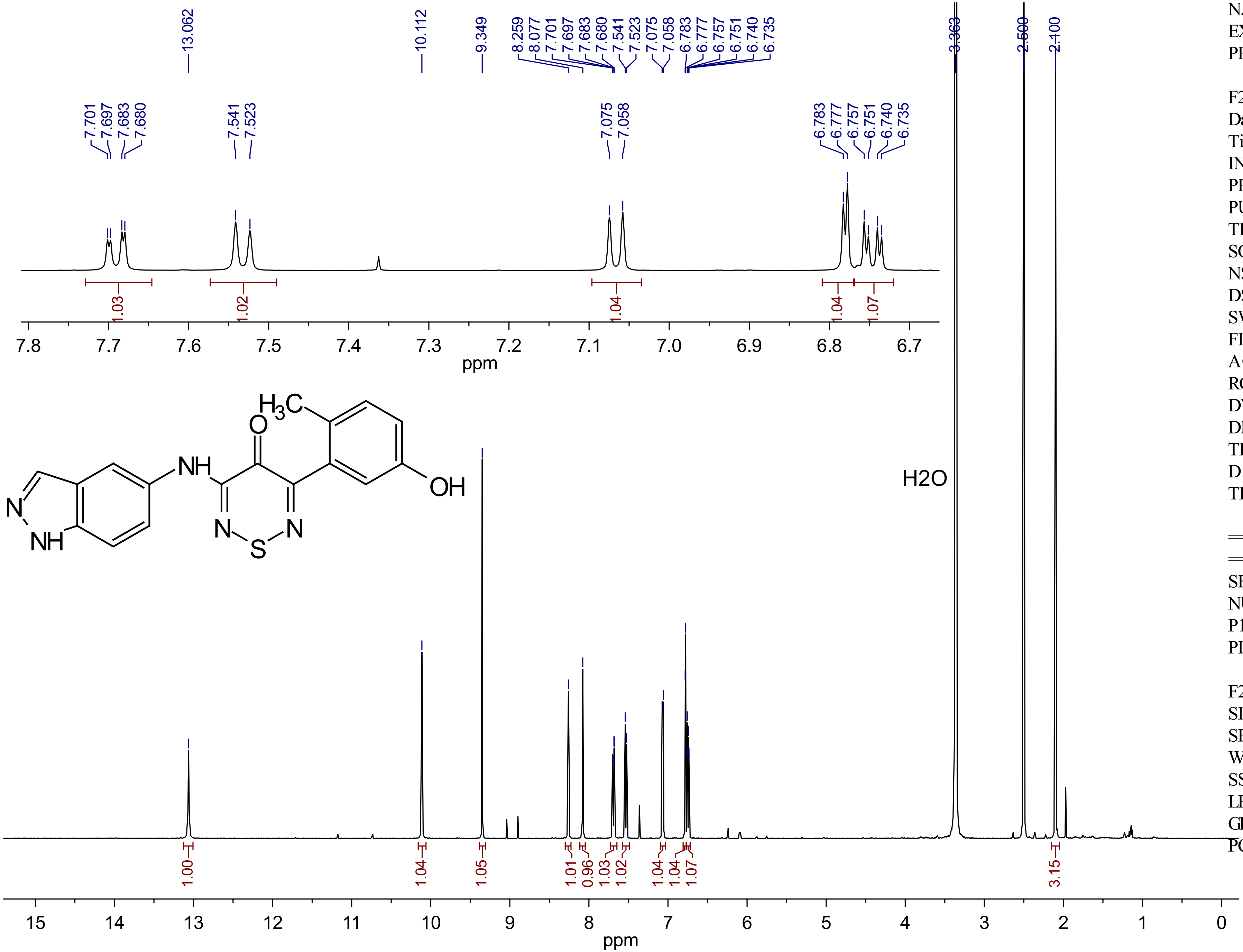

Current Data Parameters
NAME Kalogirou
EXPNO 564
PROCNO 1

F2 - Acquisition Parameters
Date_ 20181016
Time 19.13
INSTRUM spect
PROBHD 5 mm PABBO BB-
PULPROG zg30
TD 65536
SOLVENT DMSO
NS 16
DS 2
SWH 10000.000 Hz
FIDRES 0.152588 Hz
AQ 3.2767999 sec
RG 114
DW 50.000 usec
DE 6.50 usec
TE 295.6 K
D1 1.00000000 sec
TD0 1

CHANNEL f1

SFO1 500.0361158 MHz
NUC1 1H
P1 12.00 usec
PLW1 14.80000019 W

F2 - Processing parameters
SI 65536
SF 500.0330318 MHz
WDW EM
SSB 0
LB 0.30 Hz
GB 0
PC 1.00

^{13}C NMR of 3-((1*H*-indazol-5-yl)amino)-5-(5-hydroxy-2-methylphenyl)-4*H*-1,2,6-thiadiazin-4-one (**24**)

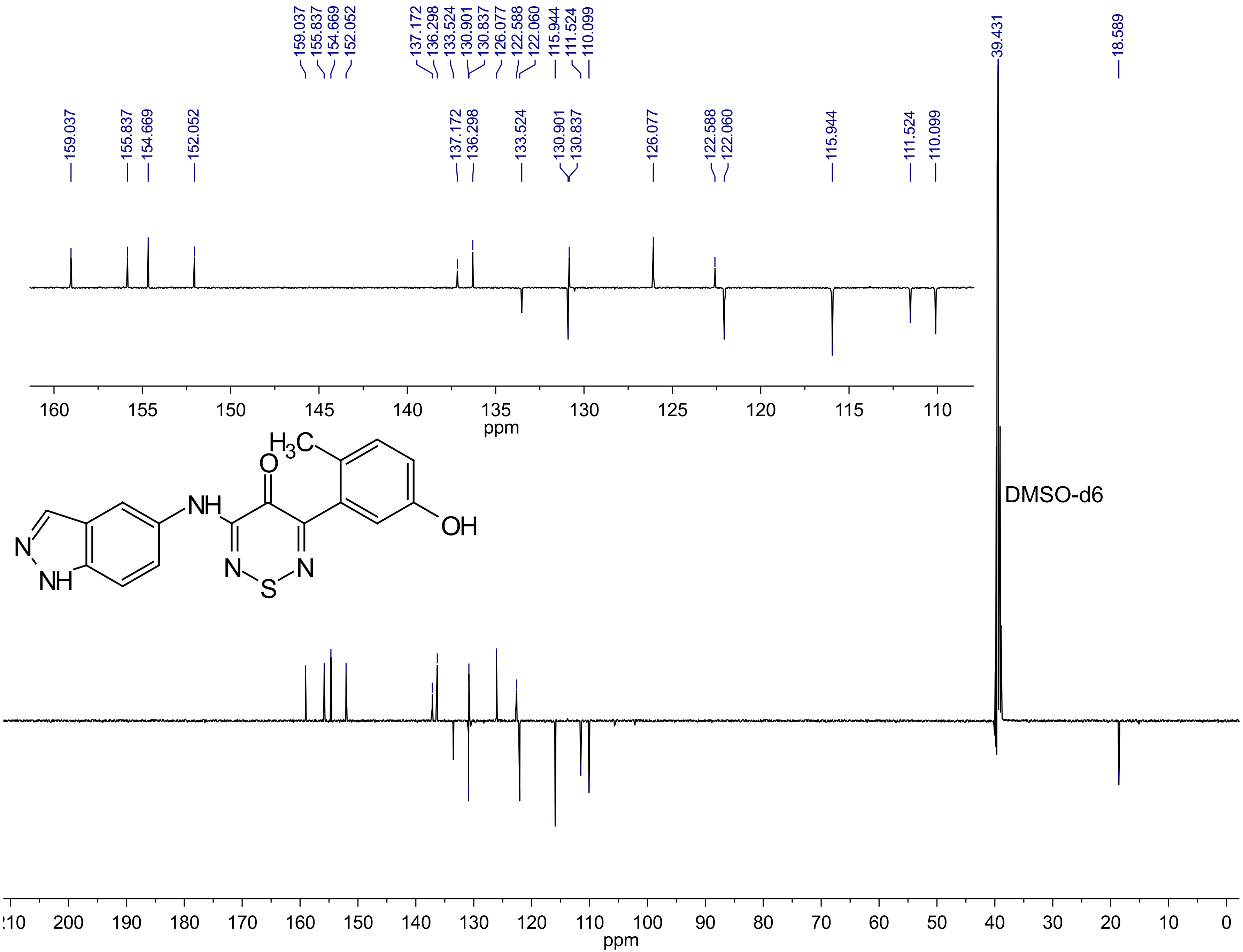

Current Data Parameters	
NAME	Kalogirou
EXPNO	565
PROCNO	1
F2 - Acquisition Parameters	
Date_	20181017
Time	9.11
INSTRUM	spect
PROBHD	5 mm PABBO BB-
PULPROG	jmod
TD	65536
SOLVENT	DMSO
NS	15800
DS	4
SWH	29761.904 Hz
FIDRES	0.454131 Hz
AQ	1.1010048 sec
RG	2050
DW	16.800 usec
DE	6.50 usec
TE	298.0 K
CNST2	145.0000000
CNST11	1.0000000
D1	2.00000000 sec
D20	0.00689655 sec
TD0	1
======== CHANNEL f1 ========	
SFO1	125.7459782 MHz
NUC1	13C
P1	9.40 usec
P2	18.80 usec
PLW1	140.00000000 W
======== CHANNEL f2 ========	
SFO2	500.0350280 MHz
NUC2	1H
CPDPRG[2	waltz16
PCPD2	80.00 usec
PLW2	14.80000019 W
PLW12	0.33300000 W
F2 - Processing parameters	
SI	32768
SF	125.7334731 MHz
WDW	EM
SSB	0
LB	1.00 Hz
GB	0
PC	1.40

^{1}H NMR of 3-((1*H*-indazol-5-yl)amino)-5-(3-methoxyphenyl)-4*H*-1,2,6-thiadiazin-4-one (**25**)

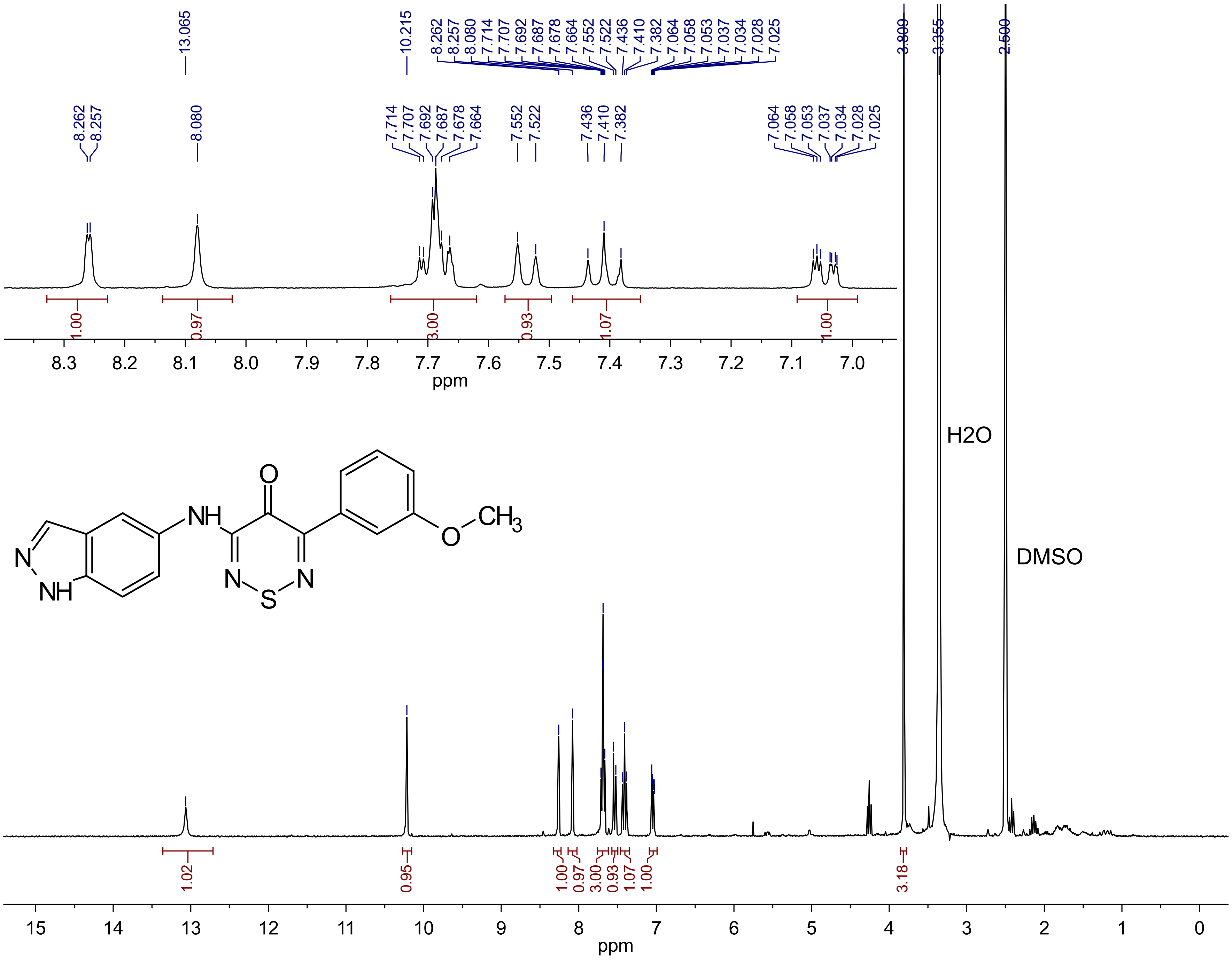

Current Data Parameters
NAME Andreas
EXPNO 145
PROCNO 1
F2 - Acquisition Parameters
Date_ 20180928
Time 15.28 h
INSTRUM spect
PROBHD Z104275_0375 (
PULPROG zg30
TD 65536
SOLVENT DMSO
NS 16
DS 2
SWH 6009.615 Hz
FIDRES 0.183399 Hz
AQ 5.4525952 sec
RG 201.81
DW 83.200 usec
DE 6.50 usec
TE 295.8 K
D1 1.00000000 sec
TD0 1
SFO1 300.1318533 MHz
NUC1 1H
P1 14.00 usec
PLW1 7.50000000 W
F2 - Processing parameters
SI 65536
SF 300.1300025 MHz
WDW EM
SSB 0
LB 0.30 Hz
GB 0
PC 1.00

^{13}C NMR of 3-((1*H*-indazol-5-yl)amino)-5-(3-methoxyphenyl)-4*H*-1,2,6-thiadiazin-4-one (**25**)

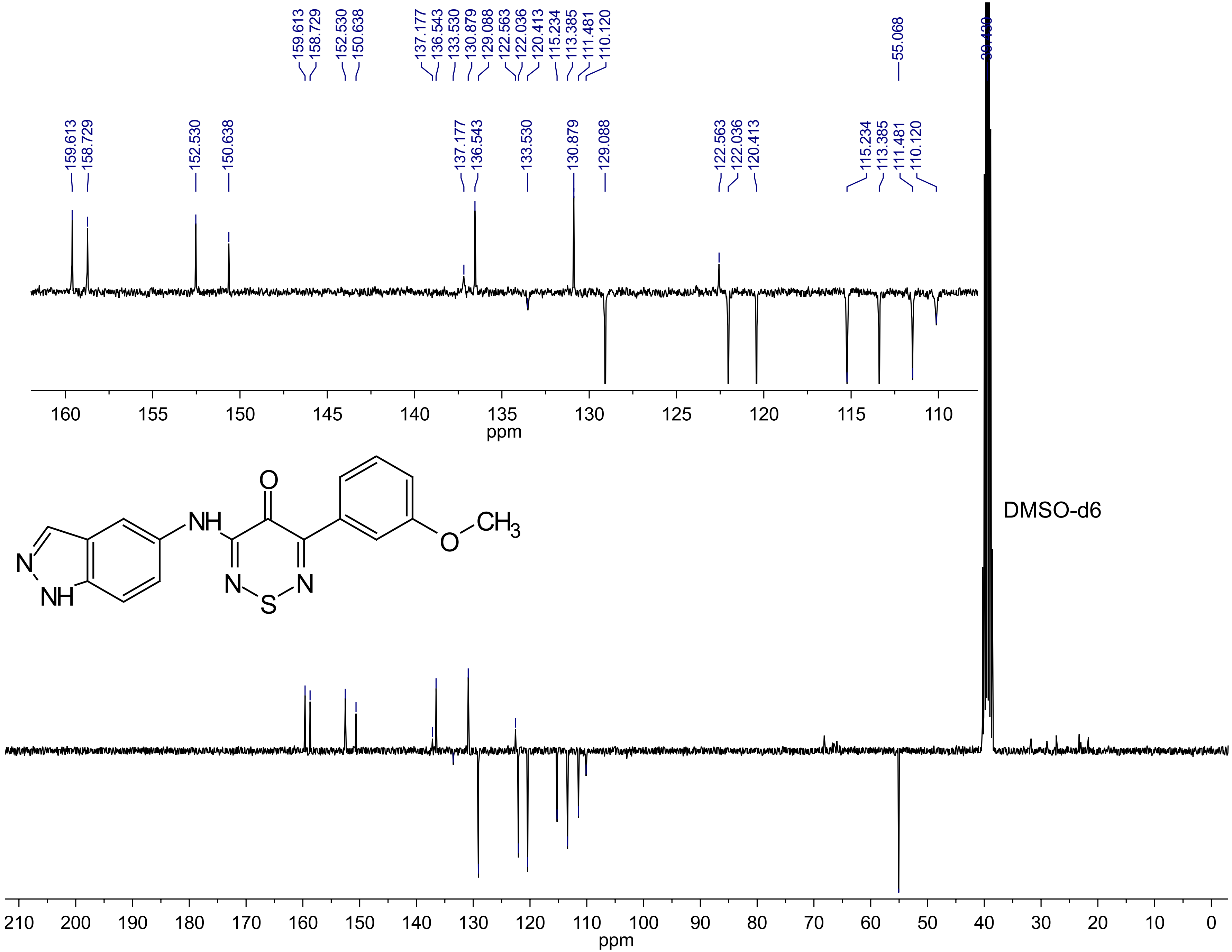

Current Data Parameters
NAME Andreas
EXPNO 146
PROCNO 1
F2 - Acquisition Parameters
Date_ 20180929
Time 15.10 h
INSTRUM spect
PROBHD Z104275_0375 (
PULPROG jmod
TD 65536
SOLVENT DMSO
NS 22000
DS 4
SWH 18115.941 Hz
FIDRES 0.552855 Hz
AQ 1.8087935 sec
RG 201.81
DW 27.600 usec
DE 6.50 usec
TE 296.2 K
CNST2 145.0000000
CNST11 1.0000000
D1 2.00000000 sec
D20 0.00689655 sec
TD0 1
SFO1 75.4752953 MHz
NUC1 13C
P1 10.00 usec
P2 20.00 usec
PLW1 40.05500031 W
SFO2 300.1312005 MHz
NUC2 1H
CPDPRG[2 waltz16
PCPD2 90.00 usec
PLW2 7.50000000 W
PLW12 0.18148001 W
F2 - Processing parameters
SI 32768
SF 75.4677905 MHz
WDW EM
SSB 0
LB 1.00 Hz
GB 0
PC 1.40

^{1}H NMR of 3-((1*H*-indazol-5-yl)amino)-5-(2-fluoropyridin-4-yl)-4*H*-1,2,6-thiadiazin-4-one (**26**)

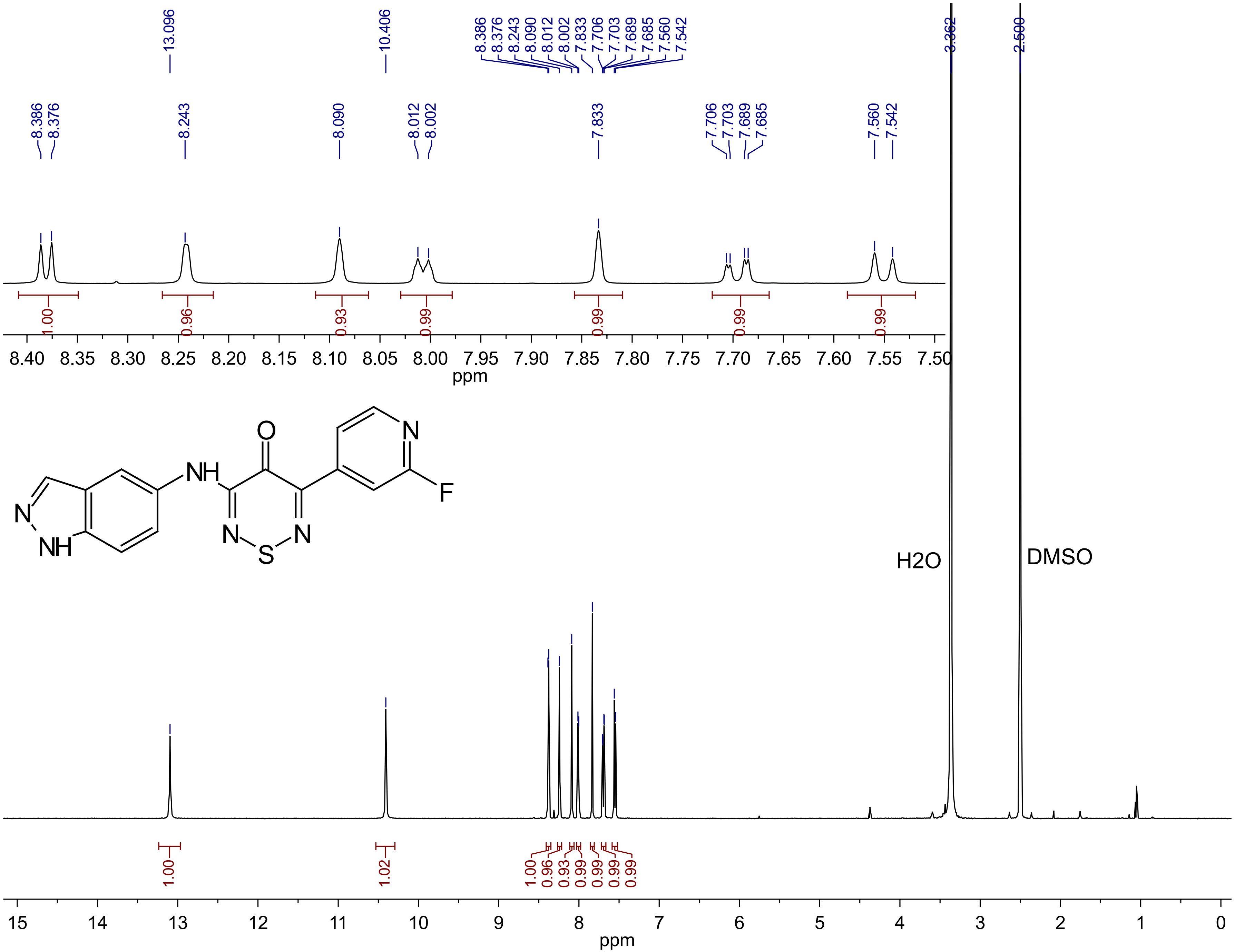

Current Data Parameters
NAME Kalogirou
EXPNO 568
PROCNO 1

F2 - Acquisition Parameters
Date_ 20181026
Time 15.48
INSTRUM spect
PROBHD 5 mm PABBO BB-
PULPROG zg30
TD 65536
SOLVENT DMSO
NS 16
DS 2
SWH 10000.000 Hz
FIDRES 0.152588 Hz
AQ 3.2767999 sec
RG 114
DW 50.000 usec
DE 6.50 usec
TE 295.2 K
D1 1.00000000 sec
TD0 1

======== CHANNEL f1 ========
SFO1 500.0361158 MHz
NUC1 1H
P1 12.00 usec
PLW1 14.80000019 W

F2 - Processing parameters
SI 65536
SF 500.0330319 MHz
WDW EM
SSB 0
LB 0.30 Hz
GB 0
PC 1.00

^{13}C NMR of 3-((1*H*-indazol-5-yl)amino)-5-(2-fluoropyridin-4-yl)-4*H*-1,2,6-thiadiazin-4-one (**26**)

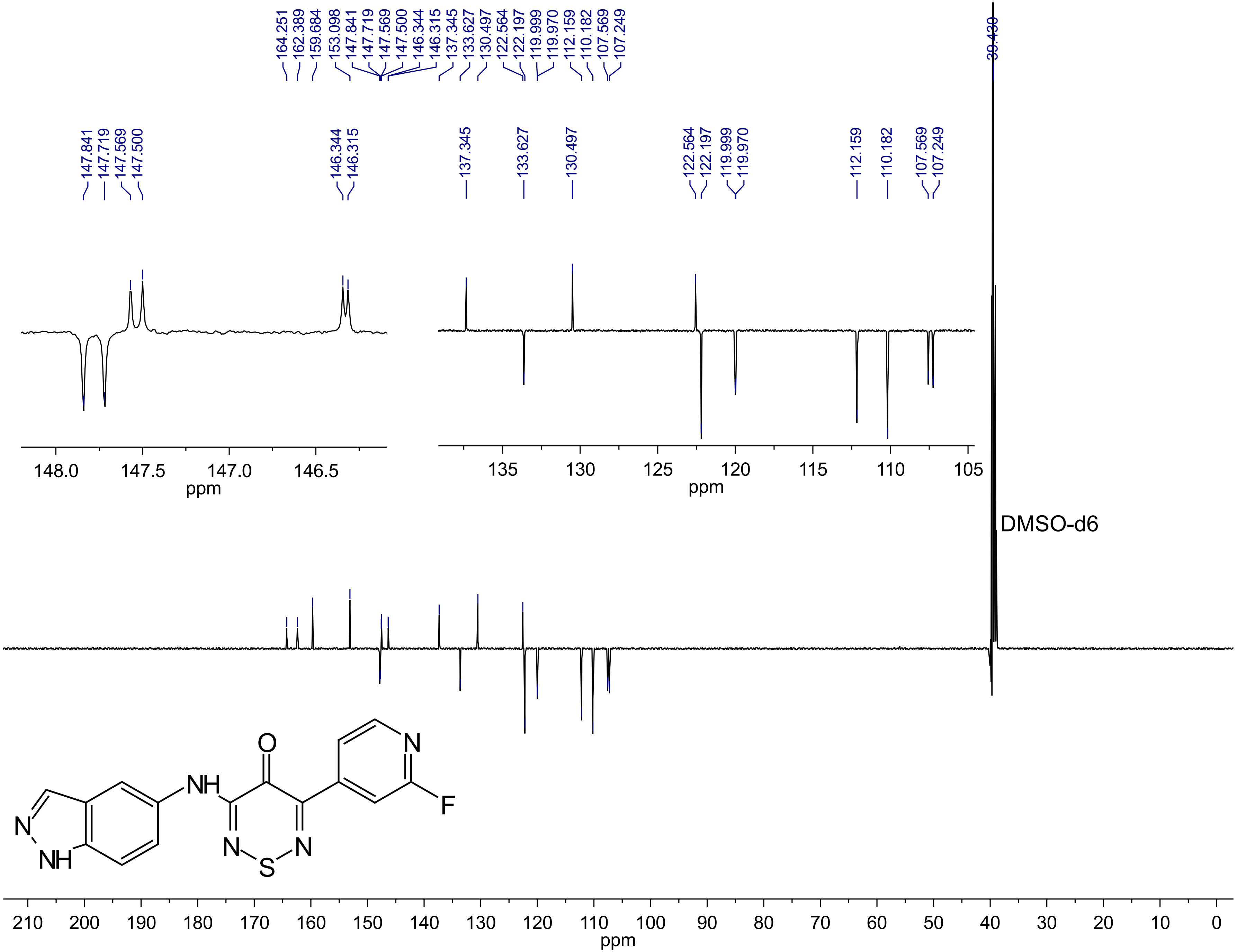

Current Data Parameters
NAME Kalogirou
EXPNO 569
PROCNO 1
F2 - Acquisition Parameters
Date_ 20181029
Time 9.06
INSTRUM spect
PROBHD 5 mm PABBO BB-
PULPROG jmod
TD 65536
SOLVENT DMSO
NS 27500
DS 4
SWH 29761.904 Hz
FIDRES 0.454131 Hz
AQ 1.1010048 sec
RG 2050
DW 16.800 usec
DE 6.50 usec
TE 296.9 K
CNST2 145.0000000
CNST11 1.0000000
D1 2.00000000 sec
D20 0.00689655 sec
TD0 1
======== CHANNEL f1 ========
SFO1 125.7459782 MHz
NUC1 13C
P1 9.40 usec
P2 18.80 usec
PLW1 140.00000000 W
======== CHANNEL f2 ========
SFO2 500.0350280 MHz
NUC2 1H
CPDPRG[2 waltz16
PCPD2 80.00 usec
PLW2 14.80000019 W
PLW12 0.33300000 W
F2 - Processing parameters
SI 32768
SF 125.7334716 MHz
WDW EM
SSB 0
LB 1.00 Hz
GB 0
PC 1.40
